# Study of Biological Activities and ADMET-Related Properties of Novel Chlorinated *N*-arylcinnamamides

**DOI:** 10.3390/ijms23063159

**Published:** 2022-03-15

**Authors:** Tomas Strharsky, Dominika Pindjakova, Jiri Kos, Lucia Vrablova, Hana Michnova, Jan Hosek, Nicol Strakova, Veronika Lelakova, Lenka Leva, Lenka Kavanova, Michal Oravec, Alois Cizek, Josef Jampilek

**Affiliations:** 1 Regional Centre of Advanced Technologies and Materials, Czech Advanced Technology and Research Institute, Palacky University, Slechtitelu 27, 783 71 Olomouc, Czech Republic; strharsky.t@gmail.com (T.S.); michnova.hana@gmail.com (H.M.); jan.hosek@upol.cz (J.H.); josef.jampilek@gmail.com (J.J.); 2 Department of Analytical Chemistry, Faculty of Natural Sciences, Comenius University, Ilkovicova 6, 842 15 Bratislava, Slovakia; pindjakova.dominika@gmail.com (D.P.); lucia.vrablova26@gmail.com (L.V.); 3 Department of Biochemistry, Faculty of Medicine, Masaryk University, Kamenice 5, 625 00 Brno, Czech Republic; 4 Department of Pharmacology and Toxicology, Veterinary Research Institute, Hudcova 296/70, 621 00 Brno, Czech Republic; nicol.strakova@vri.cz (N.S.); veronika.lelakova@vri.cz (V.L.); 5 Department of Infectious Diseases and Preventive Medicine, Veterinary Research Institute, Hudcova 296/70, 621 00 Brno, Czech Republic; lenka.leva@vri.cz (L.L.); lenka.kavanova@vri.cz (L.K.); 6 Global Change Research Institute CAS, Belidla 986/4a, 60300 Brno, Czech Republic; oravec.m@czechglobe.cz; 7 Department of Infectious Diseases and Microbiology, Faculty of Veterinary Medicine, University of Veterinary Sciences Brno, Palackeho 1946/1, 612 42 Brno, Czech Republic; cizeka@vfu.cz

**Keywords:** cinnamamides, antimicrobial activity, cytotoxicity, lipophilicity, structure-activity relationships

## Abstract

A series of eighteen 4-chlorocinnamanilides and eighteen 3,4-dichlorocinnamanilides were designed, prepared and characterized. All compounds were evaluated for their activity against gram-positive bacteria and against two mycobacterial strains. Viability on both cancer and primary mammalian cell lines was also assessed. The lipophilicity of the compounds was experimentally determined and correlated together with other physicochemical properties of the prepared derivatives with biological activity. 3,4-Dichlorocinnamanilides showed a broader spectrum of action and higher antibacterial efficacy than 4-chlorocinnamanilides; however, all compounds were more effective or comparable to clinically used drugs (ampicillin, isoniazid, rifampicin). Of the thirty-six compounds, six derivatives showed submicromolar activity against *Staphylococcus aureus* and clinical isolates of methicillin-resistant *S. aureus* (MRSA). (2*E*)-*N*-[3,5-bis(trifluoromethyl)phenyl]- 3-(4-chlorophenyl)prop-2-enamide was the most potent in series **1**. (2*E*)-*N*-[3,5-bis(Trifluoromethyl)phenyl]-3-(3,4-dichlorophenyl)prop-2-enamide, (2*E*)-3-(3,4-dichlorophenyl)-*N*-[3-(trifluoromethyl)phenyl]prop-2-enamide, (2*E*)-3-(3,4-dichloro- phenyl)-*N*-[4-(trifluoromethyl)phenyl]prop-2-enamide and (2*E*)-3-(3,4-dichlorophenyl)- *N*-[4-(trifluoromethoxy)phenyl]prop-2-enamide were the most active in series **2** and in addition to activity against *S. aureus* and MRSA were highly active against *Enterococcus faecalis* and vancomycin-resistant *E. faecalis* isolates and against fast-growing *Mycobacterium smegmatis* and against slow-growing *M. marinum*, *M. tuberculosis* non-hazardous test models. In addition, the last three compounds of the above-mentioned showed insignificant cytotoxicity to primary porcine monocyte-derived macrophages.

## 1. Introduction

One of the basic strategies in finding new bioactive compounds is inspiration from natural substances [1,2,3]. This includes a variety of phenolic acid derivatives and analogs [4]. These acids, including cinnamic acid, are vital components of plants that are often found in fruits, vegetables, cereals, and legumes. They act in plants in a wide range of processes, such as pigmentation, growth, reproduction and resistance to pathogens or predators. Cinnamic acid is a naturally occurring aromatic acid with a long history of use as an additive in plant fragrances and spices. Its derivatives are important promising substances with great potential in the search for new pharmacologically active substances [5,6]. Cinnamic acid and its derivatives have attracted the attention of scientists in recent decades due to low toxicity and a wide range of biological activities such as antibacterial, antiviral, anti-inflammatory, cytotoxic, antidiabetic, hepatoprotective, antioxidant, neuroprotective, anxiolytic, and antituberculotic [7,8,9,10,11,12,13].

Various infections are an increasing worldwide threat. The growing drug resistance of bacterial strains is also a major danger. In addition, the development of cross-resistant and multidrug-resistant strains is a significant problem [14,15,16]. The most common resistant bacterial strains include methicillin-resistant *Staphylococcus aureus*, vancomycin-resistant *S. aureus*, vancomycin-resistant enterococci, penicillin-, and macrolides-resistant *Streptococcus pneumoniae*, cotrimoxazol-resistant *Escherichia coli*, the third generation of cephalosporin-resistant *E. coli* and *Klebsiella pneumoniae*, and carbapenem-resistant *E. coli*, *K. pneumoniae*, and *Pseudomonas aeruginosa* [16]. Tuberculosis caused by *Mycobacterium tuberculosis* is still one of the most lethal communicable diseases in the world. The spread of multidrug-, extensively drug- and totally drug-resistant tubercular strains is a great problem worldwide [17]. Therefore, it is important to approach these real threats responsibly and find new molecules with the potential to fight resistant microorganisms.

Recent studies investigating the antimicrobial activities of an extensive series of variously substituted cinnamic anilides have revealed compounds with promising anti-infective potential [10,12]. As the introduction of halogen into the molecule structure is known to increase antibacterial activity, e.g., [18,19,20,21,22] two series of anilides with presumed antibacterial activity from 4-chlorocinnamic and 3,4-dichlorocinnamic acids were designed. All newly synthesized compounds were evaluated for their activity against gram-positive bacteria and mycobacteria, and their cytotoxic profiles on cancer cell lines and the primary cells were studied in detail to identify a suitable candidate with optimal physicochemical and biological properties for further investigation, because in addition to the above-mentioned antimicrobial derivatives, various cinnamic acid derivatives are described as promising anticancer agents [7,8], e.g., derivatives of sinapic acid [23], caffeic acid [24], cinnamic acid-quinolone hybrids [25], oleanolic acid-cinnamic acid ester derivatives, glycyrrhetinic acid-cinnamic acid ester derivatives [26], and other variously substituted cinnamic acids [27].

## 2. Results and Discussion

### 2.1. Synthesis and Physicochemical Properties

**Scheme 1:**
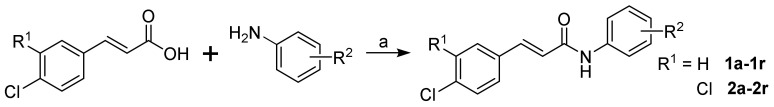
Synthesis of (2*E*)-3-(4-chlorophenyl)-*N*-arylprop-2-enanilides **1a**–**1r** and (2*E*)-3-(3,4-dichlorophenyl)-*N*-arylprop-2-enanilides **2a**–**2r**. *Reagents and conditions:* (**a**) PCl_3_, chlorobenzene, MW, 45 min [10,12].

All the studied 4-chlorocinnamanilides (series **1**) and 3,4-dichlorocinnamanilides (series **2**) were prepared according to Scheme 1 as described previously [10,12]. The condensation of (di)chlorocinnamic acid with appropriate substituted anilines using phosphorus trichloride in dry chlorobenzene under microwave conditions yielded a series of target mono- or dechlorinated *N*-aryl-(di)chlorocinnamamides **1a**–**1r** and **2a**–**2r**, see Table 1.

It can be stated that the pharmacokinetic (absorption, distribution, metabolism, elimination; ADME) and pharmacodynamic properties of bioactive agents are most significantly affected by lipophilicity, which is influenced by the chemical constitution and structure of individual agents. In addition to the mentioned ADME profile, it is responsible for interactions with biological targets. Lipophilicity is recognized as one of the most important parameters influencing ADME and bioactivity [28,29] and is also part, e.g., of Lipinski’s Rule of Five (Ro5) [30]. This thermodynamic parameter describing the molecule’s affinity for an aqueous or relatively lipophilic environment can be characterized by the logarithm of the partition coefficient (log *P*). In this study, lipophilicity was measured by reversed-phase high-performance liquid chromatography (RP-HPLC) under isocratic conditions with methanol as the organic modifier in the aqueous mobile phase and the logarithm of the capacity factor *k* was calculated, which is used as the lipophilicity index converted to the log *P* scale [28,31]. In addition to the log *k* values, the logarithm of the distribution coefficient *D*_pH_ at pH 6.5 and 7.4 was determined to clarify the behavior of the compounds under physiological conditions and possible ionization [28,29]. Instead of water, a buffer of suitable pH was used as part of the mobile phase. In addition to the above-mentioned experimental methods, commercially available ChemBioDraw Ultra 13.0 and ACD/Percepta ver. 2012 programs were used to calculate the lipophilicity values of all prepared compounds. All the results are shown in Table 1.

**Figure 1:**
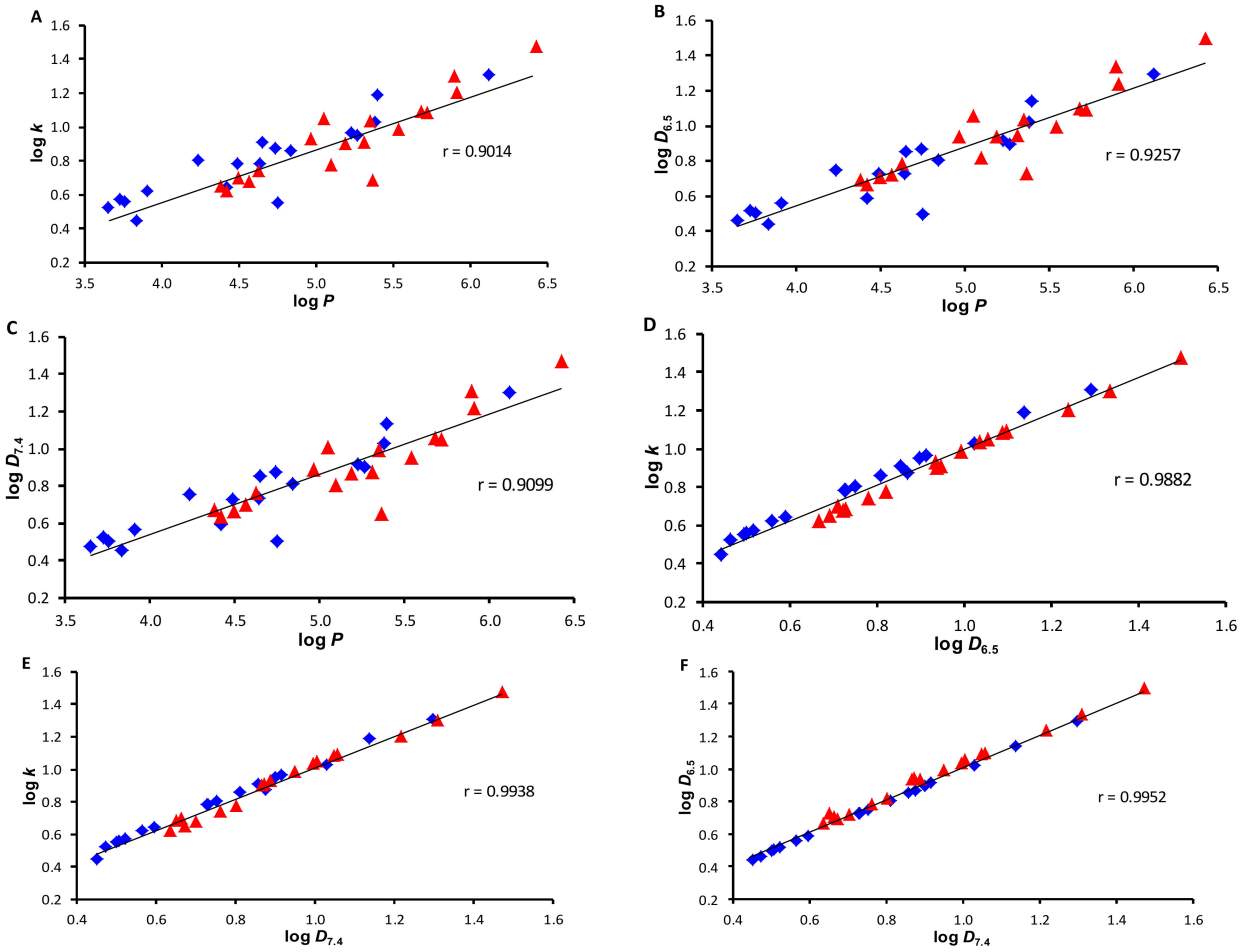
Comparison of predicted log *P* (ACD/Percepta) values with experimentally determined values of log *k* (**A**), log *D*_6.5_ (**B**), log *D*_7.4_ (**C**), and mutual correlation (**D**–**F**) of all prepared compounds **1a**–**2r**. (blue rhombuses = series **1**, red triangles = series **2**).

The log *P* values predicted by ChemDraw were identical for all three positional isomers. The Clog *P* values also calculated by ChemDraw were identical for at least two positional isomers, so this software is not applicable to these studied derivatives, unlike ADC/Percepta, which provided unique log *P* values for each derivative/isomer. The similarities/differences between the predicted and calculated values are shown in the graphs in Figure 1. It is logical and evident that the lipophilicity of the compounds differs within both series; in this case, by about 0.2. It can be seen from all the graphs that the matches between the predicted log *P* and the experimentally determined log *k* and log *D*, respectively, are very good with a correlation coefficient *r* in the range of 0.90–0.99 (*n* = 36).

Most substituents contain fluorine (either as a single atom or CF_3_ or OCF_3_ moieties). It is known that fluorine substituents easily form various inter- and intramolecular interactions, which can significantly affect the resulting behavior of the compounds in an aqueous environment [32,33,34,35]. If all fluorine-free derivatives were eliminated from the graphs, and the effects of the fluorine-based substituents in both series were evaluated separately, a slightly stronger agreement would be found in series **1** (average *r* = 0.91, *n* = 11) than in series **2** (average *r* = 0.89, *n* = 11) among fluoro-based substituents for all dependences of experimental log *k* and log *D* values on predicted lipophilicity log *P*. In the graphs of Figure 1A–C, significantly deflected points below the trend line can be observed for compounds **1h** and **2h** (R = 2-CF_3_). If these compounds are removed from the dependences, an average correlation coefficient *r* > 0.97 (*n* = 10) is obtained. After the elimination of the derivatives with these *ortho*-substituted CF_3_ moieties, a slight deviation can be observed for compound **2k** (R = 4-OCF_3_), after the removal of which *r* increases from 0.97 to 0.99 (*n* = 9). Within series **1**, the elimination of **1k** will increase *r* in the order of ten-thousandths. Thus, based on all these facts, it can be stated that within the monochlorinated series **1**, mutual interactions caused by the presence of fluoro-based substituents are much more significant.

### 2.2. In Vitro Antimicrobial Activity

The investigated compounds were screened for in vitro antibacterial activity against the reference and quality control strains *Staphylococcus aureus* ATCC 29213 and *Enterococcus faecalis* ATCC 29212 and representatives of multidrug-resistant bacteria and clinical isolates of methicillin-resistant *S. aureus* (MRSA) 63718, SA 630, SA 3202 [36] that were obtained from the National Institute of Public Health (Prague, Czech Republic), and three isolates from American crows of vanA-carrying vancomycin-resistant *E. faecalis* (VRE) 342B, 368, and 725B [37]. In addition, all the compounds were evaluated in vitro against *Mycobacterium smegmatis* ATCC 700084 and *M. marinum* CAMP 5644 as a safe alternative to *M. tuberculosis*. The genus *Mycobacterium* is a closely related group of fast and slow-growing species. *M. tuberculosis* causes one of the most serious human infections, tuberculosis [17]. Alternative model pathogens for *M. tuberculosis* can be used in laboratory studies to reduce risks and facilitate laboratory handling. *M. smegmatis* is an ideal representative of a fast-growing non-pathogenic microorganism particularly useful in the study of basic cellular processes of particular importance for pathogenic mycobacteria [38]. *M. marinum* is very closely related to *M. tuberculosis* and is the cause of TB-like infections in poikilothermic organisms, especially frogs and fish. *M. marinum* is a good model for study mainly due to the lower risk for laboratory workers, genetic relatedness and similar pathology as for human TB [39]. Activities are expressed as the minimum inhibitory concentrations (MICs). Surprisingly, the screening showed antimicrobial activity for only a few compounds, so only derivatives with antimicrobial activity are listed in Table 2. All other non-listed compounds had MICs of 256 µg/mL and greater.

Of the prepared 36 compounds, only three compounds from series **1** and 4 from series **2** were antibacterial-effective. The compounds of series **1** were completely inactive against *E. faecalis* and VRE and against *M. marinum*. On the other hand, when the compounds showed activity, the efficacy was comparable to or better than that of clinically used drugs. Compounds **1i** (R^2^ = 3-CF_3_), **1p** (R^2^ = 3,5-Cl_3_) and **1q** (R^2^ = 3,5-CF_3_) showed activity within the monochlorinated series **1**. In dichlorinated series **2**, compounds **2i** (R^2^ = 3-CF_3_), **2j** (R^2^ = 4-CF_3_), **2k** (R^2^ = 4-OCF_3_) and **2q** (R^2^ = 3,5-CF_3_) were active. The isolated activity of the unsubstituted (R^2^ = H) derivative **2a** against *M. smegmatis* was unexpected based on previous experience with analogues [40,41,42]. As MICs against MRSA isolates were, in fact, comparable with the MIC values observed against methicillin-susceptible *S. aureus* ATCC 29213, it could be assumed that the presence of *mecA* gene [43] did not affect the activity of these compounds. Thus, it can be speculated concerning the specific activity against *Staphylococcus* sp. Similarly, the close activity of the compounds against both VRE and *E. faecalis* indicates a mechanism of action unrelated to vancomycin resistance [37].

### 2.3. In Vitro Cell Viability

The effect of the compounds on viability of eukaryotic cells was evaluated on human leukemia cell line THP-1, human synovial cell line SW982, and isolated primary porcine monocytes-delivered macrophages (MDM). The general cytotoxic effect was studied on THP-1 cells in the serum-free medium (only THP-1 as the only cells used in this study are able to survive in serum-free medium). Most of the compounds had no cytotoxic effect on THP-1 cells. However, **1i**, **1p**, **1q**, **2a**, **2i**, **2j**, **2k**, and **2q** decreased cell viability with IC_50_ concentrations ranging from 0.5–7.5 µM (Table 2). On the other hand, the results showed that no cytotoxic effects were observed in the culture medium containing 10% FBS on any of the cell lines used to test the cytotoxicity of the compounds except derivative **2q**, which had a mild cytotoxic effect on MDM (IC_50_ 5–10 µM). This could be caused by the direct interaction of such compounds with serum proteins or by the effect of mitogens, cytokines, and hormones present in the serum. Obviously, the full medium is closer to physiological conditions in living organisms, so it can be assumed that these compounds could be non-toxic there.

**Figure 2:**
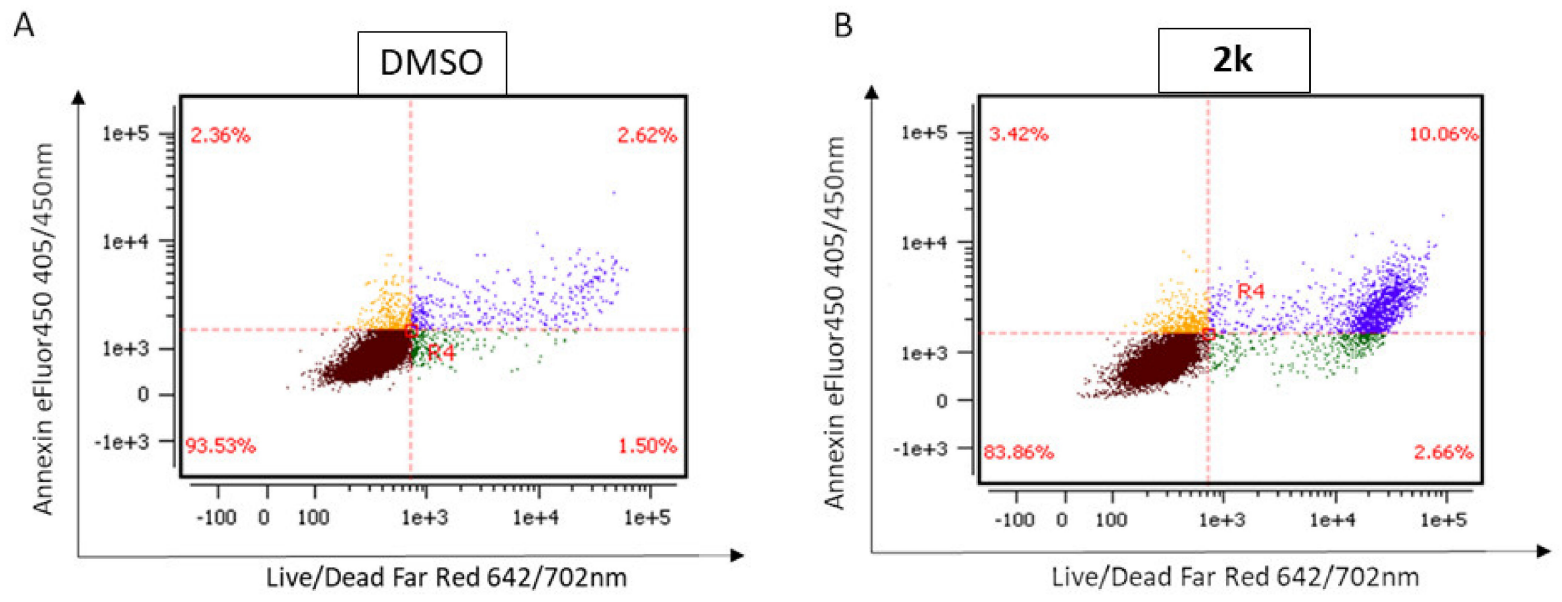

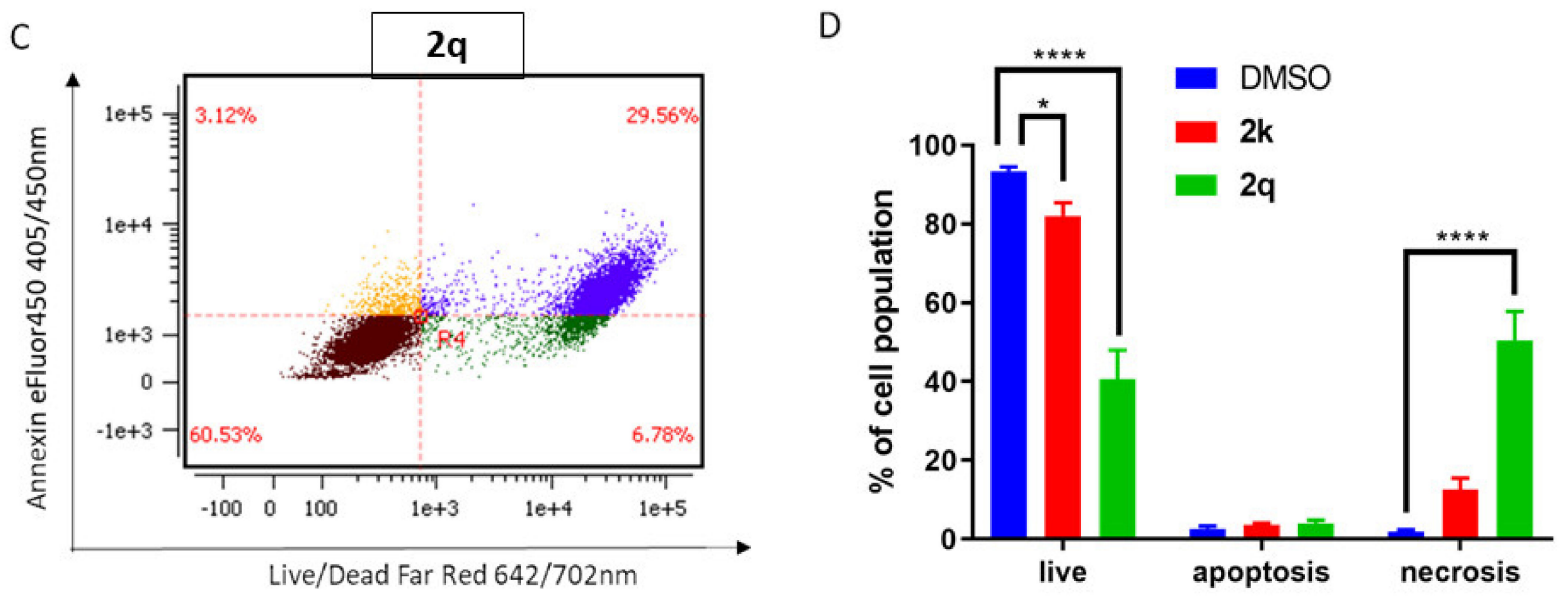
Cell death assay. Cells were treated by DMSO (**A**), **2q** (0.5 µM) (**B**), and **2k** (1.0 µM) (**C**) for 24 h. Representative dot plots of flow cytometry analysis after double staining of THP-1 cells by Annexin eFluor 450 and Live/Dead Far Red. Live cells (**Lower Left**), apoptosis (**Upper Left**) and necrosis (**Up Right**) were distinguished after. Average distribution of cell populations from three independent experiments-live, apoptotic and necrotic cells together with standard deviations, SE (**D**). * indicates statistical significance (*p* < 0.05) between indicated groups, **** indicates statistical significance (*p* < 0.0001) between indicated groups.

Subsequently, compounds **2k** and **2q** were selected for analysis by flow cytometry and cytotoxic effects were detected after treatment of cells by **2k** and **2q** in serum-free medium at two selected concentrations, 1.0 µM and 0.5 µM, respectively. Apoptosis was not observed but necrosis was significantly induced after **2q** treatment. Moreover, **2k** increased the number of dead cells (20%) (Figure 2). CCK-8 assay, which is based on cell viability detection, showed a 50% reduction. Thus, it can be assumed that **2k** attenuates cell metabolism.

### 2.4. Structure–Activity Relationships

As mentioned above, Ro5 is one of the accepted recommendations concerning the physicochemical parameters of biologically active agents. Ro5 contains the limits of specific molecular descriptors (Table 3) determined on the basis of experimentally and statistically obtained results such that a compound meeting this recommendation is druglike. However, a good druglike score does not make a molecule a drug and vice versa [44]. It is clear that bio-friendly properties are important in the context of specific interactions; therefore, Table 3 shows the profile of mainly Ro5 parameters characterizing the set of active compounds. In addition, based on previous experience, when we were convinced of the importance of electronic σ properties of substituents, i.e., the ability to affect the electron density of a compound, which results in the strength of interactions with biological targets [10,11,12,34,45,46], Table 3 shows the predicted electronic σ parameters of the whole substituted anilide ring characterizing the ability to withdraw or donate electrons to molecule system. For the same reason, i.e., the influence of the biological properties by the surface tension (ST) of the compounds [36,47], the calculated values of this parameter are given in Table 3.

Based on the data presented in Table 3, it can be stated that in general, the investigated compounds meet the Ro5 requirements. However, it should be mentioned that compound **2q** has the highest molecular weight within the effective agents (but in the limit) and all compounds except derivatives **1i** and **2a** possess slightly higher lipophilicity than recommended by Ro5. Thus, it can be stated that individual anilide fragments are characterized by electron-withdrawing properties and relatively high lipophilicity.

As approximately one-fifth of the prepared compounds demonstrated antimicrobial activity, a comprehensive structure–activity relationships (SAR) study cannot be performed. Based on the comparison with compounds unsubstituted on the cinnamic acid skeleton, it can be stated that the introduction of a chlorine atom in position C_(4)_ significantly increased the antimicrobial activity. The introduction of the second chlorine at position C_(3)_ resulted in a further significant extension and increase of activity against *E. faecalis* and VRE, which was not observed in the previous study. In previous studies, the activity of non-chlorinated *N*-cinnamanilides was found only for 3-CF_3_ and 3,5-CF_3_ substitutions against *S. aureus*, MRSA and *M. tuberculosis*, and only antistaphylococcal activity was determined in the case of 3-F-4-CF_3_ substituted derivative [10,12].

The activity of the 3-CF_3_ and 3,5-CF_3_ substituted derivatives (compounds **1i**, **2i**, **1q**, **2q**) could be expected [10]. The activity of 3,5-Cl substituted compound in series **1** and the activity of 4-CF_3_ and 4-OCF_3_ substituted derivatives in series **2** are gratifying. On the other hand, the total loss of any effect of derivatives **1r**, **2r** (R^2^ = 2-Br-4-OCF_3_) is surprising. In most cases, these are mono-/di-substitutions at the *meta* positions of the anilide ring by lipophilic electron-withdrawing substituents capable of forming hydrogen bonds.

**Figure 3:**
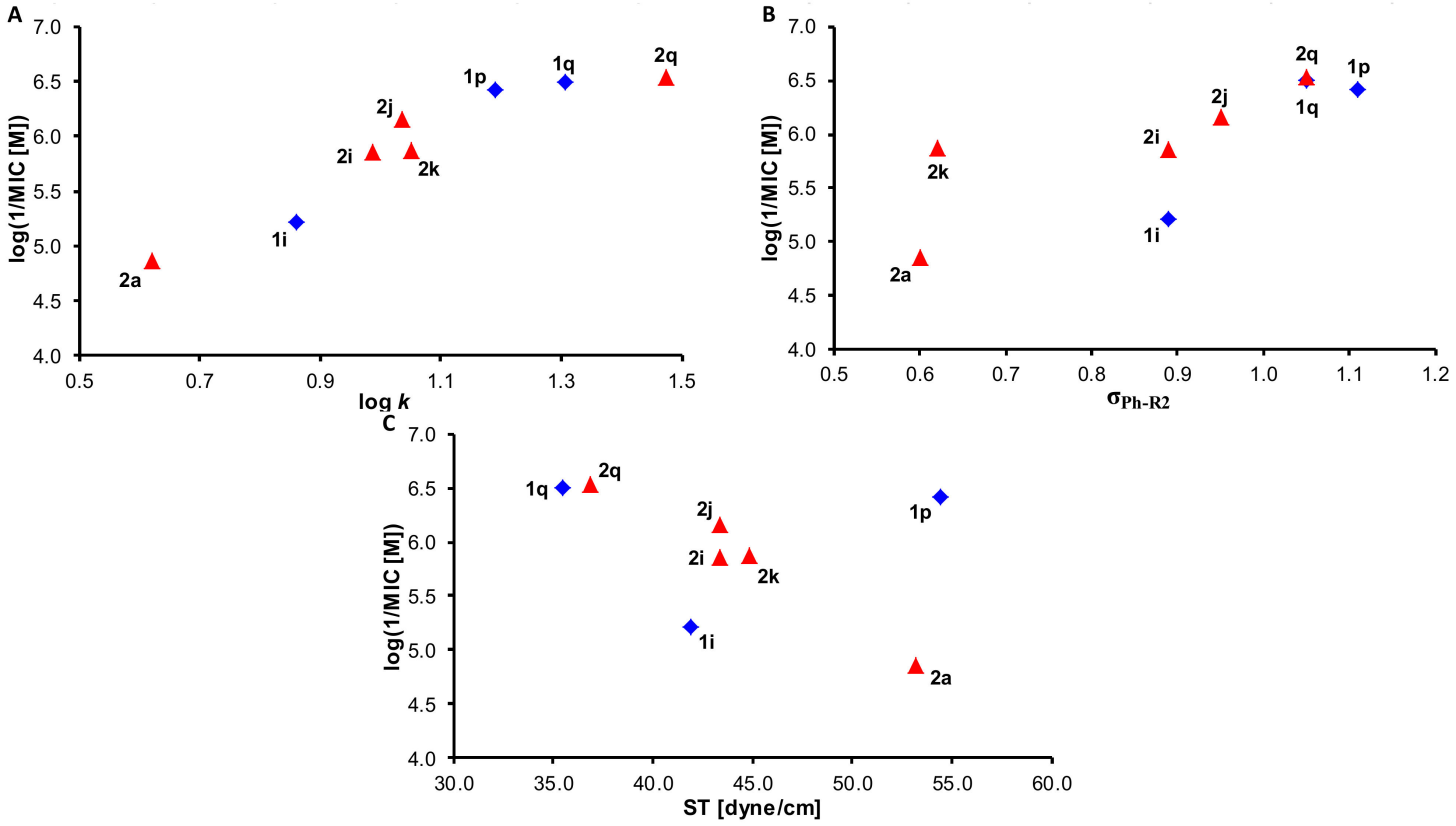
Relationships between in vitro activity against *S. aureus* ATCC 29,213 log(1/MIC [M]) and lipophilicity expressed as log *k* (**A**), electronic σ parameters of ring-substituted anilide ring (**B**) and and surface tension (**C**) of studied compounds. (blue rhombuses = series **1**, red triangles = series **2**).

However, graphs were created to better understand the relationships. The dependence of the activity against *S. aureus*, expressed as log(1/MIC [M]), on lipophilicity (log *k*) is illustrated in the graph in Figure 3a. (All MIC and IC_50_ values in the form of log(1/MIC [M]) and log(1/IC_50_ [M]) are listed in Table S1 in Supplementary Materials.) Increasing activity can be observed with increasing log *k* value. For the most effective compounds (**1p**, **1q** and **2q**), the effect of lipophilicity becomes less pronounced and the curve becomes a certain plateau. The influence of electronic effects on activity against *S. aureus* is shown in Figure 3b. There is a noticeable trend of increasing activity with increasing electron-withdrawing properties of the anilide part of the molecule.

The deviated position in Figure 3b for **2k** (R^2^ = 4-OCF_3_) is probably caused by the specific characteristics of this moiety, e.g., [48,49,50,51], are therein similar to the unpredictable behavior of methoxy-substituted compounds observed in other studies, e.g., [45,52,53,54,55]. The last Figure 3c shows the dependence of the surface tension on the activity. The antistaphylococcal activity within compounds of series **2** decreases with increasing surface tension, i.e., with decreasing ability of the compounds to act as surfactants. A similar course of trends between structure and activities of active compounds of both series can be traced for other antimicrobial activities against *E. faecalis*, MRSA, VRE, *M. smegmatis* and *M. marinum*, and are therefore are not listed.

**Figure 4:**
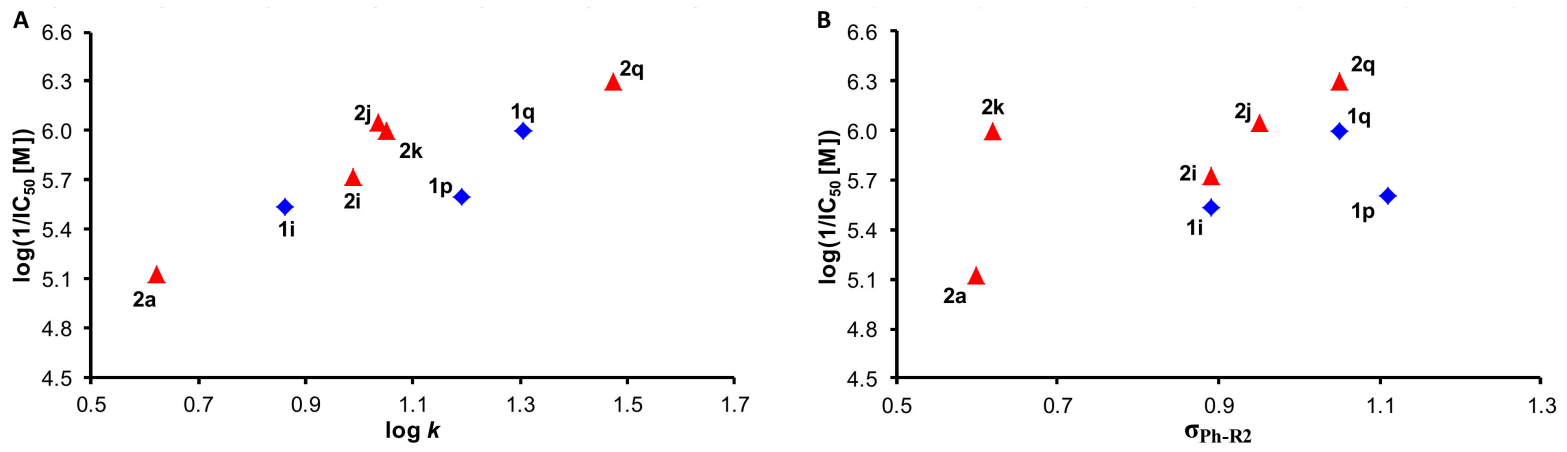

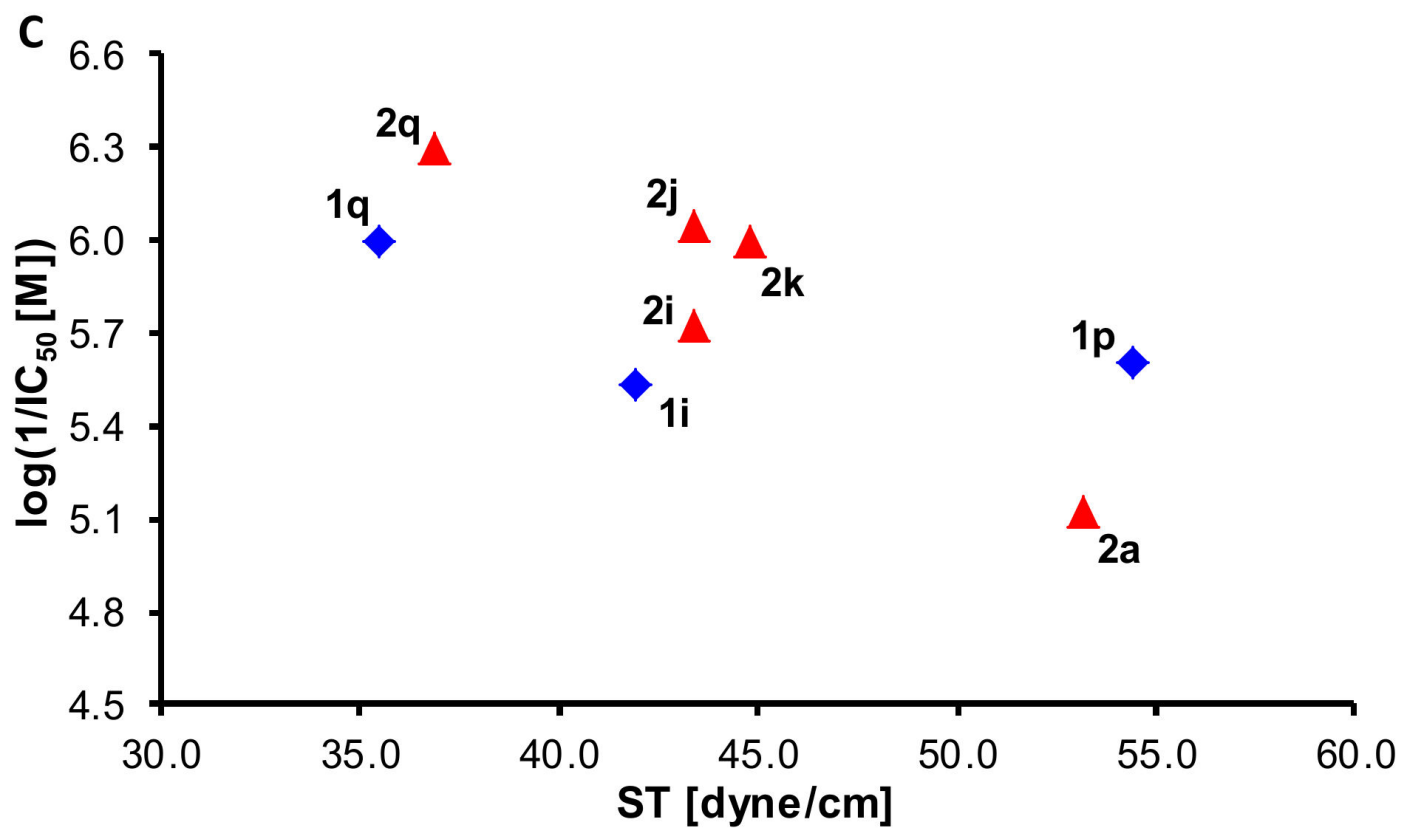
Relationships between in vitro cell viability on human monocytic leukemia cells (THP-1) in serum-free medium log(1/IC_50_ [M]) and lipophilicity expressed as log *k* (**A**), electronic σ parameters of ring-substituted anilide ring (**B**) and surface tension (**C**) of studied compounds (blue rhombuses = series **1**, red triangles = series **2**).

The relationships between the cell viability, expressed as log(1/IC_50_ [M]), on THP-1 cells in the serum-free medium on the physicochemical properties are illustrated in the graphs in Figure 4. The toxic effect increases with increasing lipophilicity, electron-withdrawing properties (within series **2**) and increasing surface activity (ability to reduce the surface tension of series **2** compounds). This observation supports the above-presented theory that the observed cytotoxic effect is mainly caused by cell necrosis, see Figure 2.

## 3. Materials and Methods

### 3.1. General Methods

All reagents were purchased from Merck (Sigma-Aldrich, St. Louis, MO, USA) and Alfa (Alfa-Aesar, Ward Hill, MA, USA). Reactions were performed using an Anton-Paar Monowave 50 microwave reactor (Graz, Austria). The melting points were determined on a Kofler hot-plate apparatus HMK (Franz Kustner Nacht KG, Dresden, Germany) and are uncorrected. Infrared (IR) spectra were recorded on Nicolet iS5 IR spectrometer (Thermo Scientific, West Palm Beach, FL, USA). The spectra were obtained by the accumulation of 256 scans with 2 cm^−1^ resolution in the region of 4000–450 cm^−1^. All ^1^H- and ^13^C-NMR spectra were recorded on a JEOL JNM-ECA 600II device (600 MHz for ^1^H and 150 MHz for ^13^C, JEOL, Tokyo, Japan) in dimethyl sulfoxide-*d_6_* (DMSO-*d_6_*). ^1^H and ^13^C chemical shifts (δ) are reported in ppm. High-resolution mass spectra were measured using a high-performance liquid chromatograph Dionex UltiMate^®^ 3000 (Thermo Scientific, West Palm Beach, FL, USA) coupled with an LTQ Orbitrap XL^TM^ Hybrid Ion Trap-Orbitrap Fourier Transform Mass Spectrometer (Thermo Scientific) equipped with a HESI II (heated electrospray ionization) source in the positive and negative mode.

### 3.2. Synthesis

#### General Procedure for Synthesis of Carboxamide Derivatives **1a**–**2r**

4-Chlorocinnamic acid/3,4-dichlorocinnamic acid (1.0 mM) was suspended in dry chlorobenzene (6.0 mL) at ambient temperature and phosphorus trichloride (0.5 mM, 0.5 eq.), and the corresponding substituted aniline (1.0 mM, 1 eq.) were added dropwise. The reaction mixture was transferred to the microwave reactor, where the synthesis was performed (40 min, 130 °C). Then the mixture was cooled to 40 °C, and then the solvent was removed to dryness under reduced pressure. The residue was washed with hydrochloride acid and water. The crude product was recrystallized from ethanol.

*(2E)-3-(4-Chlorophenyl)-N-phenylprop-2-enamide* (**1a**) [56]. Yield 63%; Mp 164–167 °C; IR (cm^−1^): 3275 (N-H), 1656 (C=O), 1624 (C=C), 1597 (C-C_arom_), ^1^H-NMR (DMSO-*d_6_*), δ: 10.23 (s, 1H), 7.70 (d, *J* = 7.6 Hz, 2H), 7.66–7.64 (m, 2H), 7.58 (d, *J* = 15.8 Hz, 1H), 7.52–7.50 (m, 2H), 7.35–7.32 (m, 2H), 7.08–7.06 (m, 1H), 6.84 (d, *J* = 15.8 Hz, 1H); ^13^C-NMR (DMSO-*d_6_*), δ: 163.3, 139.2, 138.8, 134.2, 133.7, 129.4, 129.1, 128.8, 123.43, 123.1, 119.2 (Figure S1); HR-MS: for C_15_H_11_ONCl [M-H]^−^ calculated 256.0535 *m*/*z*, found 256.0530 *m*/*z*.

*(2E)-3-(4-Chlorophenyl)-N-(2-fluorophenyl)prop-2-enamide* (**1b**). Yield 64%; Mp 186–189 °C; IR (cm^−1^): 3295 (N-H), 1658 (C=O), 1625 (C=C), 1596 (C-C_arom_); ^1^H NMR (DMSO-*d_6_*), δ: 9.96 (s, 1H), 8.11 (t, *J* = 7.6 Hz, 1H), 7.66–7.64 (m, 2H), 7.59 (d, *J* = 15.8 Hz, 1H), 7.52–7.51 (m, 2H), 7.30–7.26 (m, 1H), 7.20–7.14 (m, 2H), 7.08 (d, *J* = 15.8 Hz, 1H); ^13^C-NMR (DMSO-*d_6_*), δ: 163.7, 153.3 (d, *J* = 245.7 Hz), 139.3, 134.3, 133.7, 129.5, 129.1, 126.3 (d, *J* = 11.6 Hz), 125.1 (d, *J* = 8.7 Hz), 124.4 (d, *J* = 2.9 Hz), 123.6, 122.7, 115.5 (d, *J* = 20.2 Hz) (Figure S2); HR-MS: for C_15_H_10_ONClF [M-H]^−^ calculated 274.0440 *m*/*z*, found 274.0435 *m*/*z*.

*(2E)-3-(4-Chlorophenyl)-N-(3-fluorophenyl)prop-2-enamide* (**1c**). Yield 72%; Mp 194–197 °C; IR (cm^−1^): 3261 (N-H), 1658 (C=O), 1622 (C=C), 1605 (C-C_arom_); ^1^H-NMR (DMSO-*d_6_*), δ: 10.44 (s, 1H), 7.74–7.71 (m, 1H), 7.67–7.65 (m, 2H), 7.61 (d, *J* = 15.8 Hz, 1H), 7.52–7.50 (m, 2H), 7.38–7.36 (m, 2H), 6.92–6.88 (m, 1H), 6.81 (d, *J* = 15.8 Hz, 1H); ^13^C-NMR (DMSO-*d_6_*), δ: 163.6, 162.2 (d, *J* = 241.3 Hz), 140.9 (d, *J* = 10.1 Hz), 139.4, 134.4, 133.6, 130. 5 (d, *J* = 10.1 Hz), 129.5, 129.1, 122.7, 115.0, 109.9 (d, *J* = 21.7 Hz), 106.0 (d, *J* = 26.0 Hz) (Figure S3); HR-MS: for C_15_H_10_ONClF [M-H]^−^ calculated 274.0440 *m*/*z*, found 274.0434 *m*/*z*.

*(2E)-3-(4-Chlorophenyl)-N-(4-fluorophenyl)prop-2-enamide* (**1d**). Yield 59%; Mp 175–177 °C; IR (cm^−1^): 3265 (N-H), 1656 (C=O), 1623 (C=C), 1591 (C-C_arom_); ^1^H-NMR (DMSO-*d_6_*), δ: 10.29 (s, 1H), 7.72–7.70 (m, 2H), 7.66–7.64 (m, 2H), 7.58 (d, *J* = 15.8 Hz, 1H), 7.51–7.50 (m, 2H), 7.18 (t, *J* = 8.6 Hz, 2H), 6.80 (d, *J* = 15.8 Hz, 1H); ^13^C-NMR (DMSO-*d_6_*), δ: 163.2, 158.1 (d, *J* = 239.9 Hz), 138.9, 135.6, 134.2, 133.7, 129.4, 129.1, 122.9, 121.0 (d, *J* = 8.7 Hz), 115.4 (d, *J* = 21.7 Hz) (Figure S4); HR-MS: for C_15_H_10_ONClF [M-H]^−^ calculated 274.0440 *m*/*z*, found 274.0435 *m*/*z*.

*(2E)-N-(2-Chlorophenyl)-3-(4-chlorophenyl)prop-2-enamide* (**1e**). Yield 68%; Mp 195–196 °C; IR (cm^−1^): 3270 (N-H), 1654 (C=O), 1621 (C=C), 1585 (C-C_arom_); ^1^H-NMR (DMSO-*d_6_*), δ: 9.69 (s, 1H), 7.93 (d, *J* = 8.2 Hz, 1H), 7.68–7.67 (m, 2H), 7.60 (d, *J* = 15.8 Hz, 1H), 7.53–7.52 (m, 3H), 7.37–7.35 (m, 1H), 7.22–7.19 (m, 1H), 7.12 (d, *J* = 15.8 Hz, 1H); ^13^C-NMR (DMSO-*d_6_*), δ: 163.7, 139.4, 134.9, 134.3, 133.7, 129.5, 129.1, 127.5, 126.1, 125.8, 125.5, 122.6 (Figure S5); HR-MS: for C_15_H_10_ONCl_2_ [M-H]^−^ calculated 290.0145 *m*/*z*, found 290.0140 *m*/*z*.

*(2E)-N-(3-Chlorophenyl)-3-(4-chlorophenyl)prop-2-enamide* (**1f**) [57]. Yield 63%; Mp 177–178 °C; IR (cm^−1^): 3291 (N-H), 1660 (C=O), 1620 (C=C), 1592 (C-C_arom_); ^1^H-NMR (DMSO-*d_6_*) δ: 10.41 (s, 1H), 7.94 (t, *J* = 2.1 Hz, 1H), 7.66–7.65 (m, 2H), 7.60 (d, *J* = 15.8 Hz, 1H), 7.53–7.49 (m, 3H), 7.36 (t, *J* = 7.9 Hz, 1H), 7.13 (dd, *J* = 6.9, 2.1 Hz, 1H), 6.80 (d, *J* = 15.8 Hz, 1H); ^13^C-NMR (DMSO-*d_6_*), δ: 163.7, 140.6, 139.4, 134.4, 133.5, 133.2, 130.5, 129.5, 129.1, 123.2, 122.6, 118.72, 117.7(Figure S6); HR-MS: for C_15_H_10_ONCl_2_ [M-H]^−^ calculated 290.0145 *m*/*z*, found 290.0143 *m*/*z*.

*(2E)-N,3-bis(4-Chlorophenyl)prop-2-enamide* (**1g**) [58]. Yield 71%; Mp 211–213 °C; IR (cm^−1^): 3245 (N-H), 1660 (C=O), 1623 (C=C), 1591 (C-C_arom_); ^1^H-NMR (DMSO-*d_6_*), δ: 10.36 (s, 1H), 7.73–7.72 (m, 2H), 7.66–7.64 (m, 2H), 7.59 (d, *J* = 15.8 Hz, 1H), 7.51–7.50 (m, 2H), 7.39–7.37 (m, 2H), 6.81 (d, *J* = 15.8 Hz, 1H); ^13^C-NMR (DMSO-*d_6_*), δ: 163.4, 139.1, 138.1, 134.3, 133.6, 129.5, 129.1, 128.7, 127.0, 122.8, 120.8 (Figure S7); HR-MS: for C_15_H_10_ONCl_2_ [M-H]^−^ calculated 290.0145 *m*/*z*, found 290.0143 *m*/*z*.

*(2E)-3-(4-Chlorophenyl)-N-[2-(trifluoromethyl)phenyl]prop-2-enamide* (**1h**). Yield 66%; Mp 183–186 °C; IR (cm^−1^): 3265 (N-H), 1654 (C=O), 1621 (C=C), 1591 (C-C_arom_); ^1^H-NMR (DMSO-*d_6_*), δ: 9.75 (s, 1H), 7.76 (d, *J* = 8.2 Hz, 1H), 7.71 (t, *J* = 7.6 Hz, 1H), 7.68–7.66 (m, 2H), 7.63 (d, *J* = 8.2 Hz, 1H), 7.59 (d, *J* = 15.8 Hz, 1H), 7.52–7.51 (m, 2H), 7.47 (t, *J* = 7.6 Hz, 1H), 7.01 (d, *J* = 15.8 Hz, 1H); ^13^C-NMR (DMSO-*d_6_*), δ: 164.4, 139.4, 135.3, 134.3, 133.6, 133.0, 129.7, 129.5, 129.1, 126.6, 126.3 (q, *J* = 4.3 Hz), 124.3 (q, *J* = 28.9 Hz), 123.6 (q, *J* = 273.1 Hz), 122.2 (Figure S8); HR-MS: for C_16_H_10_ONClF_3_ [M-H]^−^ calculated 324.0409 *m*/*z*, found 324.0400 *m*/*z*.

*(2E)-3-(4-Chlorophenyl)-N-[3-(trifluoromethyl)phenyl]prop-2-enamide* (**1i**). Yield 59%; Mp 168–169 °C; IR (cm^−1^): 3267 (N-H), 1662 (C=O), 1627 (ν C=O), 1557 (C-C_arom_); ^1^H-NMR (DMSO-*d_6_*), δ: 10.57 (s, 1H), 8.20 (s, 1H), 7.86 (d, *J* = 8.2 Hz, 1H), 7.68–7.66 (m, 2H), 7.62 (d, *J* = 15.8 Hz, 1H), 7.58 (t, *J* = 7.9 Hz, 1H), 7.53–7.51 (m, 2H), 7.43 (d, *J* = 7.6 Hz, 1H), 6.81 (d, *J* = 15.8 Hz, 1H); ^13^C-NMR (DMSO-*d_6_*), δ: 163.8, 139.9, 139.6, 134.4, 133.5 130.1, 129.5, 129.5 (q, *J* = 31.8 Hz), 129.1, 124.1 (q, *J* = 271.7 Hz), 122.8, 122.5, 119.7 (q, *J* = 4.3 Hz), 115.3 (q, *J* = 4.3 Hz) (Figure S9); HR-MS: for C_16_H_10_ONClF_3_ [M-H]^−^ calculated 324.0409 *m*/*z*, found 324.0400 *m*/*z*.

*(2E)-3-(4-Chlorophenyl)-N-[4-(trifluoromethyl)phenyl]prop-2-enamide* (**1j**). Yield 65%; Mp 196–199 °C; IR (cm^−1^): 3282 (N-H), 1655(C=O), 1619 (C=C), 1599 (C-C_arom_), 1530 (δ NH); ^1^H-NMR (DMSO-*d_6_*), δ: 10.59 (s, 1H), 7.90 (d, *J* = 8.9 Hz, 2H), 7.71 (d, *J* = 8.9 Hz, 2H), 7.68–7.67 (m, 2H), 7.63 (d, *J* = 15.8 Hz, 1H), 7.53–7.51 (m, 2H), 6.84 (d, *J* = 15.8 Hz, 1H); ^13^C-NMR (DMSO-*d_6_*), δ: 163.9, 142.8, 139.7, 134.5, 133.5, 129.6, 129.1, 126.2 (q, *J* = 4.3 Hz), 124.4 (q, *J* = 271.7 Hz), 123.4 (q, *J* = 31.8 Hz), 122.6, 119.2 (Figure S10); HR-MS: for C_16_H_10_ONClF_3_ [M-H]^−^ calculated 324.0409 *m*/*z*, found 324.0400 *m*/*z*.

*(2E)-3-(4-Chlorophenyl)-N-[4-(trifluoromethoxy)phenyl]prop-2-enamide* (**1k**). Yield 56%; Mp 161–163 °C; IR (cm^−1^): 3266 (N-H), 1654 (C=O), 1621 (C=C), 1567 (C-C_arom_); ^1^H-NMR (DMSO-*d_6_*), δ: 10.43 (s, 1H), 7.81–7.79 (m, 2H), 7.67–7.65 (m, 2H), 7.61 (d, *J* = 15.8 Hz, 1H), 7.52–7.50 (m, 2H), 7.35–7.34 (m, 2H), 6.82 (d, *J* = 15.8 Hz, 1H); ^13^C-NMR (DMSO-*d_6_*), δ: 163.5, 143.7, 139.3, 138.4, 134.3, 133.6, 129.5, 129.1, 122.7, 121.7, 121.6, 120.2 (q, *J* = 255.3 Hz) (Figure S11); HR-MS: for C_16_H_10_O_2_NClF_3_ [M-H]^−^ calculated 340.0358 *m*/*z*, found 340.0346 *m*/*z*.

*(2E)-3-(4-Chlorophenyl)-N-(2,4-difluorophenyl)prop-2-enamide* (**1l**). Yield 60%; Mp 194–196 °C; IR (cm^−1^): 3276 (N-H), 1656 (C=O), 1621 (C=C), 1568 (C-C_arom_); ^1^H-NMR (DMSO-*d_6_*), δ: 9.97 (s, 1H), 8.06–8.02 (m, 1H), 7.65–7.63 (m, 2H), 7.59 (d, *J* = 15.8 Hz, 1H), 7.52–7.49 (m, 2H), 7.33 (ddd, *J* = 11.2, 8.8, 2.7 Hz, 1H), 7.11–7.07 (m, 1H), 7.02 (d, *J* = 15.8 Hz, 1H); ^13^C-NMR (DMSO-*d_6_*), δ: 163.7, 158.4 (dd, *J* = 242.8, 11.6 Hz), 153.4 (dd, *J* = 248.5, 13.0 Hz), 139.4, 134.3, 133.6, 129.5, 129.1, 125.0 (dd, *J* = 8.7, 2.9 Hz), 122.9 (dd, *J* = 11.6, 4.3 Hz), 122.4, 111.2 (dd, *J* = 21.7, 2.9 Hz), 104.2 (multiplet) (Figure S12); HR-MS: for C_15_H_9_ONClF_2_ [M-H]^−^ calculated 292.0346 *m*/*z*, found 292.0338 *m*/*z*.

*(2E)-3-(4-Chlorophenyl)-N-(3,5-difluorophenyl)prop-2-enamide* (**1m**). Yield 61%; Mp 224–226 °C; IR (cm^−1^): 3268 (N-H), 1662 (C=O), 1615 (C=C), 1558 (C-C_arom_); ^1^H-NMR (DMSO-*d_6_*), δ: 10.60 (s, 1H), 7.68–7.66 (m, 2H), 7.63 (d, *J* = 15.8 Hz, 1H), 7.53–7.50 (m, 2H), 7.41 (dd, *J* = 9.6, 2.1 Hz, 2H), 6.93 (tt, *J* = 9.4, 2.3 Hz, 1H), 6.77 (d, *J* = 15.8 Hz, 1H); ^13^C-NMR (DMSO-*d_6_*), δ: 163.9, 162.5 (dd, *J* = 242.8, 14.5 Hz), 141.6 (t, *J* = 13.0 Hz), 139.9, 134.5, 133.4, 129.6, 129.1, 122.2, 102.1 (dd, *J* = 23.1, 5.8 Hz), 98.6 (t, *J* = 26.0 Hz) (Figure S13); HR-MS: for C_15_H_9_ONClF_2_ [M-H]^−^calculated 292.0346 *m*/*z*, found 292.0342 *m*/*z*.

*(2E)-3-(4-Chlorophenyl)-N-(2,4-dichlorophenyl)prop-2-enamide* (**1n**). Yield 59%; Mp 200–202 °C; IR (cm^−1^): 3275 (N-H), 1656 (C=O), 1622 (C=C), 1582 (C-C_arom_); ^1^H-NMR (DMSO-*d_6_*), δ: 9.76 (s, 1H), 7.98 (d, *J* = 8.9 Hz, 1H), 7.68–7.65 (m, 3H), 7.61 (d, *J* = 15.8 Hz, 1H), 7.53–7.50 (m, 2H), 7.44 (dd, *J* = 8.9, 2.7 Hz, 1H), 7.12 (d, *J* = 15.8 Hz, 1H); ^13^C-NMR (DMSO-*d_6_*), δ: 163.8, 139.8, 134.4, 134.1, 133.6, 129.5, 129.1, 129.1, 128.9, 127.6, 126.5, 126.3, 122.4 (Figure S14); HR-MS: for C_15_H_9_ONCl_3_ [M-H]^−^ calculated 323.9755 *m*/*z*, found 323.9761 *m*/*z*.

*(2E)-3-(4-Chlorophenyl)-N-(2,5-dichlorophenyl)prop-2-enamide* (**1o**). Yield 74%; Mp 156–158 °C; IR (cm^−1^): 3412 (N-H), 1661 (C=O), 1630 (C=C), 1579 (C-C_arom_); ^1^H-NMR (DMSO-*d_6_*), δ: 9.77 (s, 1H), 8.13 (d, *J* = 2.1 Hz, 1H), 7.68–7.66 (m, 2H), 7.62, (d, *J* = 15.8 Hz, 1H), 7.56 (d, *J* = 8.2 Hz, 1H), 7.53–7.51 (m, 2H), 7.26 (dd, *J* = 8.6, 2.4 Hz, 1H), 7.17 (d, *J* = 15.8 Hz, 1H); ^13^C-NMR (DMSO-*d_6_*), δ: 164.0, 140.0, 136.2, 134.5, 133.6, 131.6, 130.9, 129.6, 129.1, 125.5, 124.1, 123.7, 122.3 (Figure S15); HR-MS: for C_15_H_9_ONCl_3_ [M-H]^−^ calculated 323.9755 *m*/*z*, found 323.9757 *m*/*z*.

*(2E)-3-(4-Chlorophenyl)-N-(3,5-dichlorophenyl)prop-2-enamide* (**1p**). Yield 67%; Mp 214–217 °C; IR (cm^−1^): 3291 (N-H), 1660 (C=O), 1618 (C=C), 1582 (C-C_arom_); ^1^H-NMR (DMSO-*d_6_*), δ: 10.56 (s, 1H), 7.75 (d, *J* = 2.1 Hz, 2H), 7.68–7.66 (m, 2H), 7.62 (d, *J* = 15.8 Hz, 1H), 7.52–7.50 (m, 2H), 7.29 (t, *J* = 2.1 Hz, 1H), 6.75 (d, *J* = 15.8 Hz, 1H); ^13^C-NMR (DMSO-*d_6_*), δ: 163.9, 141.5, 140.0, 134.6, 134.1, 133.4, 129.6, 129.1, 122.6, 122.2, 117.3 (Figure S16); HR-MS: for C_15_H_9_ONCl_3_ [M-H]^−^ calculated 323.9755 *m*/*z*, found 323.9758 *m*/*z*.

*(2E)-N-[3,5-bis(Trifluoromethyl)phenyl]-3-(4-chlorophenyl)prop-2-enamide* (**1q**). Yield 71%; Mp 178–181 °C; IR (cm^−1^): 3282 (N-H Stretch), 1664 (C=O), 1632 (C=C), 1567 (C-C_arom_); ^1^H-NMR (DMSO-*d_6_*), δ: 10.88 (s, 1H), 8.34 (s, 2H), 7.75 (s, 1H), 7.68–7.66 (m, 2H), 7.65 (d, *J* = 15.8 Hz, 1H), 7.52–7.49 (m, 2H), 6.76 (d, *J* = 15.8 Hz, 1H); ^13^C-NMR (DMSO-*d_6_*), δ: 164.2, 141.0, 140.4, 134.7, 133.2, 130.8 (q, *J* = 33.2 Hz), 129.7, 129.1, 123.2 (q, *J* = 271.7 Hz), 121.9, 118.9 (q, *J* = 2.9 Hz), 116.1 (q, *J* = 4.3 Hz) (Figure S17); HR-MS: for C_17_H_9_ONClF_6_ [M-H]^−^ calculated 392.0282 *m*/*z*, found 392.0288 *m*/*z*.

*(2E)-N-[2-Bromo-4-(trifluoromethoxy)phenyl]-3-(4-chlorophenyl)prop-2-enamide* (**1r**). Yield 63%; Mp 144–146 °C; IR (cm^−1^): 3276 (N-H), 1659 (C=O), 1628 (C=C), 1587 (C-C_arom_); ^1^H-NMR (DMSO-*d_6_*), δ: 9.77 (s, 1H), 7.91 (d, *J* = 8.9 Hz, 1H), 7.79 (d, *J* = 2.7 Hz, 1H), 7.69–7.67 (m, 2H), 7.62 (d, *J* = 15.8 Hz, 1H), 7.54–7.51 (m, 2H), 7.47 (dd, *J* = 8.9, 2.7 Hz, 1H), 7.08 (d, *J* = 15.8 Hz, 1H); ^13^C-NMR (DMSO-*d_6_*), δ: 163.8, 145.1, 139.8, 135.8, 134.4, 133.6, 129.6, 129.1, 127.4, 125.6, 122.2, 120.9, 120.0 (q, *J* = 257.2 Hz), 117.5 (Figure S18); HR-MS: for C_16_H_11_O_2_NBrF_3_ [M+H]^+^ calculated 419.9608 *m*/*z*, found 419.9432 *m*/*z*.

*(2E)-3-(3,4-Dichlorophenyl)-N-phenylprop-2-enamide* (**2a**) [59]. Yield 63%; Mp 140–143 °C; IR (cm^−1^): 3244 (N-H), 1654 (C=O), 1616 (C=C), 1593 (C-C_arom_); ^1^H-NMR (DMSO-*d_6_*) δ: 10.23 (s, 1H), 7.90 (d, *J* = 2.1 Hz, 1H); 7.71–7.69 (m, 3H), 7.62 (dd, *J* = 8.9, 2.1 Hz, 1H); 7.57 (d, *J* = 15.8 Hz, 1H); 7.35–7.32 (m, 2H), 7.09–7.06 (m, 1H), 6.89 (d, *J* = 15.8 Hz, 1H); ^13^C-NMR (DMSO-*d_6_*), δ: 163.0, 139.1, 137.4, 135.7, 131.9, 131.7, 131.13, 129.6, 128.8, 127.4, 124.6, 123.5, 119.2 (Figure S19); HR-MS: for C_15_H_10_ONCl_2_ [M-H]^−^ calculated 290.0145 *m*/*z*, found 290.0146 *m*/*z*.

*(2E)-N-(2-Fluorophenyl)-3-(3,4-dichlorophenyl)prop-2-enamide* (**2b**). Yield 67%; Mp 151–153 °C; IR (cm^−1^): 3331 (N-H), 1662 (C=O), 1618 (C=C), 1553 (C-C_arom_); ^1^H-NMR (DMSO-*d_6_*), δ: 9.96 (s, 1H), 8.12 (t, *J* = 7.2 Hz, 1H), 7.90 (d, *J* = 2.1 Hz, 1H), 7.72 (d, *J* = 8.2 Hz, 1H), 7.62 (dd, *J* = 8.2, 2.1 Hz, 1H), 7.58 (d, *J* = 15.8 Hz, 1H), 7.28 (ddd, *J* = 11.3, 7.9, 2.1 Hz, 1H), 7.21–7.13 (m, 3H); ^13^C-NMR (DMSO-*d_6_*), δ: 163.4, 153.2 (d, *J* = 244.2 Hz), 138.0, 135.7, 132.0, 131.8, 131.2, 129.61, 127.5, 126.3 (d, *J* = 11.6 Hz), 125.2 (d, *J* = 7.2 Hz), 124.5 (d, *J* = 2.9 Hz), 124.2, 123.5, 115.5 (d, *J* = 18.8 Hz) (Figure S20); HR-MS: for C_15_H_9_ONCl_2_F [M-H]^−^ calculated 308.0051 *m*/*z*, found 308.0049 *m*/*z*.

*(2E)-N-(3-Fluorophenyl)-3-(3,4-dichlorophenyl)prop-2-enamide* (**2c**). Yield 71%; Mp 158–161 °C; IR (cm^−1^): 3431 (N-H), 1685 (C=O), 1618 (C=C), 1596 (C-C_arom_); ^1^H-NMR (DMSO-*d_6_*), δ: 10.45 (s, 1H), 7.91 (d, *J* = 2.1 Hz, 1H), 7.73–7.70 (m, 2H), 7.63 (dd, *J* = 8.2, 2.1 Hz, 1H), 7.59 (d, *J* = 15.8 Hz, 1H), 7.39–7.35 (m, 2H), 6.93–6.89 (m, 1H), 6.86 (d, *J* = 15.8 Hz, 1H); ^13^C-NMR (DMSO-*d_6_*), δ: 163.3, 162.2 (d, *J* = 241.3 Hz), 140.8 (d, *J* = 11.6 Hz), 138.0, 135.5, 132.1, 131.8, 131.2, 130.5 (d, *J* = 10.1 Hz), 129.7, 127.5, 124.2, 115.0, 110.0 (d, *J* = 21.7 Hz), 106.1 (d, *J* = 27.5 Hz) (Figure S21); HR-MS: for C_15_H_9_ONCl_2_F [M-H]^−^ calculated 308.0051 *m*/*z*, found 308.0049 *m*/*z*.

*(2E)-N-(4-Fluorophenyl)-3-(3,4-dichlorophenyl)prop-2-enamide* (**2d**). Yield 63%; Mp 142–145 °C; IR (cm^−1^):3278 (N-H), 1664 (C=O), 1626 (C=C), 1552 (C-C_arom_); ^1^H-NMR (DMSO-*d_6_*), δ: 10.31 (s, 1H), 7.90 (d, *J* = 2.1 Hz, 1H), 7.72–7.69 (m, 3H), 7.62 (dd, *J* = 8.2, 2.1 Hz, 1H), 7.57 (d, *J* = 15.8 Hz, 1H), 7.20–7.16 (m, 2H), 6.86 (d, *J* = 15.8 Hz, 1H); ^13^C-NMR (DMSO-*d_6_*), δ: 163.0, 158.1 (d, *J* = 239.9 Hz), 137.5, 135.6, 135.5 (d, *J* = 2.9 Hz), 131.9, 131.8, 131.1, 129.6, 127.4, 124.4, 121.0 (d, *J* = 7.2 Hz), 115.4 (d, *J* = 23.1 Hz) (Figure S22); HR-MS: for C_15_H_9_ONCl_2_F [M-H]^−^ calculated 308.0051 *m*/*z*, found 308.0049 *m*/*z*.

*(2E)-N-(2-Chlorophenyl)-3-(3,4-dichlorophenyl)prop-2-enamide* (**2e**). Yield 68%; Mp 154–156 °C; IR (cm^−1^): 3293 (N-H), 1659 (C=O), 1626 (C=C), 1592 (C-C_arom_); ^1^H-NMR (DMSO-*d_6_*), δ: 9.66 (s, 1H), 7.96 (d, *J* = 8.2 Hz, 1H), 7.93 (d, *J* = 1.4 Hz, 1H), 7.72–7.71 (m, 1H), 7.64 (dd, *J* = 8.2, 2.1 Hz, 1H), 7.59 (d, *J* = 15.1 Hz, 1H), 7.52 (dd, *J* = 7.6, 1.4 Hz, 1H), 7.36 (td, *J* = 7.6, 1.4 Hz, 1H), 7.21–7.19 (m, 2H); ^13^C-NMR (DMSO-*d_6_*), δ: 163.5, 138.2, 135.6, 134.8, 132.0, 131.8, 131.1, 129.6, 129.5, 127.6, 127.5, 126.1, 125.5, 125.2, 124.2 (Figure S23); HR-MS: for C_15_H_9_ONCl_3_ [M-H]^−^ calculated 323.9755 *m*/*z*, found 323.9759 *m*/*z*.

*(2E)-N-(3-Chlorophenyl)-3-(3,4-dichlorophenyl)prop-2-enamide* (**2f**). Yield 63%; Mp 186–188 °C; IR (cm^−1^): 3277 (N-H), 1664 (C=O), 1626 (C=C), 1597 (C-C_arom_); ^1^H-NMR (DMSO-*d_6_*), δ: 10.42 (s, 1H), 7.93–7.91 (m, 2H), 7.70 (d, *J* = 8.2 Hz, 1H), 7.63 (dd, *J* = 8.6, 1.7 Hz, 1H), 7.58 (d, *J* = 15.8 Hz, 1H), 7.51 (dd, *J* = 8.2, 1.4 Hz, 1H), 7.36 (t, *J* = 7.9 Hz, 1H), 7.13 (dd, *J* = 8.2, 1.4 Hz, 1H), 6.85 (d, *J* = 15.8 Hz, 1H); ^13^C-NMR (DMSO-*d_6_*), δ: 163.3, 140.5, 138.1, 135.5, 133.2, 132.1, 131.8, 131.2, 130.54, 129.8, 127.5, 124.1, 123.2, 118.7, 117.7 (Figure S24); HR-MS: for C_15_H_9_ONCl_3_ [M-H]^−^ calculated 323.9755 *m*/*z*, found 323.9755 *m*/*z*.

*(2E)-N-(4-Chlorophenyl)-3-(3,4-dichlorophenyl)prop-2-enamide* (**2g**). Yield 71%; Mp 158–160 °C; IR (cm^−1^): 3291 (N-H), 1660 (C=O), 1623 (C=C), 1590 (C-C_arom_); ^1^H-NMR (DMSO-*d_6_*), δ: 10.37 (s, 1H), 7.90 (d, *J* = 2.1 Hz, 1H), 7.73–7.70 (m, 2H), 7.70 (d, *J* = 8.2 Hz, 1H), 7.62 (dd, *J* = 8.6, 1.7 Hz, 1H), 7.57 (d, *J* = 15.8 Hz, 1H), 7.40–7.37 (m, 2H), 6.85 (d, *J* = 15.8 Hz, 1H); ^13^C-NMR (DMSO-*d_6_*), δ: 163.1, 138.0, 137.8, 135.6, 132.0, 131.8, 131.1, 129.7, 128.8, 127.4, 127.1, 124.3, 120.8 (Figure S25); HR-MS: for C_15_H_9_ONCl_3_ [M-H]^−^ calculated 323.9755 *m*/*z*, found 323.9757 *m*/*z*.

*(2E)-3-(3,4-Dichlorophenyl)-N-[2-(trifluoromethyl)phenyl]prop-2-enamide* (**2h**). Yield 65%; Mp 153–156 °C; IR (cm^−1^): 3285 (N-H), 1661 (C=O), 1628 (C=C), 1591 (C-C_arom_); ^1^H-NMR (DMSO-*d_6_*), δ: 9.72 (s, 1H), 7.94 (d, *J* = 2.1 Hz, 1H), 7.76 (dd, *J* = 7.6, 1.4 Hz, 1H), 7.72–7.70 (m, 2H), 7.66–7.64 (m, 2H), 7.58 (d, *J* = 15.8 Hz, 1H), 7.47 (t, *J* = 7.6 Hz, 1H), 7.08 (d, *J* = 15.8 Hz, 1H); ^13^C-NMR (DMSO-*d_6_*), δ: 164.1, 138.2, 135.6, 135.2, 133.0, 132.0, 131.8, 131.2, 129.7, 129.5, 127.6, 126.6, 126.3 (q, *J* = 5.8 Hz), 124.1 (q, *J* = 30.3 Hz), 123.7, 123.6 (q, *J =* 273.1 Hz) (Figure S26); HR-MS: for C_16_H_9_ONCl_2_F_3_ [M-H]^−^ calculated 358.0019 *m*/*z*, found 358.0017 *m*/*z*.

*(2E)-3-(3,4-Dichlorophenyl)-N-[3-(trifluoromethyl)phenyl]prop-2-enamide* (**2i**). Yield 72%; Mp 156–159 °C; IR (cm^−1^): 3310 (N-H), 1666 (C=O), 1626 (C=C), 1604 (C-C_arom_); ^1^H-NMR (DMSO-*d_6_*), δ: 10.58 (s, 1H), 8.20 (s, 1H), 7.92 (d, *J* = 2.1 Hz, 1H), 7.85 (d, *J* = 8.2 Hz, 1H), 7.71 (d, *J* = 8.2 Hz, 1H), 7.64 (dd, *J* = 8.2, 2.1 Hz, 1H), 7.62–7.60 (d, *J* = 15.8 Hz, 1H), 7.58 (t, *J* = 8.2 Hz, 1H), 7.43 (d, *J* = 8.2 Hz, 1H), 6.86 (d, *J* = 15.8 Hz, 1H); ^13^C-NMR (DMSO-*d_6_*), δ: 163.5, 139.8, 138.2, 135.4, 132.1, 131.8, 131.2, 130.1, 129.8, 129.5 (q, *J* = 31.8 Hz), 127.5, 124.1 (q, *J* = 273.1 Hz), 124.0, 122.8, 119.8 (q, *J* = 4.3 Hz), 115.3 (q, *J* = 4.3 Hz) (Figure S27); HR-MS: for C_16_H_9_ONCl_2_F_3_ [M-H]^−^ calculated 358.0019 *m*/*z*, found 358.0015 *m*/*z*.

*(2E)-3-(3,4-Dichlorophenyl)-N-[4-(trifluoromethyl)phenyl]prop-2-enamide* (**2j**). Yield 65%; Mp 162–164 °C; IR (cm^−1^): 3322 (N-H), 1668 (C=O), 1635 (C=C), 1600 (C-C_arom_); ^1^H-NMR (DMSO-*d_6_*), δ: 10.59 (s, 1H), 7.92 (d, *J* = 2.1 Hz, 1H), 7.90 (d, *J* = 8.2 Hz, 2H), 7.72–7.70 (m, 3H), 7.64 (dd, *J* = 8.9, 2.1 Hz, 1H), 7.62 (d, *J* = 15.8 Hz, 1H), 6.89 (d, *J* = 15.8 Hz, 1H); ^13^C-NMR (DMSO-*d_6_*), δ: 163.6, 142.6, 138.4, 135.5, 132.1, 131.8, 131.2, 129.8, 127.5, 126.2 (q, *J* = 4.3 Hz), 124.4 (q, *J* = 270.2 Hz), 124.1, 123.5 (q, *J* = 31.8 Hz), 119.2 (Figure S28); HR-MS: for C_16_H_9_ONCl_2_F_3_ [M-H]^−^ calculated 358.0019 *m*/*z*, found 358.0016 *m*/*z*.

*(2E)-3-(3,4-Dichlorophenyl)-N-[4-(trifluoromethoxy)phenyl]prop-2-enamide* (**2k**). Yield 61%; Mp 147–149 °C; IR (cm^−1^): 3270 (N-H), 1661 (C=O), 1626 (C=C), 1537 (C-C_arom_); ^1^H-NMR (DMSO-*d_6_*), δ: 10.44 (s, 1H), 7.91 (d, *J* = 2.1 Hz, 1H), 7.81–7.79 (m, 2H), 7.71 (d, *J* = 8.2 Hz, 1H), 7.63 (dd, *J* = 8.2, 2.1 Hz, 1H), 7.59 (d, *J* = 15.8 Hz, 1H), 7.35 (d, *J* = 8.2 Hz, 2H), 6.87 (d, *J* = 15.8 Hz, 1H); ^13^C-NMR (DMSO-*d_6_*), δ: 163.2, 143.7, 138.3, 137.9, 135.6, 132.0, 131.8, 131.2, 129.7, 127.4, 124.2, 121.7, 120.6, 120.1 (q, *J* = 255.8 Hz) (Figure S29); HR-MS: for C_16_H_9_O_2_NCl_2_F_3_ [M-H]^−^ calculated 373.9968 *m*/*z*, found 373.9965 *m*/*z*.

*(2E)-3-(3,4-Dichlorophenyl)-N-(2,4-difluorophenyl)prop-2-enamide* (**2l**). Yield 72%; Mp 147–150 °C; IR (cm^−1^): 3244 (N-H), 1659 (C=O), 1612 (C=C), 1554 (C-C_arom_); ^1^H-NMR (DMSO-*d_6_*), δ: 9.98 (s, 1H), 8.07–8.03 (m, 1H), 7.90 (d, *J* = 2.1 Hz, 1H), 7.71 (d, *J* = 8.2 Hz, 1H), 7.62 (dd, *J* = 8.2, 2.1 Hz, 1H), 7.57 (d, *J* = 15.8 Hz, 1H), 7.35 (ddd, *J* = 11.2, 8.8, 2.7 Hz, 1H), 7.11–7.07 (m, 2H); ^13^C-NMR (DMSO-*d_6_*), δ: 163.4, 158.4 (dd, *J* = 244.2, 11.6 Hz), 153.6 (dd, *J* = 248.5, 13.0 Hz), 138.0, 135.6, 132.0, 131.8, 131.2, 129.6, 127.5, 124.9 (d, *J* = 8.7 Hz), 123.9, 122.8 (dd, *J* = 11.6, 2.9 Hz), 111.2 (dd, *J* = 21.7, 2.9 Hz), 104.2 (multiplet) (Figure S30); HR-MS: for C_15_H_8_ONCl_2_F_2_ [M-H]^−^ calculated 325.9956 *m*/*z*, found 325.9957 *m*/*z*.

*(2E)-3-(3,4-Dichlorophenyl)-N-(3,5-difluorophenyl)prop-2-enamide* (**2m**). Yield 66%; Mp 170–172 °C; IR (cm^−1^): 3260 (N-H), 1668 (C=O), 1627 (C=C), 1570 (C-C_arom_); ^1^H-NMR (DMSO-*d_6_*), δ: 10.60 (s, 1H), 7.91 (d, *J* = 2.1 Hz, 1H), 7.70 (d, *J* = 8.9 Hz, 1H), 7.63 (dd, *J* = 8.9, 2.1 Hz, 1H), 7.60 (d, *J* = 15.8 Hz, 1H), 7.39 (dd, *J* = 9.6, 2.1 Hz, 2H), 6.93 (tt, *J* = 9.4, 2.3 Hz, 1H), 6.81 (d, *J* = 15.8 Hz, 1H); ^13^C-NMR (DMSO-*d_6_*), δ: 163.6, 162.5 (dd, *J* = 242.8, 15.9 Hz), 141.5 (t, *J* = 13.0 Hz), 138.6, 135.4, 132.2, 131.8, 131.2, 129.8, 127.5, 123.7, 102.1 (dd, *J* = 23.1, 5.8 Hz), 98.7 (t, *J* = 26.0 Hz) (Figure S31); HR-MS: for C_15_H_8_ONCl_2_F_2_ [M-H]^−^ calculated 325.9956 *m*/*z*, found 325.9955 *m*/*z*.

*(2E)-N-(2,4-Dichlorophenyl)-3-(3,4-dichlorophenyl)prop-2-enamide* (**2n**). Yield 64%; Mp 190–193 °C; IR (cm^−1^): 3276 (N-H), 1658 (C=O), 1626 (C=C), 1579 (C-C_arom_); NMR (DMSO-*d_6_*), δ: 9.72 (s, 1H), 8.01 (d, *J* = 8.9 Hz, 1H), 7.92 (d, *J* = 2.1 Hz, 1H), 7.71 (d, *J* = 8.2 Hz, 1H), 7.68 (d, *J* = 2.1 Hz, 1H), 7.63 (dd, *J* = 8.6, 1.7 Hz, 1H), 7.59 (d, *J* = 15.8 Hz, 1H), 7.44 (dd, *J* = 8.9, 2.1 Hz, 1H), 7.19 (d, *J* = 15.8 Hz, 1H); ^13^C-NMR (DMSO-*d_6_*), δ: 163.5, 138.4, 135.6, 134.0, 132.1, 131.8, 131.1, 129.6, 129.1, 128.9, 127.6, 127.6, 126.2, 126.0, 123.9 (Figure S32); HR-MS: for C_15_H_8_ONCl_4_ [M-H]^−^ calculated 357.9365 *m*/*z*, found 357.9373 *m*/*z*.

*(2E)-N-(2,5-Dichlorophenyl)-3-(3,4-dichlorophenyl)prop-2-enamide* (**2o**). Yield 72%; Mp 203–207 °C; IR (cm^−1^): 3398 (N-H), 3115, 1696 (C=O), 1633 (C=C), 1581 (C-C_arom_); ^1^H-NMR (DMSO-*d_6_*), δ: 9.74 (s, 1H), 8.15 (d, *J* = 2.7 Hz, 1H), 7.94 (d, *J* = 1.4 Hz, 1H), 7.72 (d, *J* = 8.2 Hz, 1H), 7.64 (dd, *J* = 8.2, 2.1 Hz, 1H), 7.60 (d, *J* = 15.8 Hz, 1H), 7.56 (d, *J* = 8.9 Hz, 1H), 7.26 (dd, *J* = 8.6, 2.4 Hz, 1H), 7.24 (d, *J* = 15.8 Hz, 1H); ^13^C-NMR (DMSO-*d_6_*), δ: 163.7, 138.7, 136.0, 135.5, 132.2, 131.8, 131.6, 131.2, 130.9, 129.7, 127.7, 125.5, 123.8, 123.5 (Figure S33); HR-MS: for C_15_H_8_ONCl_4_ [M-H]^−^ calculated 357.9365 *m*/*z*, found 357.9370 *m*/*z*.

*(2E)-N-(3,5-Dichlorophenyl)-3-(3,4-dichlorophenyl)prop-2-enamide* (**2p**). Yield 59%; Mp 169–172 °C; IR (cm^−1^): 3449 (N-H), 1659 (C=O), 1620 (C=C), 1587 (C-C_arom_); ^1^H-NMR (DMSO-*d_6_*), δ: 10.56 (s, 1H), 7.90 (d, *J* = 2.1 Hz, 1H), 7.73–7.72 (m, 2H), 7.69 (d, *J* = 8.9 Hz, 1H), 7.62 (dd, *J* = 8.9, 2.1 Hz, 1H), 7.59 (d, *J* = 15.8 Hz, 1H), 7.28–7.27 (m, 1H), 6.79 (d, *J* = 15.8 Hz, 1H); ^13^C-NMR (DMSO-*d_6_*), δ: 163.6, 141.4, 138.7, 135.3, 134.2, 132.2, 131.8, 131.2, 129.8, 127.5, 123.6, 122.7, 117.4 (Figure S34); HR-MS: for C_15_H_8_ONCl_4_ [M-H]^−^ calculated 357.9365 *m*/*z*, found 357.9372 *m*/*z*.

*(2E)-N-[3,5-bis(Trifluoromethyl)phenyl]-3-(3,4-dichlorophenyl)prop-2-enamide* (**2q**). Yield 58%; Mp 168–170 °C; IR (cm^−1^): 3413 (N-H), 1697 (C=O), 1637 (C=C), 1549 (C-C_arom_); ^1^H-NMR (DMSO-*d_6_*), δ: 10.88 (s, 1H), 8.32 (s, 2H), 7.91 (d, *J* = 2.1 Hz, 1H), 7.75 (s, 1H), 7.69 (d, *J* = 8.2 Hz, 1H), 7.63 (dd, *J* = 8.2, 2.1 Hz, 1H), 7.62 (d, *J* = 15.8 Hz, 1H), 6.80 (d, *J* = 15.8 Hz, 1H); ^13^C-NMR (DMSO-*d_6_*), δ: 163.9, 140.9, 139.0, 135.2, 132.4, 131.8, 131.2, 130.8 (q, *J* = 33.2 Hz), 129.9, 127.5, 123.4, 123.2 (q, *J* = 273.1 Hz), 118.9 (q, *J* = 2.9 Hz), 116.2 (q, *J* = 4.3 Hz) (Figure S35); HR-MS: for C_17_H_8_ONCl_2_F_6_ [M-H]^−^ calculated 425.9893 *m*/*z*, found 425.9900 *m*/*z*.

*(2E)-N-[2-Bromo-4-(trifluoromethoxy)phenyl]-3-(3,4-dichlorophenyl)prop-2-enamide* (**2r**). Yield 64%; Mp 156–158 °C; IR (cm^−1^): 3278 (N-H), 1661 (C=O), 1626 (C=C), 1585 (C-C_arom_); ^1^H-NMR (DMSO-*d_6_*), δ: 9.73 (s, 1H), 7.95–7.93 (m, 2H), 7.79 (d, *J* = 2.7 Hz, 1H), 7.72 (d, *J* = 8.2 Hz, 1H), 7.65 (dd, *J* = 8.2, 2.1 Hz, 1H), 7.61 (d, *J* = 15.8 Hz, 1H), 7.46 (dd, *J* = 8.6, 1.7 Hz, 1H), 7.16 (d, *J* = 15.8 Hz, 1H); ^13^C-NMR (DMSO-*d_6_*), δ: 163.6, 145.0, 138.5, 135.7, 135.5, 132.1, 131.8, 131.2, 129.7, 127.7, 127.1, 125.6, 123.8, 121.0, 120.0 (q, *J* = 257.2 Hz), 117.3 (Figure S36); HR-MS: for C_16_H_8_O_2_NCl_2_F_3_ [M-H]^−^ calculated 451.9073 *m*/*z*, found 451.9083 *m*/*z*.

### 3.3. Lipophilicity Determination by HPLC

A HPLC separation module Waters Alliance 2695 XE equipped with a Waters Dual Absorbance Detector 2486 (Waters Corp., Milford, MA, USA) was used. A chromatographic column Symmetry^®^ C18 5 μm, 4.6 × 250 mm, Part No. W21751W016 (Waters Corp., Milford, MA, USA) was used. The HPLC separation process was monitored by Empower^®^ 3 Chromatography Manager Software (Waters Corp.). Isocratic elution by a mixture of MeOH p.a. (72%) and H_2_O-HPLC Mili-Q grade (28%) as a mobile phase was used for the determination of capacity factor *k*. Isocratic elution by a mixture of MeOH p.a. (72%) and acetate buffered saline (pH 7.4 and pH 6.5) (28%) as a mobile phase was used for the determination of distribution coefficient expressed as *D*_7.4_ and *D*_6.5_. The total flow of the column was 1.0 mL/min, injection 20 μL, column temperature 40 °C, and sample temperature 10 °C. The detection wavelength of 210 nm was chosen. A KI methanolic solution was used for the determination of the dead times (*t_d_*). Retention times (*t_r_*) were measured in minutes. The capacity factors *k* were calculated according to the formula *k* = (*t_r_* − *t_d_*)/*t_d_*, where *t_R_* is the retention time of the solute, and *t_d_* is the dead time obtained using an unretained analyte. The distribution coefficients *D*_pH_ were calculated according to the formula *D*_pH_ = (*t_r_* − *t_d_*)/*t_d_*. Each experiment was repeated three times. The log *k* values of individual compounds are shown in Table 1.

### 3.4. In Vitro Antibacterial Evaluation

The minimum inhibitory concentrations (MICs) were evaluated by the microtitration broth method according to the CLSI [60] with some modifications. The compounds were dissolved in DMSO (Sigma, St. Louis, MO, USA) to get concentration 10 µg/mL and diluted in a microtitration plate in an appropriate medium, i.e., Cation Adjusted Mueller–Hinton (CaMH, Oxoid, Basingstoke, UK) for staphylococci, and brain heart infusion (BHI, Oxoid) for enterococci to reach the final concentration of 256–0.125 µg/mL. The plate was inoculated by tested microorganisms. The final concentration of bacterial cells was 10^5^ for bacteria. Ampicillin (Sigma) was used as reference drugs. A drug-free control and a sterility control were included. The plates were incubated for 24 h at 37 °C for staphylococci and enterococci. After static incubation in the darkness in an aerobic atmosphere, the MIC was visually evaluated as the lowest concentration of the tested compound, which completely inhibited the growth of the microorganism. The experiments were repeated three times. The results are summarized in Table 2.

### 3.5. In Vitro Antimycobacterial Evaluation

The evaluation of in vitro antimycobacterial activity of the compounds was performed against *Mycobacterium marinum* CAMP 5644 and *M. smegmatis* ATCC 700084. The broth dilution micro-method in Middlebrook 7H9 medium (Difco, Lawrence, KS, USA) supplemented with ADC Enrichment (Difco) was used to determine the minimum inhibitory concentration (MIC) as previously described [61]. The compounds were dissolved in DMSO (Sigma), and the final concentration of DMSO did not exceed 2.5% of the total solution composition. The final concentrations of the evaluated compounds ranging from 256 μg/mL to 0.125 μg/mL were obtained by twofold serial dilution of the stock solution in a microtiter plate with sterile medium. Isoniazid and rifampicin (Sigma) were used as reference antibacterial drugs. Bacterial inocula were prepared by transferring colonies from culture to sterile water. The cell density was adjusted to 0.5 McFarland units using a densitometer (Densi-La-Meter, LIAP, Riga, Latvia). The final inoculum was made by 1:1000 dilution of the suspension with sterile water. Drug-free controls, sterility controls and controls consisting of medium and DMSO alone were included. The determination of results was performed visually after three days of static incubation in the darkness at 37 °C in an aerobic atmosphere for *M. smegmatis* and after 21 days of static incubation in the darkness at 28 °C in an aerobic atmosphere for *M. marinum*. The minimum inhibitory concentrations (MICs) were defined as the lowest concentration of the compound at which no visible bacterial growth was observed. The MIC value is routinely and widely used in bacterial assays and is a standard detection limit according to the CLSI [60]. The results are summarized in Table 2.

### 3.6. In Vitro Cell Viability Analysis

Human monocytic leukemia cell line THP-1 and human synovial cell line SW982 were purchased from the European Collection of Cell Cultures (ECACC, Salisbury, UK) and were used for determination of the influence of test compounds on cell viability, as described previously [52]. These cell lines represent typical suspension and adherent cells, respectively. They were cultivated with test substances in the presence or absence of fetal bovine serum (FBS) in the cultivation medium, and the relative cell viability (the ratio between cells treated with compounds and cells treated only with DMSO) was determined by CCK-8 kit (Sigma) after 24 h.

Porcine monocyte-derived macrophages (MDMs) were prepared, as described previously [62]. Briefly, blood was collected from three-month-old pigs and peripheral blood mononuclear cells (PBMC) were isolated by gradient centrifugation (830× *g*, 40 min) using Histopaque-1077 (Sigma). CD14+ cells were isolated by positive magnetic bead selection with LS separation columns (Miltenyi Biotec, Bergisch Gladbach, Germany). Isolated monocytes were cultured in 96-well plates at a concentration of 1 × 10^5^ cells per well in 0.2 mL of DMEM supplemented with 10% FBS and 1% antibiotics (Antibiotic Antimycotic Solution 100 × 10,000 units penicillin, 10 mg streptomycin, and 25 μg amphotericin B per mL; Sigma) and incubated for five days at 37 °C in 5% CO_2_ to differentiate into macrophages. Then, MDMs were cultured for 24 h with tested substances and the relative cell viability was determined by CCK-8 kit (Sigma) following manufacturer instructions.

### 3.7. Flow Cytometry Analysis

Annexin V eFluor450 (Invitrogene, Waltham, MA, USA) with LIVE/DEAD Fixable Far Red Dead Cell Stain Kit (Invitrogene) were used for the analysis of cell death. THP-1 cells resuspended in serum-free medium and treated by two selected drugs, such as **2q** and **2k,** by IC_50_ concentrations, 0.5 µM and 1.0 µM, respectively, for 24 h. Cells were double-stained by AnnexinV eFluor 450 (1:20) and LIVE/DEAD Fixable Far Red (3:1000). Finally, the cells were analysed by flow cytometer (Amnis CellStream; Luminex, Austin, TX, USA) and the data were evaluated by CellStream Analysis 1.2.55 software. The dot plots were divided into four quadrants. Live cells (low left quadrant), apoptosis (upper-left quadrant) and necrosis as a terminal stage of cell death (upper-right quadrant) were distinguished. Experiments were performed in duplicates and in three independent repetitions.

## 4. Conclusions

A series of thirty-six 4-chlorocinnamanilides and 3,4-dichlorocinnamanilides were designed, prepared and tested against *S. aureus* ATCC 29213 and *E. faecalis* ATCC 29212 as reference strains and against MRSA and VRE isolates. Furthermore, the derivatives were tested against *M. smegmatis* ATCC 700084 and *M. marinum* CAMP 5644 as non-hazardous models of *M. tuberculosis*. Cytotoxicity was assessed against two human cancer cell lines (THP-1 and SW982) and against primary porcine monocyte-derived macrophages. (2*E*)-3-(4-Chlorophenyl)-*N*-(3,5-dichlorophenyl)prop-2-enamide (**1p**) and (2*E*)-*N*-[3,5-bis(trifluoromethyl)phenyl]-3-(4-chlorophenyl)prop-2-enamide (**1q**) were the most potent in series **1**. (2*E*)-*N*-[3,5-bis(Trifluoromethyl)phenyl]-3-(3,4-dichlorophenyl)- prop-2-enamide (**2q**), (2*E*)-3-(3,4-dichlorophenyl)-*N*-[3-(trifluoromethyl)phenyl]- prop-2-enamide (**2i**), (2*E*)-3-(3,4-dichlorophenyl)-*N*-[4-(trifluoromethyl)phenyl]prop- 2-enamide (**2j**) and (2*E*)-3-(3,4-dichlorophenyl)-*N*-[4-(trifluoromethoxy)phenyl]prop- 2-enamide (**2k**) were the most active in series **2**. Derivatives **1q**, **1p**, **2j** and **2q** showed submicromolar activity against *S. aureus* and MRSA isolates. In addition, agents from series **2** were effective against *E. faecalis* and VRE isolates and against both mycobacterial strains. By comparison with on cinnamon scaffold non-chlorinated derivatives, it can be stated that chlorination resulted in a significant extension and increase of antimicrobial activity. The lipophilicity (log *k* and log *D*) of the compounds was determined experimentally and other physicochemical properties of the prepared compounds were predicted. Based on a rational choice of parameters, general trends between structure and biological activity were outlined. It was found that the higher lipophilicity and surface properties of compounds and electron-withdrawing properties of anilide substituents have a positive effect on antibacterial activity and unfortunately cytotoxicity. However, when eukaryotic cells were cultured in fetal bovine serum, which mimics real in vivo conditions, most derivatives demonstrated cytotoxicity, expressed as IC_50_ values, above 10 μM; only compound **2q** showed cytotoxicity in the range of IC_50_ 5–10 μM. These compounds induced cell death (necrosis) of THP-1 cells in a serum-free medium. Based on all of the above-mentioned facts, the most effective agent **2q** cannot be considered as suitable for further investigation, in contrast to agents **2j**, **2k** and **2i**, which have wide antibacterial potential. Compounds **1p** and **1q** can be considered as specifically antistaphylococcal compounds.

## Figures and Tables

**Table 1:** Structures of ring-substituted (2*E*)-3-(4-chlorophenyl)-*N*-arylprop-2-enanilides **1a**–**1r**, (2*E*)-3-(3,4-dichlorophenyl)-*N*-arylprop-2-enanilides **2a**–**2r**, calculated lipophilicities (log *P*/Clog *P*), and experimentally determined log *k*, log *D*_6.5_ and log *D*_7.4_ values of investigated compounds.

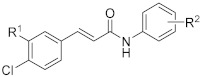								
No.	R^1^	R^2^	log *P* ^1^	log *P* ^2^	Clog *P* ^2^	log *k*	log *D*_6.5_	log *D*_7.4_
**1a**	H	H	3.84	3.74	4.3770	0.4490	0.4428	0.4515
**1b**	H	2-F	4.42	4.30	4.4976	0.6459	0.5908	0.5965
**1c**	H	3-F	4.64	4.30	5.3476	0.7859	0.7296	0.7325
**1d**	H	4-F	4.49	4.30	5.3476	0.7808	0.7252	0.7290
**1e**	H	2-Cl	3.65	3.90	4.1776	0.5241	0.4650	0.4724
**1f**	H	3-Cl	3.91	3.90	4.7776	0.6192	0.5603	0.5666
**1g**	H	4-Cl	3.76	3.90	4.7776	0.5598	0.5012	0.5065
**1h**	H	2-CF_3_	4.75	4.66	4.2608	0.5506	0.4938	0.5010
**1i**	H	3-CF_3_	4.84	4.66	5.7108	0.8612	0.8071	0.8105
**1j**	H	4-CF_3_	4.65	4.66	5.7108	0.9078	0.8541	0.8560
**1k**	H	4-OCF_3_	4.74	5.27	5.5614	0.8759	0.8702	0.8754
**1l**	H	2,4-F	3.73	4.06	4.4108	0.5746	0.5172	0.5223
**1m**	H	3,5-F	4.24	4.06	5.0108	0.8054	0.7502	0.7537
**1n**	H	2,4-Cl	5.23	4.86	5.3008	0.9665	0.9141	0.9170
**1o**	H	2,5-Cl	5.27	4.86	5.3008	0.9507	0.8986	0.9010
**1p**	H	3,5-Cl	5.40	4.86	6.1508	1.1899	1.1368	1.1366
**1q**	H	3,5-CF_3_	6.12	5.58	6.7516	1.3064	1.2925	1.2980
**1r**	H	2-Br-4-OCF_3_	5.38	6.09	5.7423	1.0278	1.0236	1.0284
**2a**	Cl	H	4.42	4.30	4.9700	0.6199	0.6669	0.6354
**2b**	Cl	2-F	5.10	4.86	5.0906	0.7764	0.8203	0.8019
**2c**	Cl	3-F	5.31	4.86	5.9406	0.9071	0.9453	0.8735
**2d**	Cl	4-F	5.19	4.86	5.9406	0.9009	0.9381	0.8660
**2e**	Cl	2-Cl	4.38	4.46	4.7706	0.6488	0.6925	0.6739
**2f**	Cl	3-Cl	4.63	4.46	5.3706	0.7404	0.7816	0.7616
**2g**	Cl	4-Cl	4.57	4.46	5.3706	0.6786	0.7224	0.7021
**2h**	Cl	2-CF_3_	5.37	5.22	4.8538	0.6836	0.7295	0.6526
**2i**	Cl	3-CF_3_	5.54	5.22	6.3038	0.9873	0.9915	0.9505
**2j**	Cl	4-CF_3_	5.35	5.22	6.3038	1.0364	1.0370	0.9945
**2k**	Cl	4-OCF_3_	5.05	5.82	6.1544	1.0513	1.0544	1.0053
**2l**	Cl	2,4-F	4.50	4.61	5.0038	0.6989	0.7094	0.6632
**2m**	Cl	3,5-F	4.97	4.61	5.6038	0.9298	0.9345	0.8882
**2n**	Cl	2,4-Cl	5.68	5.41	5.8938	1.0932	1.0985	1.0565
**2o**	Cl	2,5-Cl	5.72	5.41	5.8938	1.0840	1.0887	1.0474
**2p**	Cl	3,5-Cl	5.90	5.41	6.7438	1.3043	1.3336	1.3080
**2q**	Cl	3,5-CF_3_	6.43	6.14	7.3446	1.4731	1.4967	1.4717
**2r**	Cl	2-Br-4-OCF_3_	5.91	6.65	6.3353	1.2065	1.2373	1.2178

^1^ ACD/Percepta ver. 2012 (Advanced Chemistry Development. Inc., Toronto, ON, Canada, 2012); ^2^ ChemBioDrawUltra 13.0 (CambridgeSoft, PerkinElmer Inc., MA, USA).

**Table 2:** In vitro antistaphylococcal, antienterococcal activities (MIC [μM]) compared to ampicillin, in vitro antimycobacterial activity (MIC [μM]) compared to isoniazid and rifampicin, and cell viability (IC_50_ [µM]) on three eukaryotic cell lines (outliers detected and eliminated, Q = 5%).

No.	MIC (µM)										IC_50_ (µM)			
	SA	MRSA1	MRSA2	MRSA3	EF	VRE1	VRE2	VRE3	MS	MM	THP-1 SFM	THP-1 10% FSB	SW982 10% FSB	MDM 10% FSB
**1i**	6.14	6.14	3.07	6.14	>786	>786	>786	>786	>786	>786	2.9 ± 1.2	>10	>10	>10
**1p**	0.383	0.383	0.383	0.765	>783	>783	>783	>783	24.48	>783	2.5 ± 1.1	>10	>10	>10
**1q**	0.318	0.318	0.318	0.635	325	650	325	325	2.54	>650	1.0 ± 1.1	>10	>10	>10
**2a**	13.6	>876	>876	>876	876	>876	>876	>876	13.6	876	7.5 ± 1.0	>10	>10	>10
**2i**	1.39	0.694	1.39	1.39	22.2	22.2	22.2	44.4	5.55	5.55	1.9 ± 1.0	>10	>10	>10
**2j**	0.694	0.371	0.371	0.694	11.1	11.1	11.1	11.1	2.78	2.78	0.9 ± 1.0	>10	>10	>10
**2k**	1.33	1.33	1.33	1.33	10.6	21.3	42.5	21.3	10.6	2.66	1.0 ± 1.0	>10	>10	>10
**2q**	0.292	0.136	0.136	0.292	4.67	9.34	18.69	4.67	2.34	0.292	0.5 ± 1.0	>10	>10	5–10
**AMP**	5.72	45.8	>45.8	>45.8	11.5	11.5	11.5	5.72	–	–	–	–	–	–
**INH**	–	–	–	–	–	–	–	–	117	467	–	–	–	–
**RIF**	–	–	–	–	–	–	–	–	19.4	2.43	–	–	–	–

SA = *Staphylococcus aureus* ATCC 29213; MRSA1–3 = clinical isolates of methicillin-resistant *S. aureus* 63718, SA 630, and SA 3202 (National Institute of Public Health, Prague, Czech Republic); EF = *Enterococcus faecalis* ATCC 29213, and vancomycin-resistant enterococci VRE1–3 = VRE 342B, VRE 368, VRE 725B, MS = *M. smegmatis* ATCC 700084; MM = *M. marinum* CAMP 5644, THP-1 = human monocytic leukemia cell line, SW982 = human synovial sarcoma cell line, MDM = porcine monocyte-derived macrophages, SFM = serum-free medium, FSB = fetal bovine serum, AMP = ampicillin, INH = isoniazid, RIF = rifampicin.

**Table 3:** Values of parameters characterizing physicochemical properties calculated using ACD/Percepta ver. 2012 in relation to Lipinski’s Rule of Five (Ro5).

No.	R^1^	R^2^	MW	log *P*	HBD	HBA	RB	TPSA	Parachor (cm^3^)	σ_Ph-R2_	ST (dyne/cm)
**1i**	H	3-CF_3_	325.71	4.84	1	2	4	29.10	602.60	0.89	41.91
**1p**	H	3,5-Cl	326.60	5.40	1	2	3	29.10	617.13	1.11	54.41
**1q**	H	3,5-CF_3_	393.71	6.12	1	2	5	29.10	659.80	1.05	35.48
**2a**	Cl	H	292.16	4.42	1	2	3	29.10	581.26	0.60	53.15
**2i**	Cl	3-CF_3_	360.16	5.54	1	2	4	29.10	638.47	0.89	43.37
**2j**	Cl	4-CF_3_	360.16	5.35	1	2	4	29.10	638.47	0.95	43.37
**2k**	Cl	4-OCF_3_	376.16	5.05	1	3	5	38.33	661.54	0.62	44.82
**2q**	Cl	3,5-CF_3_	428.16	6.43	1	2	5	29.10	695.67	1.05	36.88
**Ro5**			**<500**	**<5**	**<5**	**<10**	–	–	–	–	–

MW = molecular weight, HBD = number of H-bond donors, HBA = number of H-bond acceptors, RB = number of rotatable bonds, TPSA = topological polar surface area, ST = surface tension.

## Data Availability

The data presented in this study are available on request from the corresponding author.

## Supplementary Materials

Supplementary Materials can be found at https://www.mdpi.com/article/10.3390/ijms23063159/s1.
