# Supplementary material for: Study of Biological Activities and ADMET-Related Properties of Novel Chlorinated N-arylcinnamamides
Source: Int J Mol Sci. 2022 Mar 15;23(6):3159. doi: 10.3390/ijms23063159 (PMC8951032; doi:10.3390/ijms23063159)
Supplement: Supplementary file 1 [file ijms-23-03159-s001.zip › ijms-1635927-supplementary.pdf]

Supplementary Materials

Study of Biological Activities and ADMET-Related Properties of Novel Chlorinated *N*-arylcinnamamides

Tomas Strharsky [1], Dominika Pindjakova [2], Jiri Kos [1,2,3,*], Lucia Vrablova [2], Hana Michnova [1], Jan Hosek[1], Nicol Strakova[4], Veronika Lelakova [4], Lenka Leva [5], Lenka Kavanova [5], Michal Oravec [6], Alois Cizek[7] and Josef Jampilek [1,2]

1 Regional Centre of Advanced Technologies and Materials, Czech Advanced Technology and Research Institute, Palacky University, Slechtitelu 27, 783 71 Olomouc, Czech Republic; strharsky.t@gmail.com (T.S.); michnova.hana@gmail.com (H.M.); jan.hosek@upol.cz (J.H.)

2 Department of Analytical Chemistry, Faculty of Natural Sciences, Comenius University, Ilkovicova 6, 842 15 Bratislava, Slovakia; pindjakova.dominika@gmail.com (D.P.); lucia.vrablova26@gmail.com (L.V.); josef.jampilek@gmail.com (J.J.)

3 Department of Biochemistry, Faculty of Medicine, Masaryk University, Kamenice 5, Brno 625 00, Czech Republic

4 Department of Pharmacology and Toxicology, Veterinary Research Institute, Hudcova 296/70, 621 00 Brno, Czech Republic; nicol.strakova@vri.cz (N.S.); veronika.lelakova@vri.cz (V.L.)

5 Department of Infectious Diseases and Preventive Medicine, Veterinary Research Institute, Hudcova 296/70, 621 00 Brno, Czech Republic; lenka.leva@vri.cz (L.L.), lenka.kavanova@vri.cz (L.K)

6 Global Change Research Institute CAS, Belidla 986/4a, 60300 Brno, Czech Republic; oravec.m@czechglobe.cz

7 Department of Infectious Diseases and Microbiology, Faculty of Veterinary Medicine, University of Veterinary Sciences Brno, Palackeho 1946/1, 612 42 Brno, Czech Republic; cizeka@vfu.cz

* Correspondence: jirikos85@gmail.com

Table S1. In vitro antistaphylococcal, antienterococcal activities (log(1/MIC [M])) compared to ampicillin, in vitro antimycobacterial activity (log(1/MIC [M])) compared to isoniazid and rifampicin, and cell viability (log(1/IC_{50} [M]) on three eukaryotic cell lines.

No.	log(1/MIC [M])										log(1/IC_{50} [M])			
	**SA**	**MRSA1**	**MRSA2**	**MRSA3**	**EF**	**VRE1**	**VRE2**	**VRE3**	**MS**	**MM**	**THP-1** SFM	**THP-1** 10% FSB	**SW982** 10% FSB	**MDM** 10% FSB
**1i**	5.21	5.21	5.51	5.21	<3.10	<3.10	<3.10	<3.10	<3.10	<3.10	5.54	<5.0	<5.0	<5.0
**1p**	6.42	6.42	6.42	6.12	<3.11	<3.11	<3.11	<3.11	4.61	<3.11	5.60	<5.0	<5.0	<5.0
**1q**	6.50	6.50	6.50	6.20	3.49	3.19	3.49	3.49	5.60	<3.19	6.00	<5.0	<5.0	<5.0
**2a**	4.86	<3.06	<3.06	<3.06	3.06	<3.06	<3.06	<3.06	4.86	3.06	5.12	<5.0	<5.0	<5.0
**2i**	5.86	6.16	5.86	5.86	4.65	4.65	4.65	4.35	5.26	5.26	5.72	<5.0	<5.0	<5.0
**2j**	6.16	6.43	6.43	6.16	4.95	4.95	4.95	4.95	5.56	5.56	6.05	<5.0	<5.0	<5.0
**2k**	6.53	5.88	5.88	5.88	5.33	4.67	4.37	4.67	5.63	6.53	6.30	<5.0	<5.0	<5.0
**2q**	5.88	6.87	6.87	6.53	4.97	5.03	4.73	5.33	4.97	5.58	6.00	<5.0	<5.0	5.3–5.0
**AMP**	5.24	4.34	<4.34	<4.34	4.94	4.94	4.94	5.24	–	–	–	–	–	–
**INH**	–	–	–	–	–	–	–	–	3.93	3.33	–	–	–	–
**RIF**	–	–	–	–	–	–	–	–	4.71	5.61	–	–	–	–

SA = *Staphylococcus aureus* ATCC 29213; MRSA1–3 = clinical isolates of methicillin-resistant *S. aureus* 63718, SA 630, and SA 3202 (National Institute of Public Health, Prague, Czech Republic); EF = *Enterococcus faecalis* ATCC 29213, and vancomycin-resistant enterococci VRE1–3 = VRE 342B, VRE 368, VRE 725B, MS = *M. smegmatis* ATCC 700084; MM = *M. marinum* CAMP 5644, THP-1 = human monocytic leukemia cell line, SW982 = human synovial sarcoma cell line, MDM = porcine monocyte-derived macrophages, SFM = serum-free medium, FSB = fetal bovine serum, AMP = ampicillin, INH = isoniazid, RIF = rifampicin.

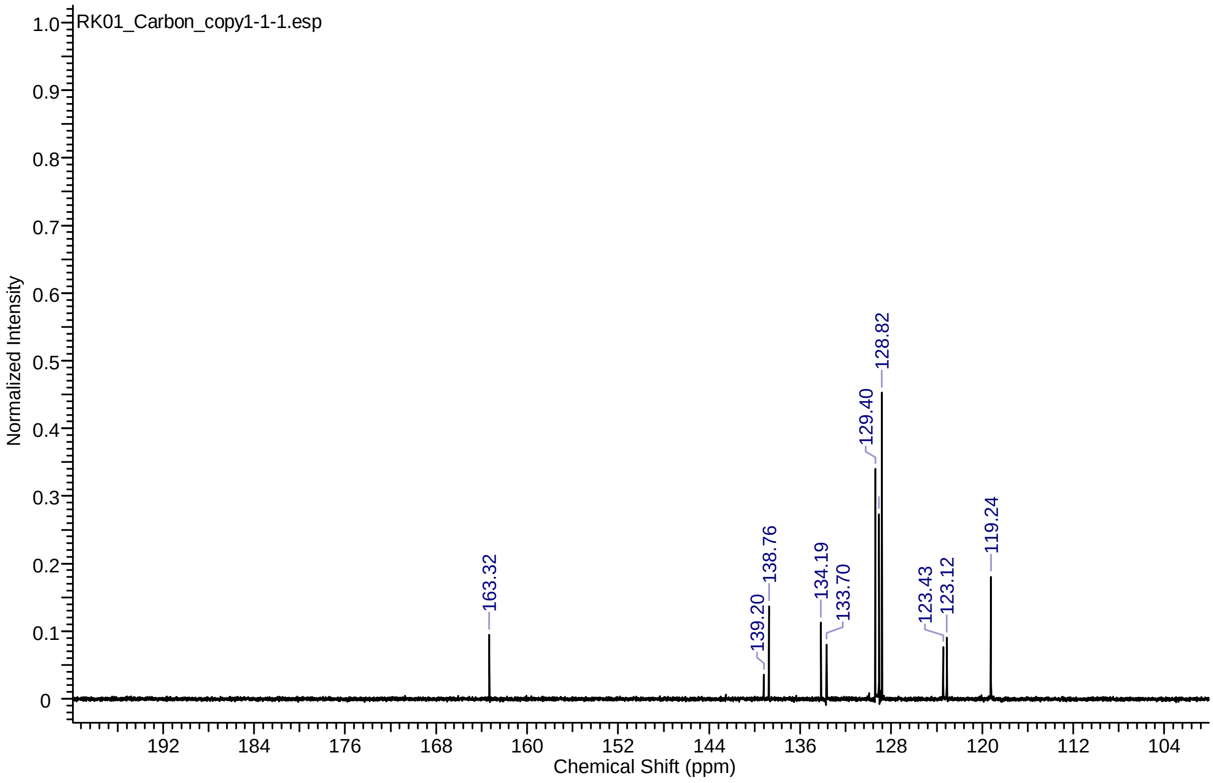

Figure S1. ^{13}C-NMR (DMSO-d_6) spectrum of (2*E*)-3-(4-chlorophenyl)-*N*-phenylprop-2-enamide (**1a**).

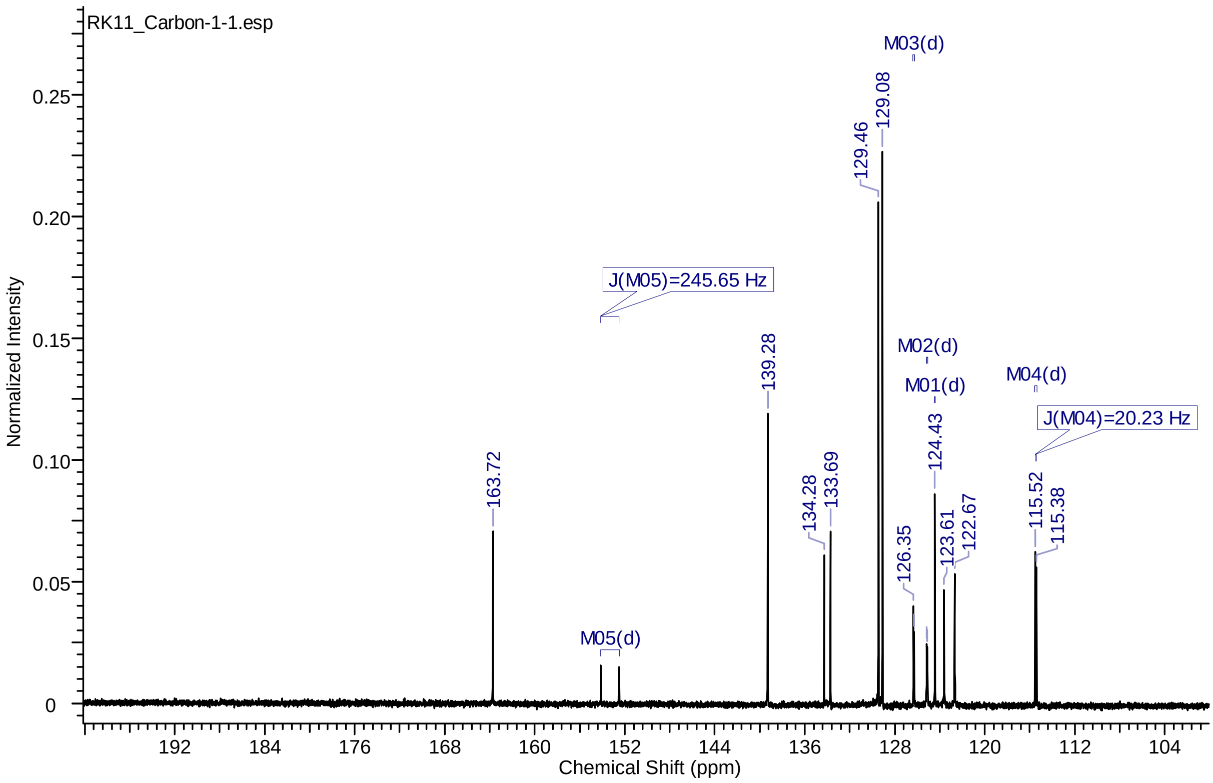

Figure S2. ^{13}C-NMR (DMSO-d_6) spectrum of (2*E*)-3-(4-chlorophenyl)-*N*-(2-fluorophenyl)prop-2-enamide (**1b**).

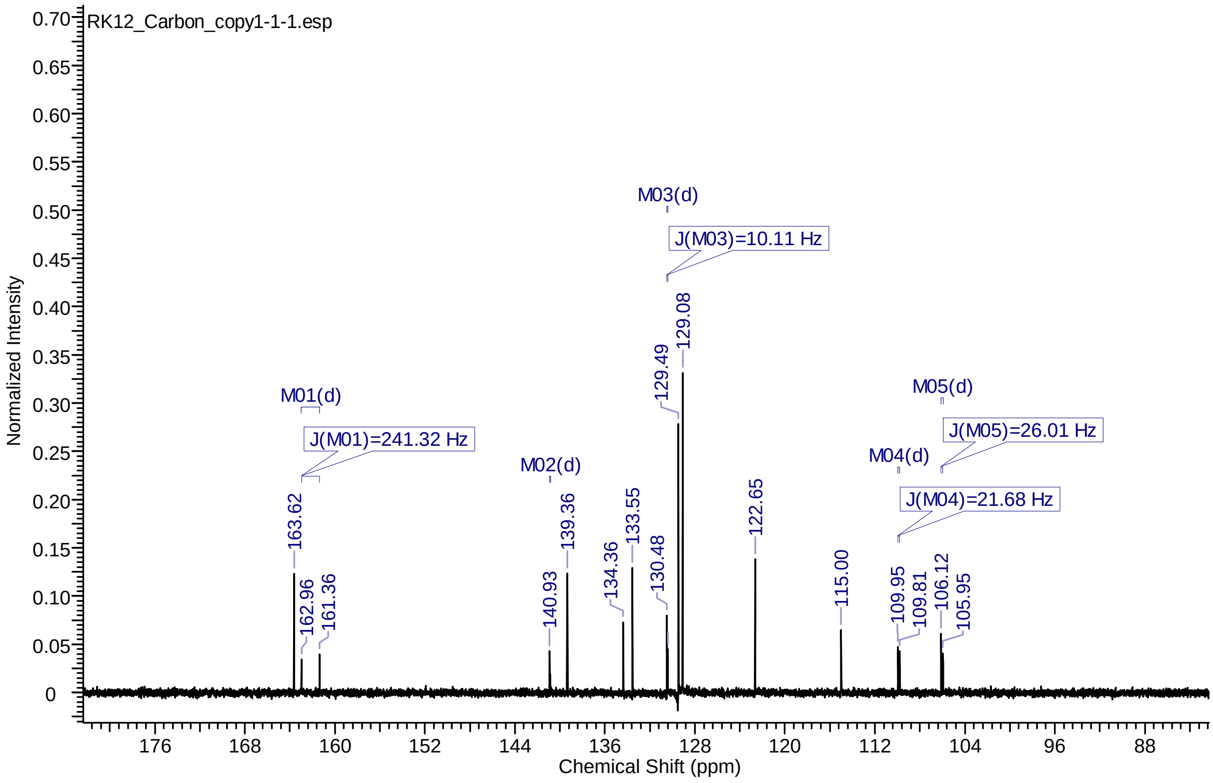

Figure S3. ^{13}C-NMR (DMSO-d_6) spectrum of (2*E*)-3-(4-chlorophenyl)-*N*-(3-fluorophenyl)prop-2-enamide (**1c**).

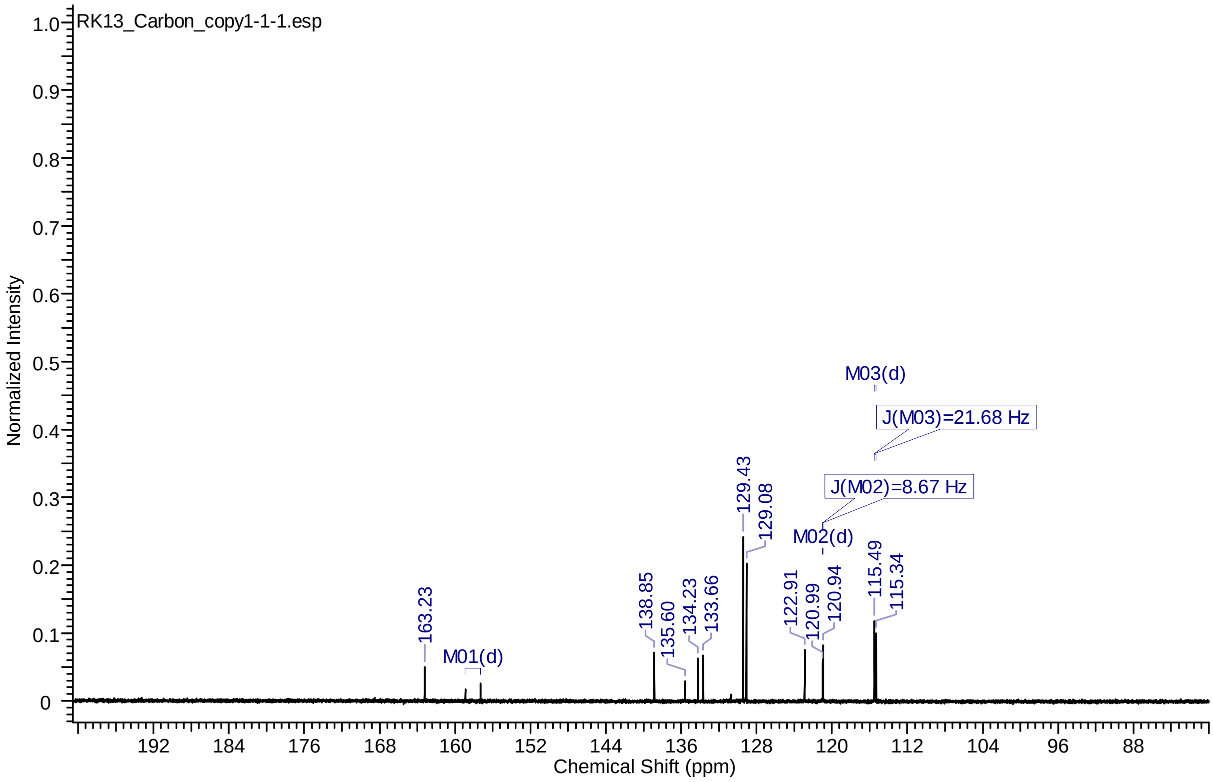

Figure S4. ^{13}C-NMR (DMSO-d_6) spectrum of (2*E*)-3-(4-chlorophenyl)-*N*-(4-fluorophenyl)prop-2-enamide (**1d**).

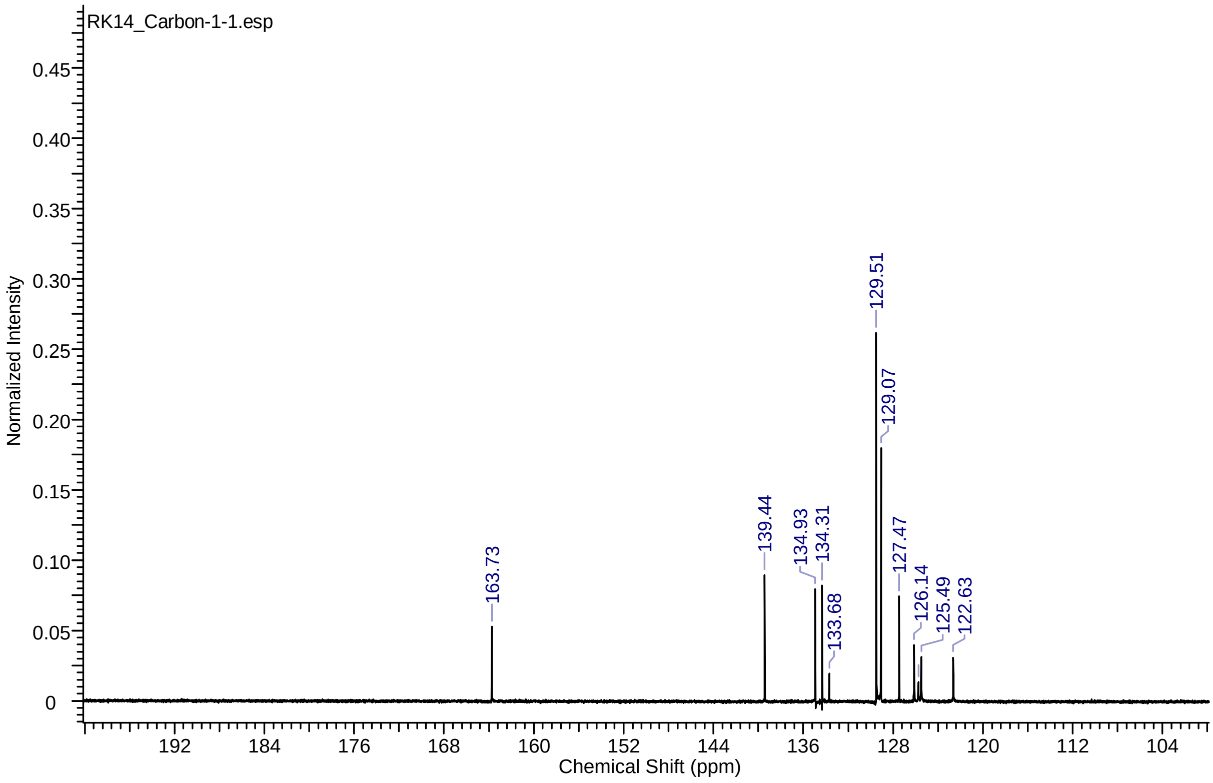

Figure S5. ^{13}C-NMR (DMSO-d_6) spectrum of (2*E*)-*N*-(2-chlorophenyl)-3-(4-chlorophenyl)prop-2-enamide (**1e**).

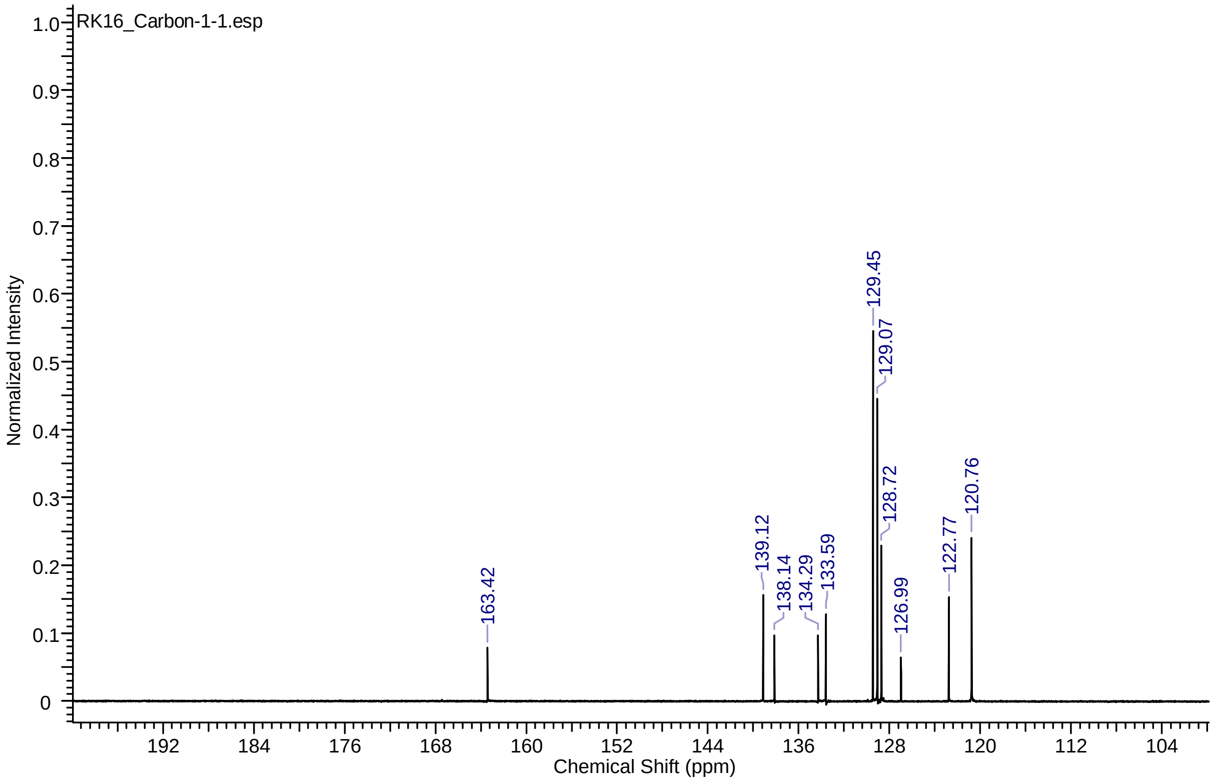

Figure S6. ^{13}C-NMR (DMSO-d_6) spectrum of (2*E*)-*N*-(3-chlorophenyl)-3-(4-chlorophenyl)prop-2-enamide (**1f**).

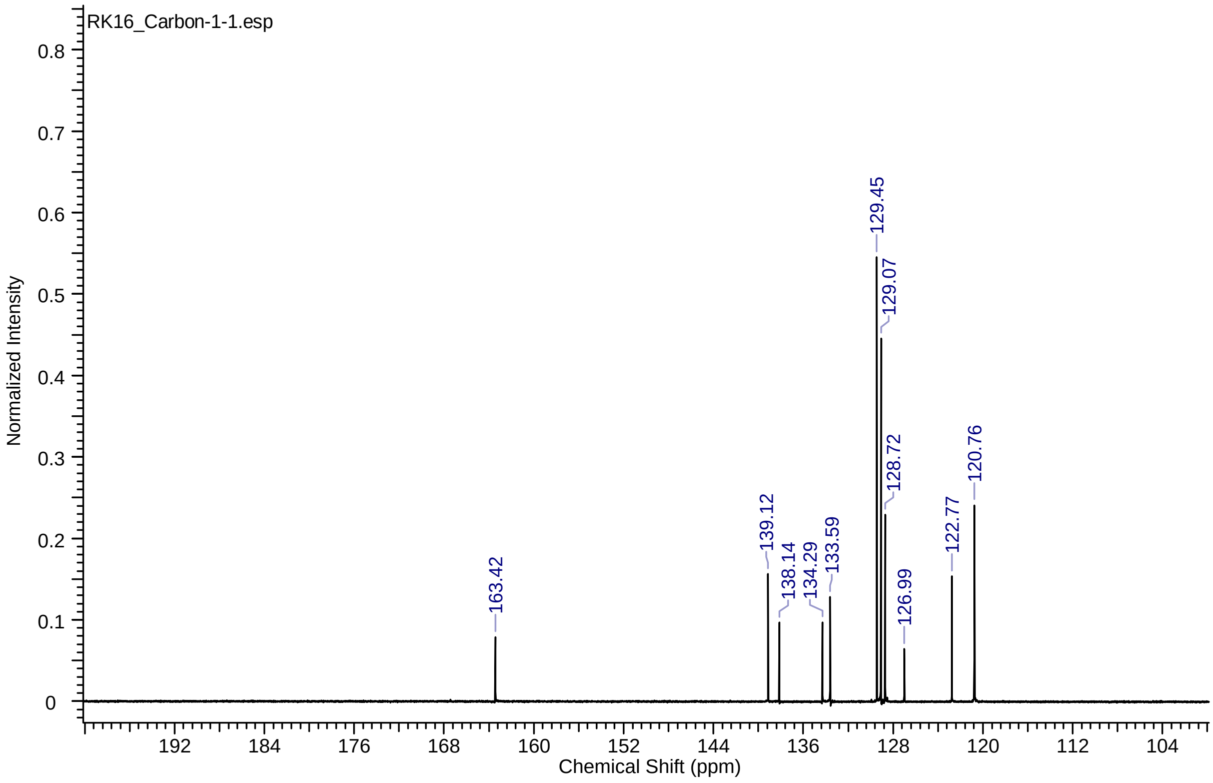

Figure S7. ^{13}C-NMR (DMSO-d_6) spectrum of (2*E*)-*N*,3-bis(4-chlorophenyl)prop-2-enamide (**1g**).

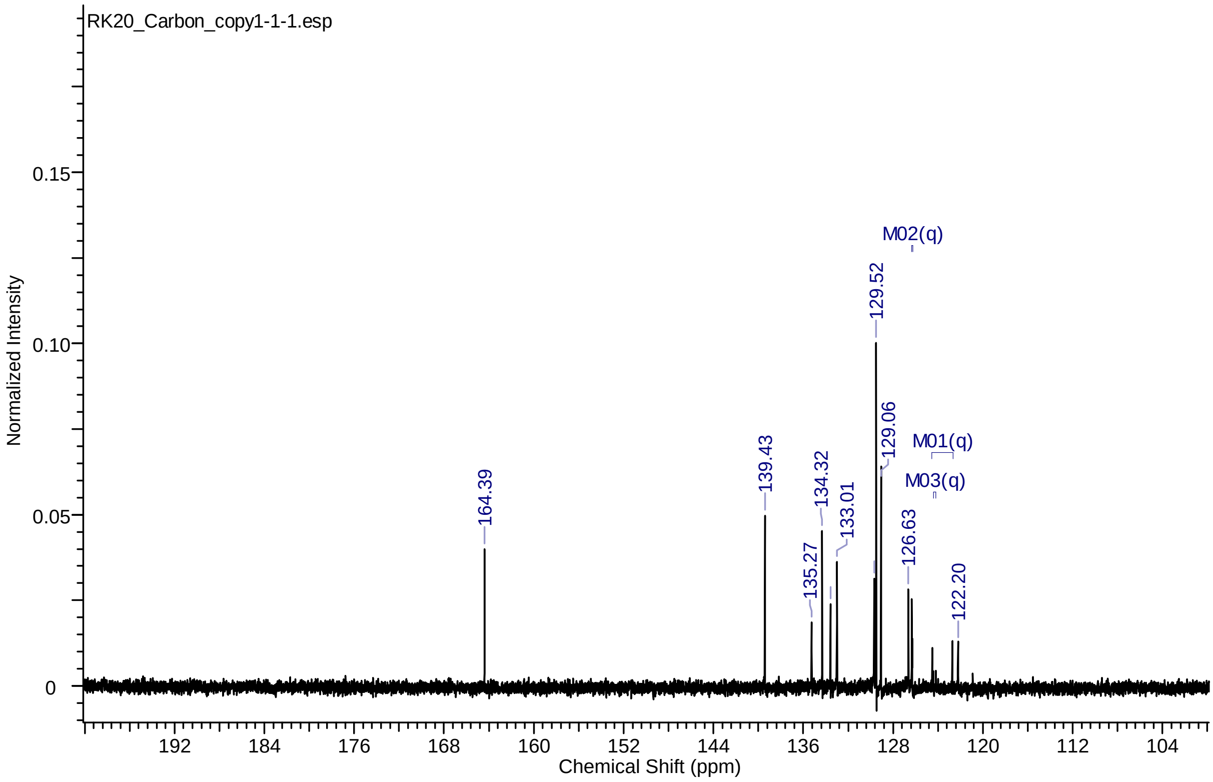

Figure S8. ^{13}C-NMR (DMSO-d_6) spectrum of (2*E*)-3-(4-chlorophenyl)-*N*-[2-(trifluoromethyl)phenyl]prop-2-enamide (**1h**).

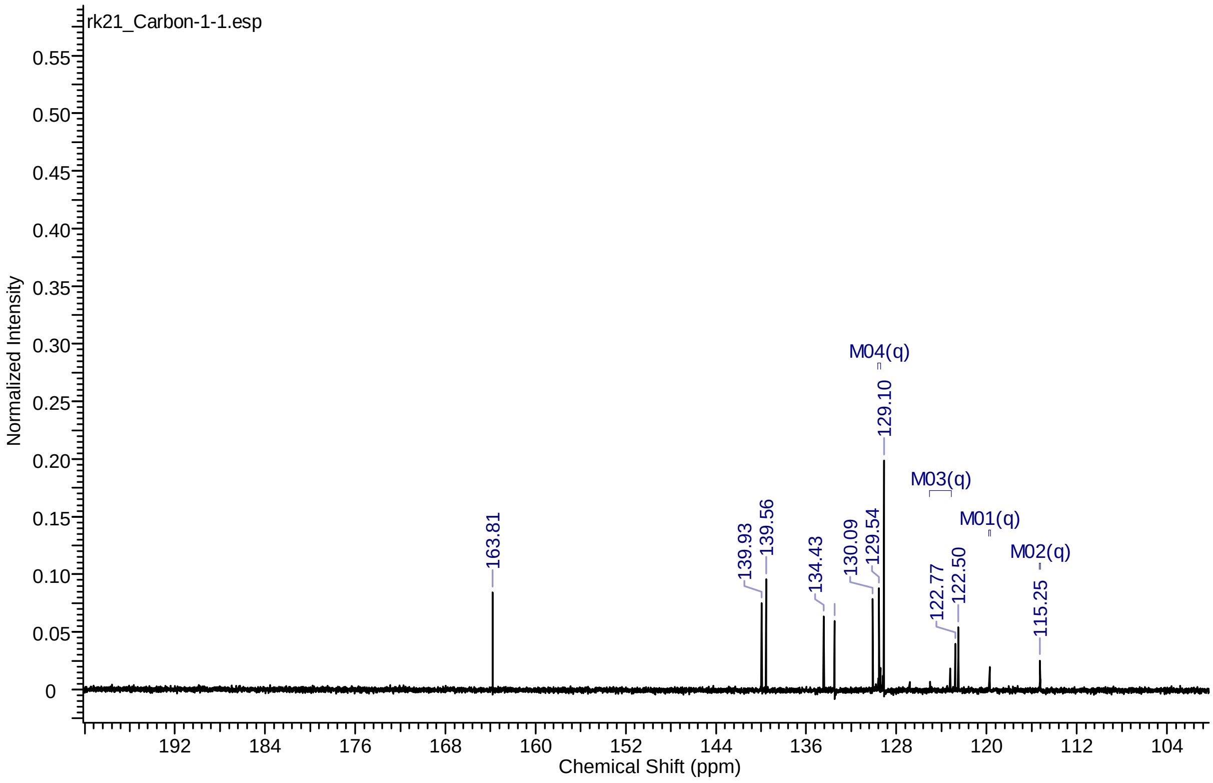

Figure S9. ^{13}C-NMR (DMSO-d_6) spectrum of (2*E*)-3-(4-chlorophenyl)-*N*-[3-(trifluoromethyl)phenyl]prop-2-enamide (**1i**).

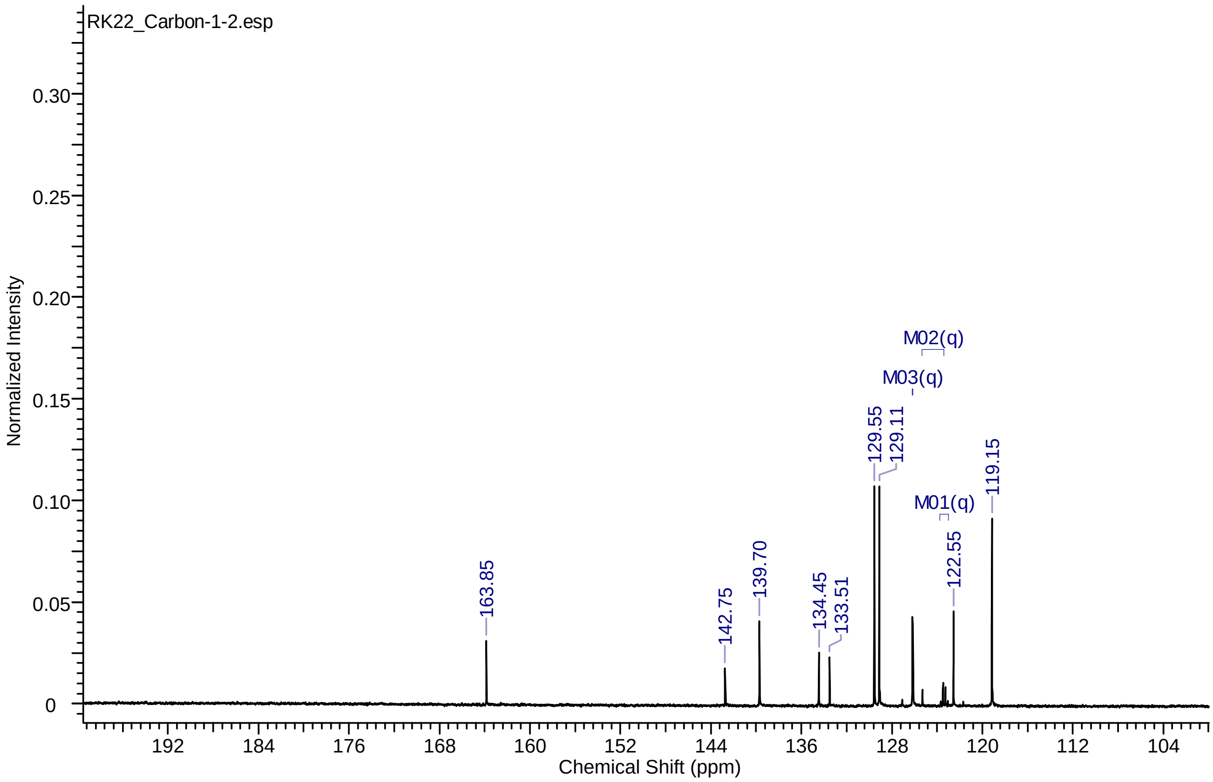

Figure S10. ^{13}C-NMR (DMSO-d_6) spectrum of (2*E*)-3-(4-chlorophenyl)-*N*-[4-(trifluoromethyl)phenyl]prop-2-enamide (**1j**).

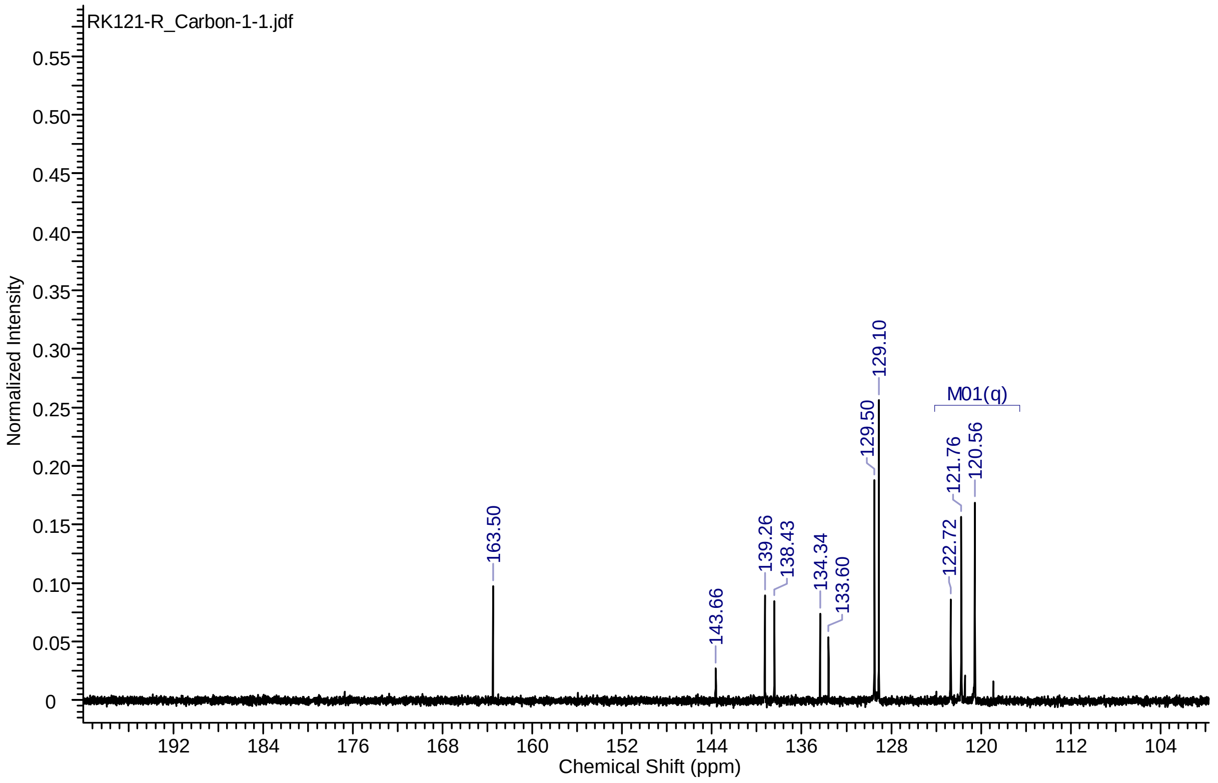

Figure S11. ^{13}C-NMR (DMSO-d_6) spectrum of (2*E*)-3-(4-chlorophenyl)-*N*-[4-(trifluoromethoxy)phenyl]prop-2-enamide (**1k**).

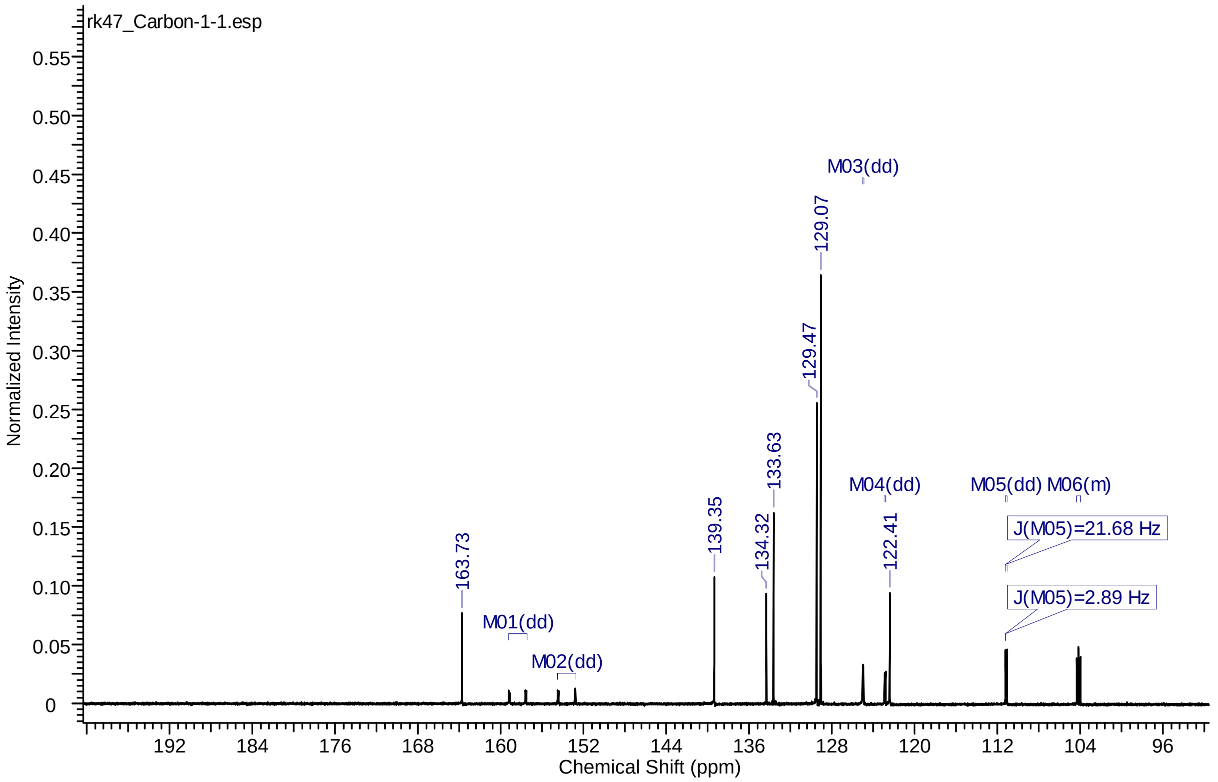

Figure S12. ^{13}C-NMR (DMSO-d_6) spectrum of (2*E*)-3-(4-chlorophenyl)-*N*-(2,4-difluorophenyl)prop-2-enamide (**1l**).

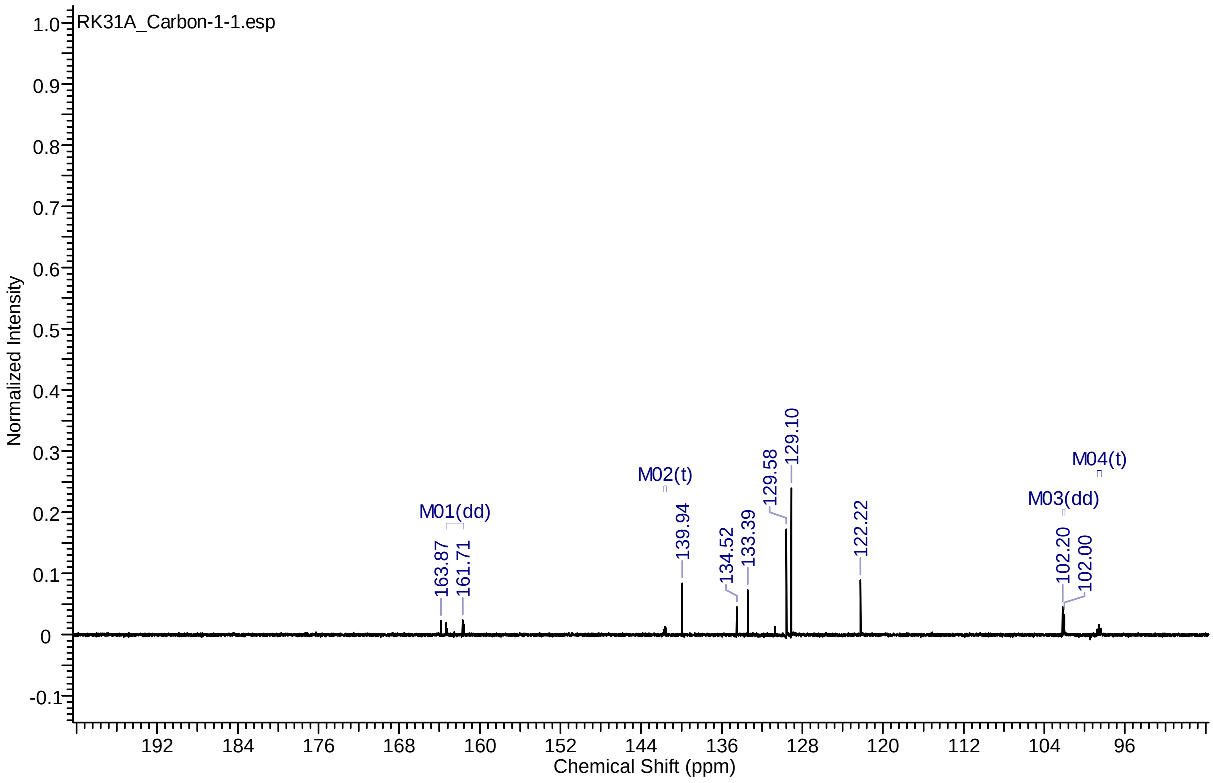

Figure S13. ^{13}C-NMR (DMSO-d_6) spectrum of (2*E*)-3-(4-chlorophenyl)-*N*-(3,5-difluorophenyl)prop-2-enamide (**1m**).

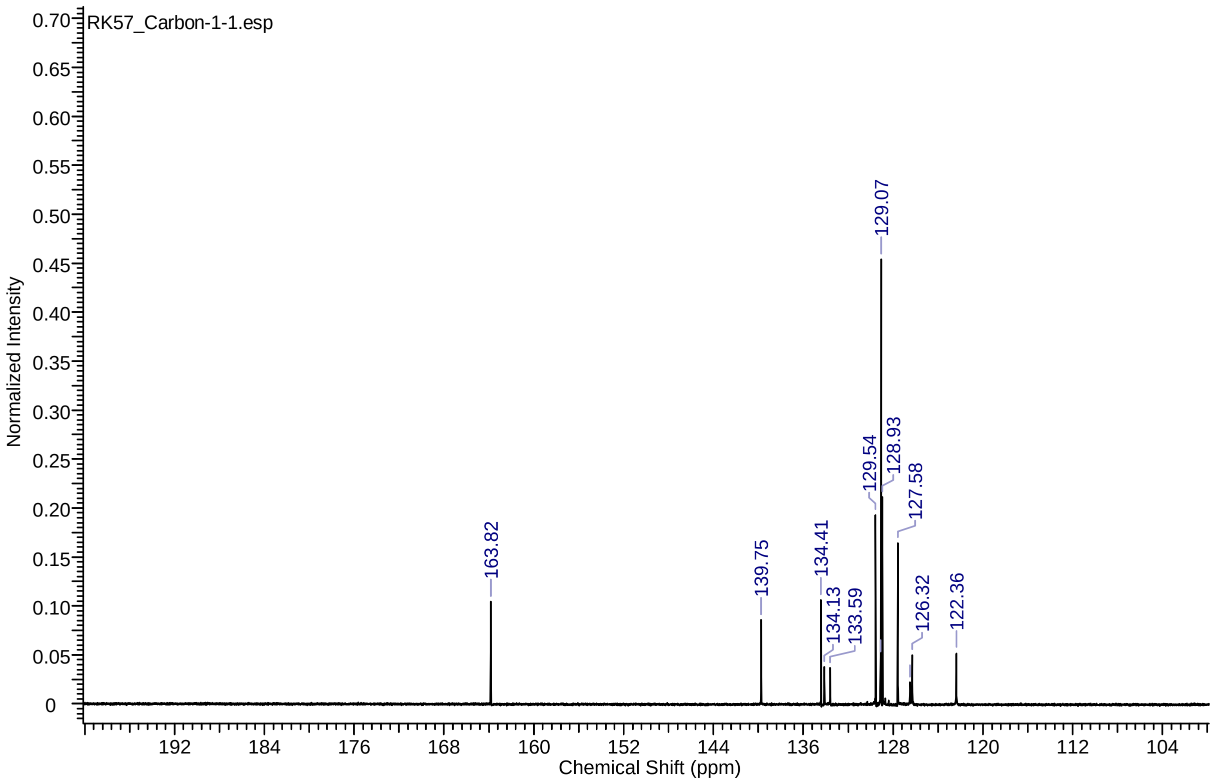

Figure S14. ^{13}C-NMR (DMSO-d_6) spectrum of (2*E*)-3-(4-chlorophenyl)-*N*-(2,4-dichlorophenyl)prop-2-enamide (**1n**).

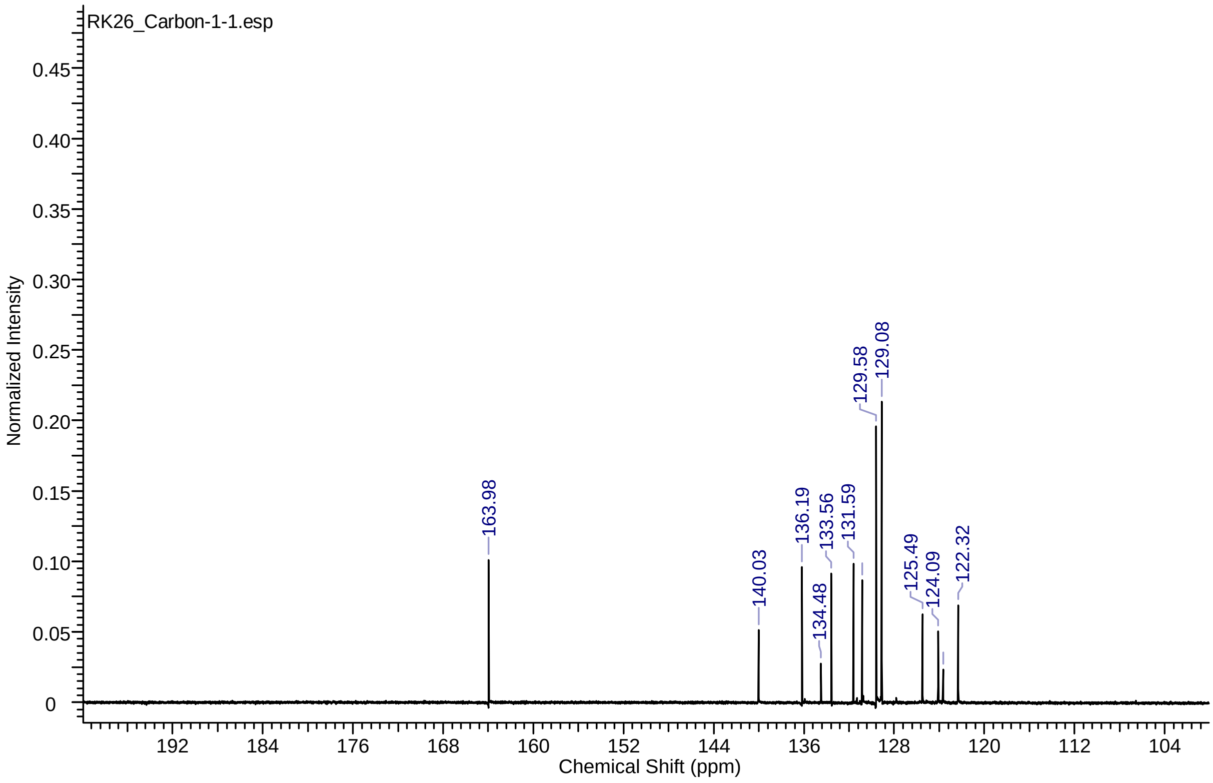

Figure S15. ^{13}C-NMR (DMSO-d_6) spectrum of (2*E*)-3-(4-chlorophenyl)-*N*-(2,5-dichlorophenyl)prop-2-enamide (**1o**).

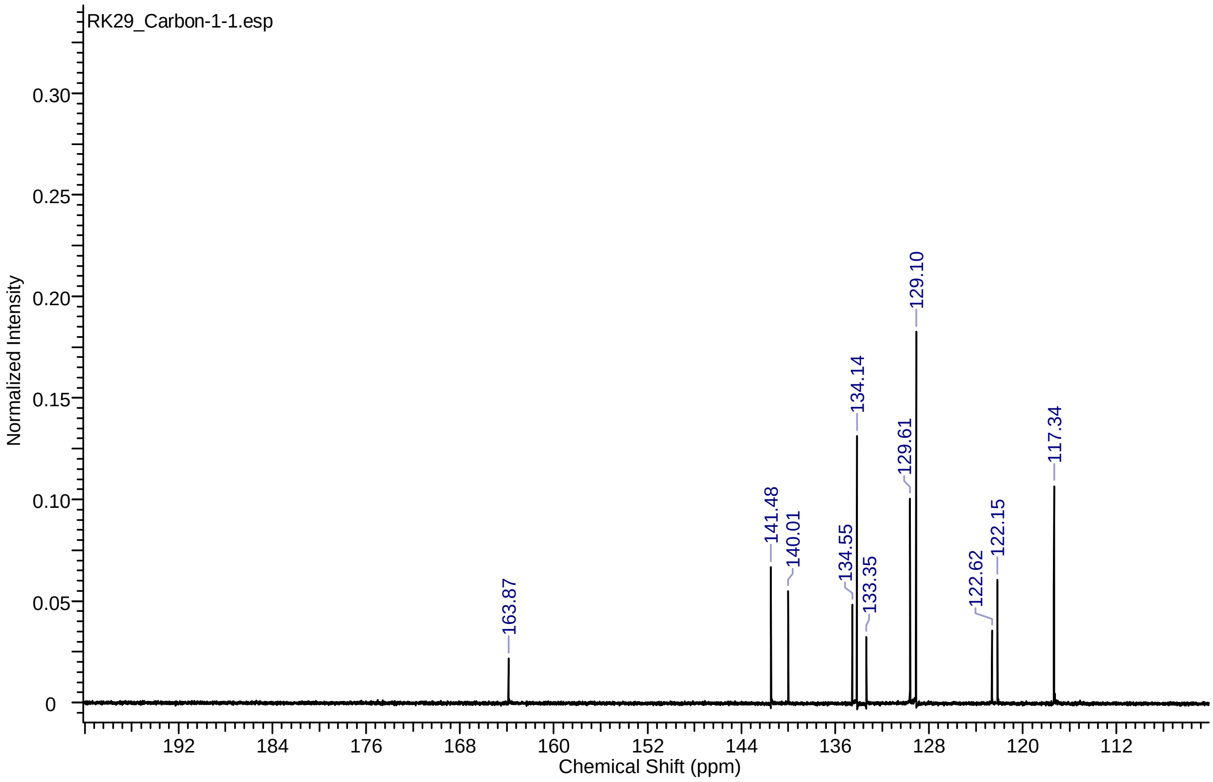

Figure S16. ^{13}C-NMR (DMSO-d_6) spectrum of (2*E*)-3-(4-chlorophenyl)-*N*-(3,5-dichlorophenyl)prop-2-enamide (**1p**).

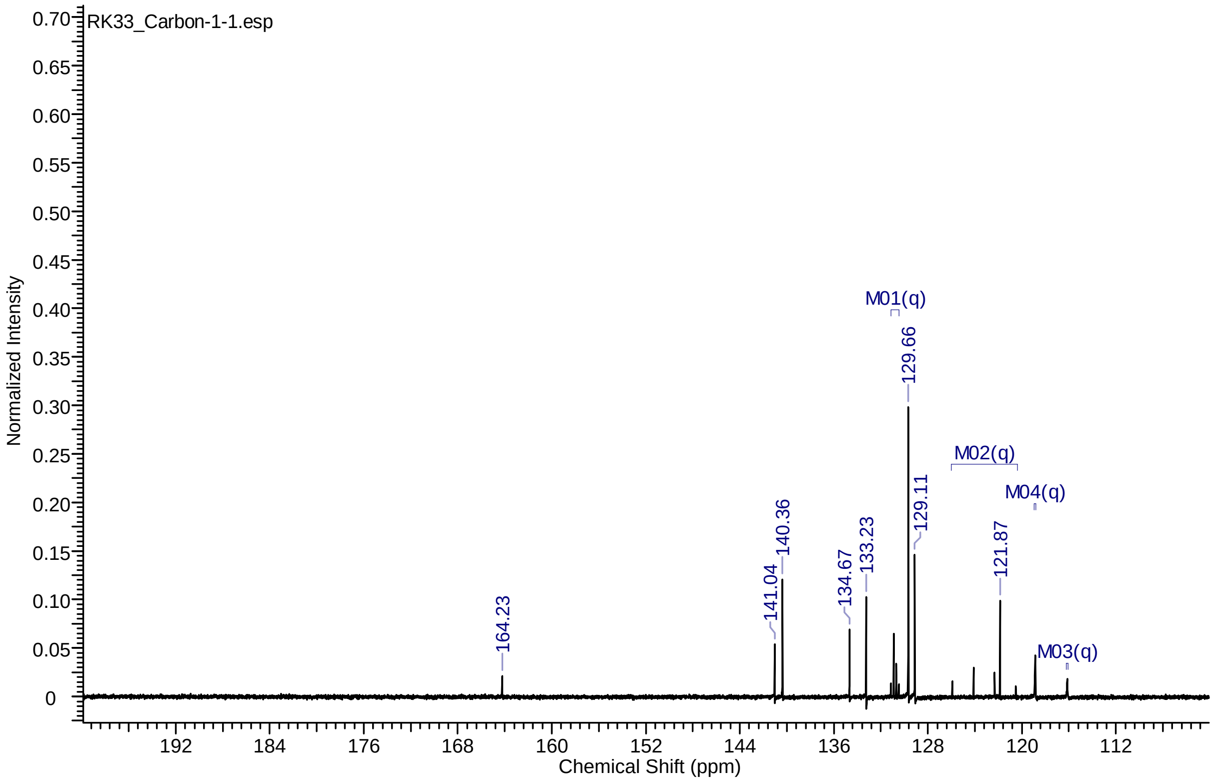

Figure S17. ^{13}C-NMR (DMSO-d_6) spectrum of (2*E*)-*N*-[3,5-bis(trifluoromethyl)phenyl]-3-(4-chlorophenyl)prop-2-enamide (**1q**).

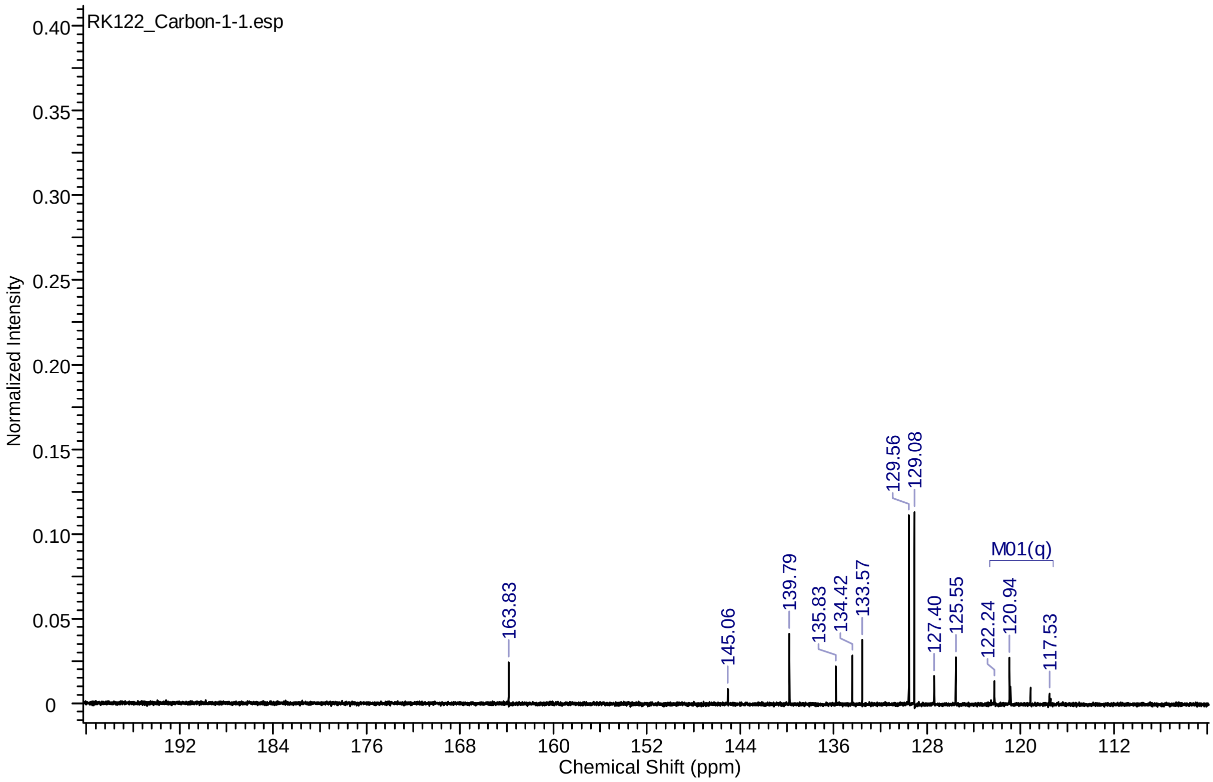

Figure S18. ^{13}C-NMR (DMSO-d_6) spectrum of (2*E*)-*N*-[2-bromo-4-(trifluoromethoxy)phenyl]-3-(4-chlorophenyl)prop-2-enamide (**1r**).

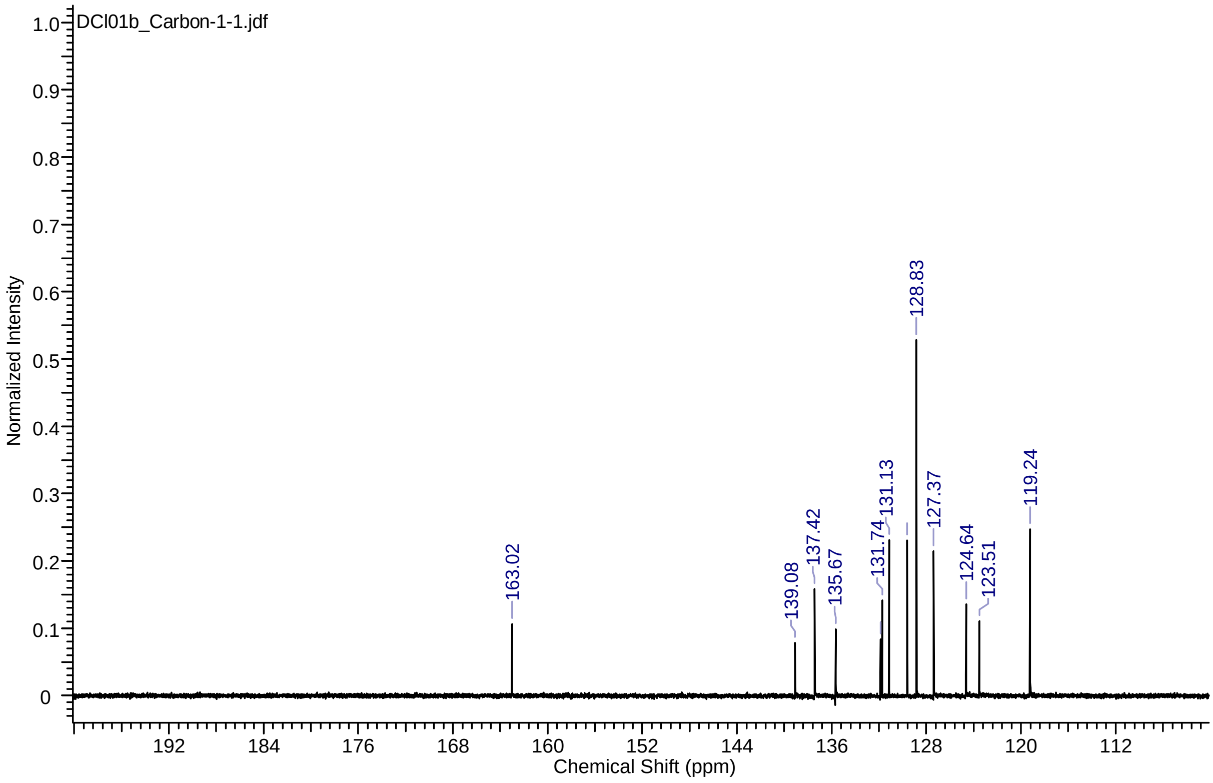

Figure S19. ^{13}C-NMR (DMSO-d_6) spectrum of (2*E*)-3-(3,4-dichlorophenyl)-*N*-phenylprop-2-enamide (**2a**).

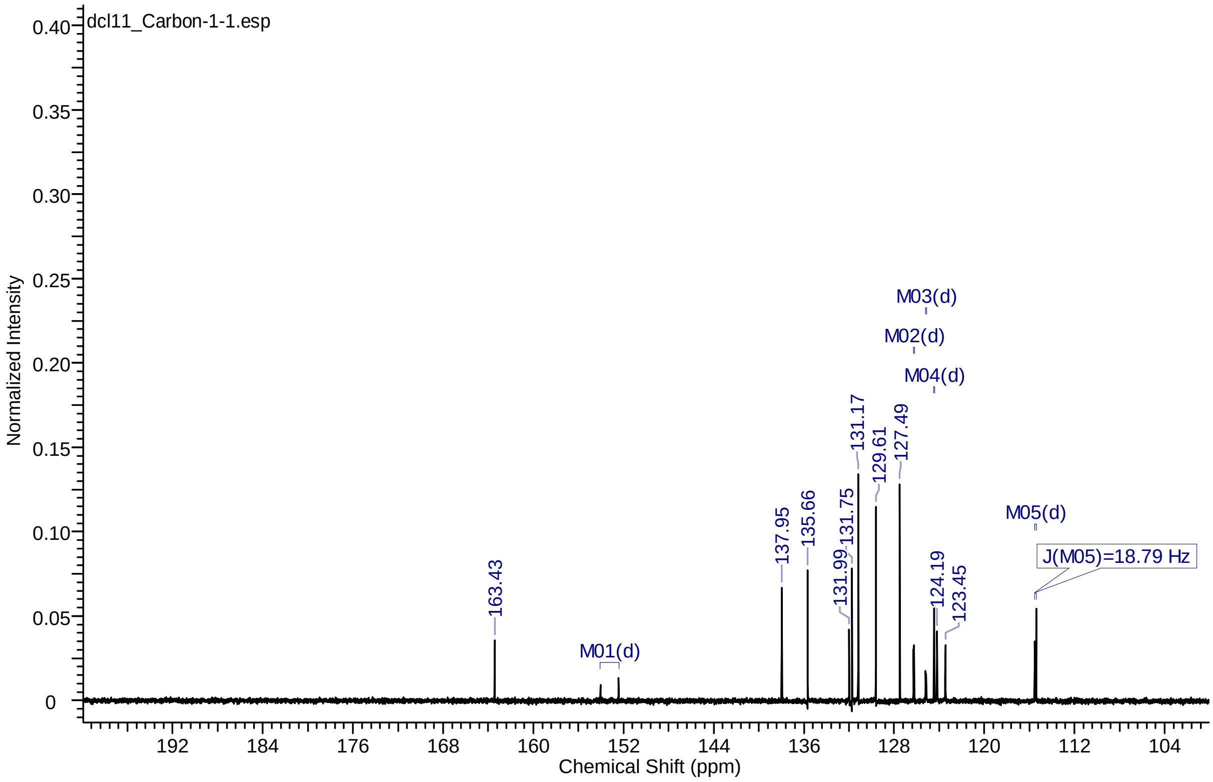

Figure S20. ^{13}C-NMR (DMSO-d_6) spectrum of (2*E*)-*N*-(2-fluorophenyl)-3-(3,4-dichlorophenyl)prop-2-enamide (**2b**).

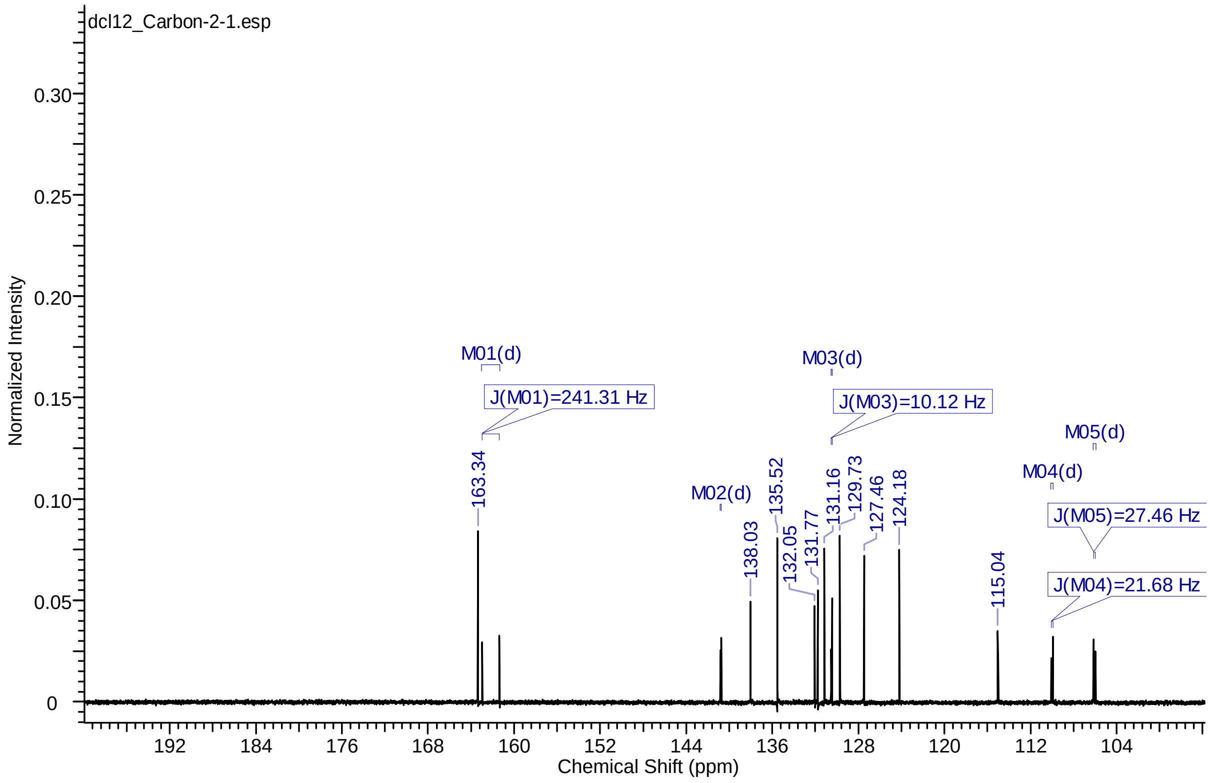

Figure S21. ^{13}C-NMR (DMSO-d_6) spectrum of (2*E*)-*N*-(3-fluorophenyl)-3-(3,4-dichlorophenyl)prop-2-enamide (**2c**).

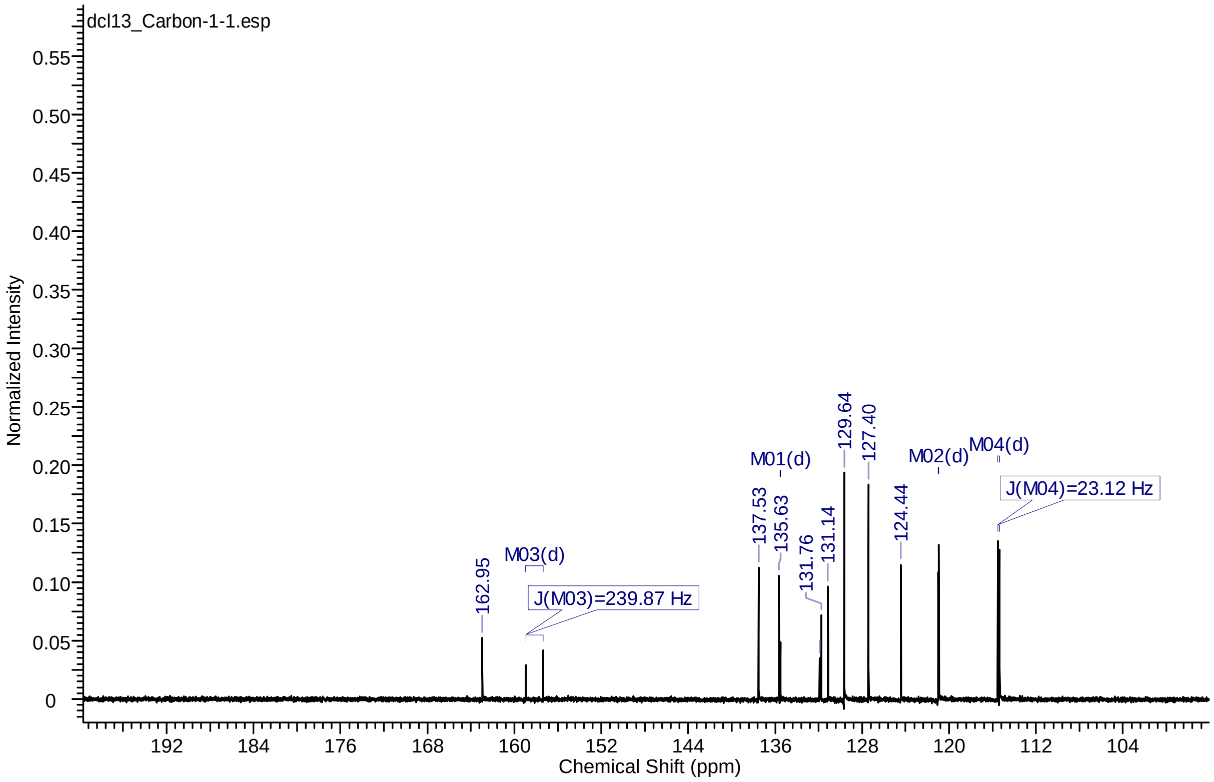

Figure S22. ^{13}C-NMR (DMSO-d_6) spectrum of (2*E*)-*N*-(4-fluorophenyl)-3-(3,4-dichlorophenyl)prop-2-enamide (**2d**).

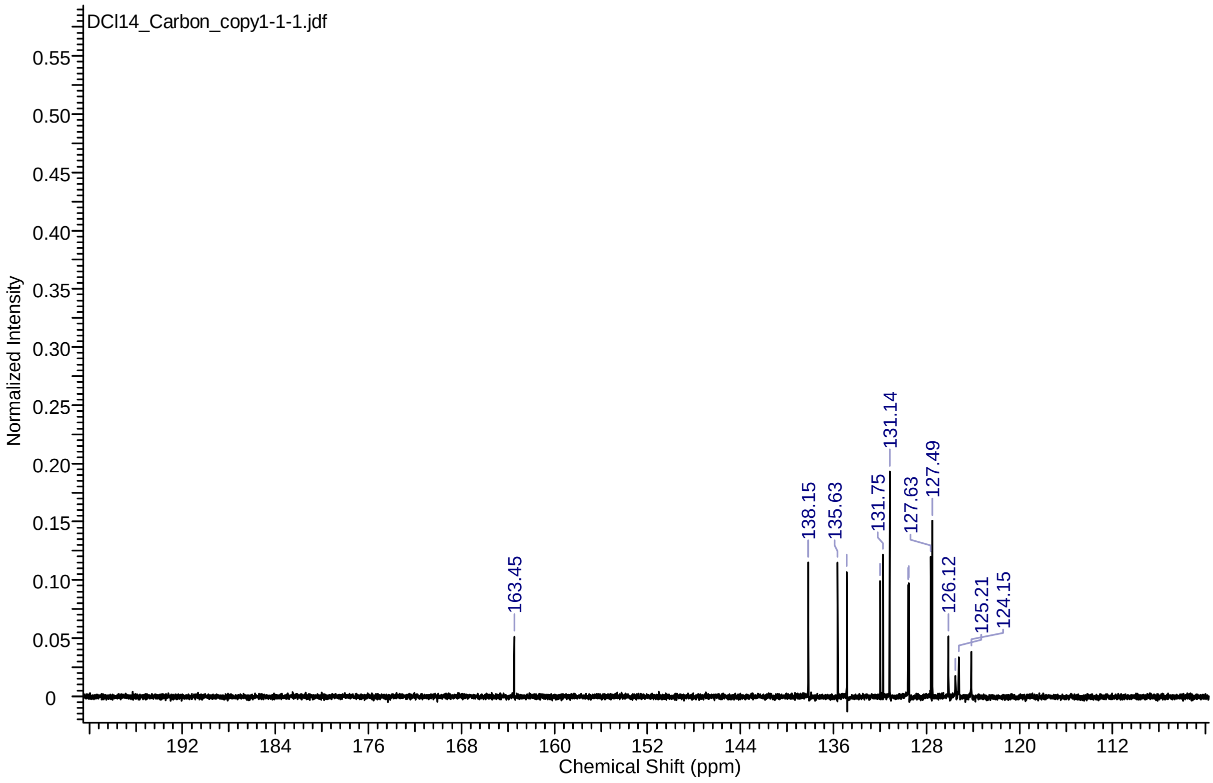

Figure S23. ^{13}C-NMR (DMSO-d_6) spectrum of (2*E*)-*N*-(2-chlorophenyl)-3-(3,4-dichlorophenyl)prop-2-enamide (**2e**).

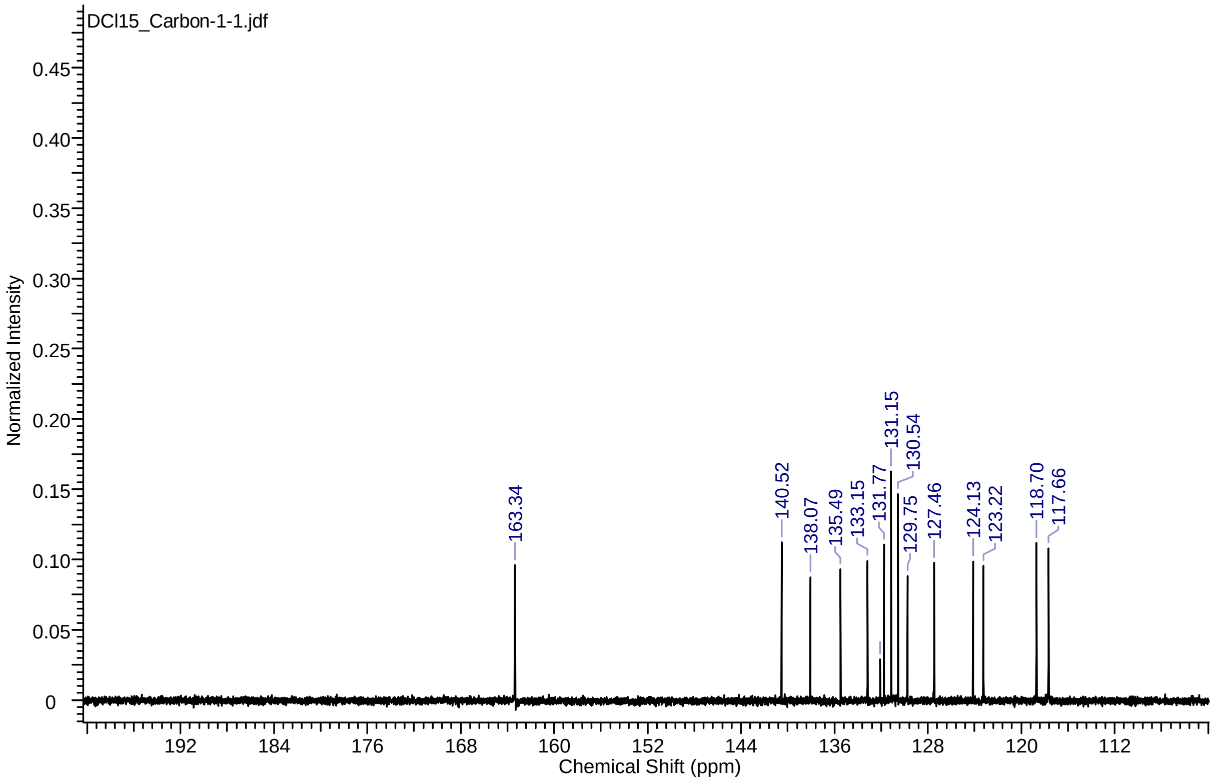

Figure S24. ^{13}C-NMR (DMSO-d_6) spectrum of (2*E*)-*N*-(3-chlorophenyl)-3-(3,4-dichlorophenyl)prop-2-enamide (**2f**).

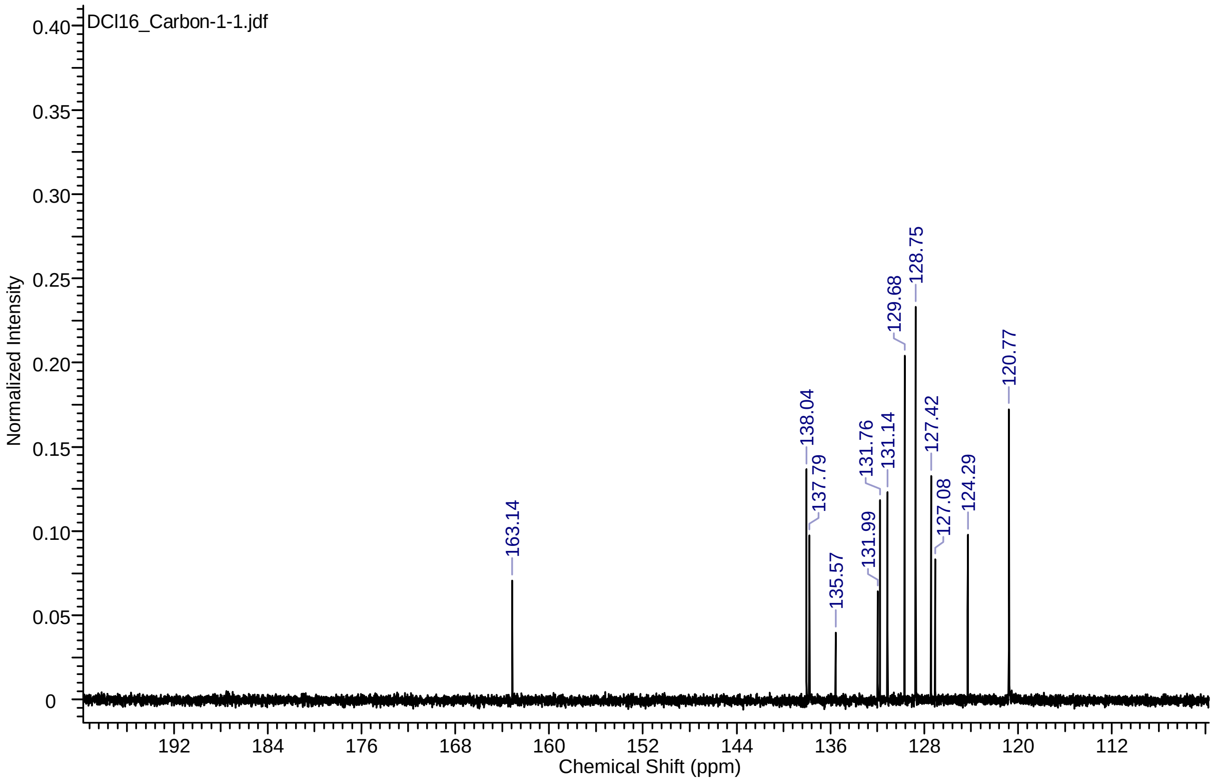

Figure S25. ^{13}C-NMR (DMSO-d_6) spectrum of (2*E*)-*N*-(4-chlorophenyl)-3-(3,4-dichlorophenyl)prop-2-enamide (**2g**).

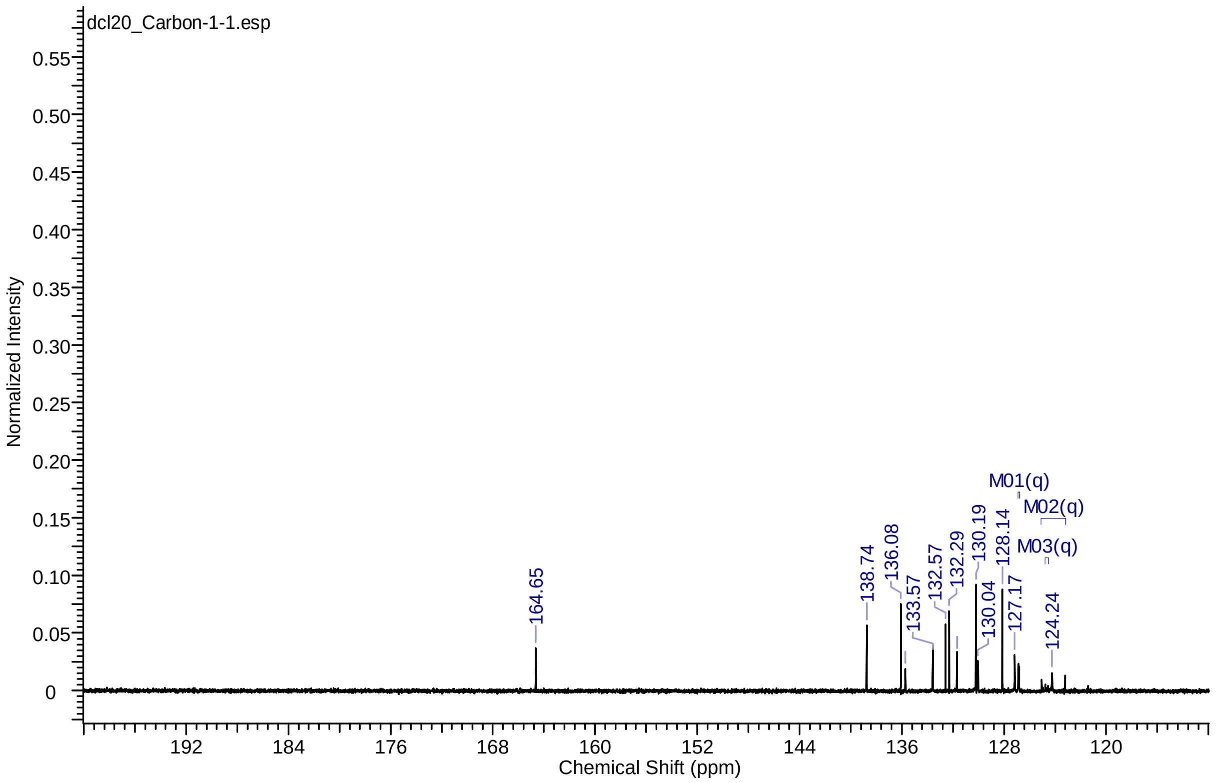

Figure S26. ^{13}C-NMR (DMSO-d_6) spectrum of (2*E*)-3-(3,4-dichlorophenyl)-*N*-[2-(trifluoromethyl)phenyl]prop-2-enamide (**2h**).

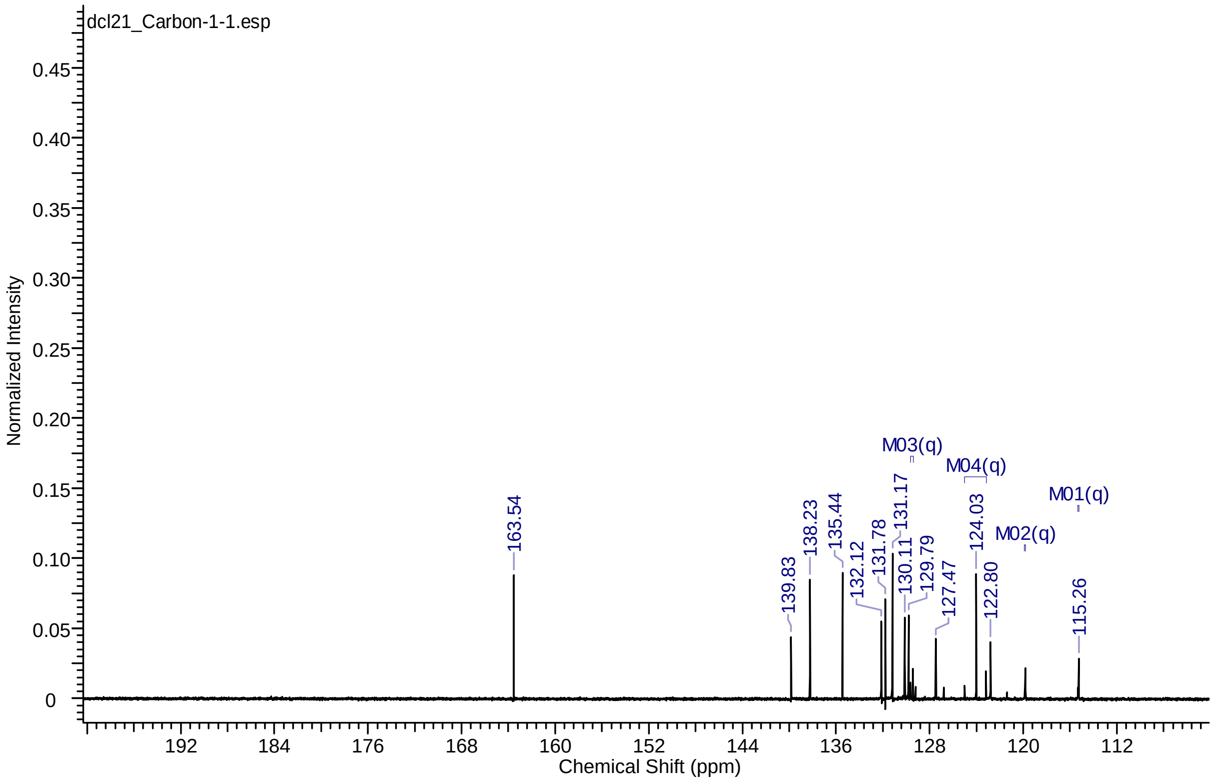

Figure S27. ^{13}C-NMR (DMSO-d_6) spectrum of (2*E*)-3-(3,4-dichlorophenyl)-*N*-[3-(trifluoromethyl)phenyl]prop-2-enamide (**2i**).

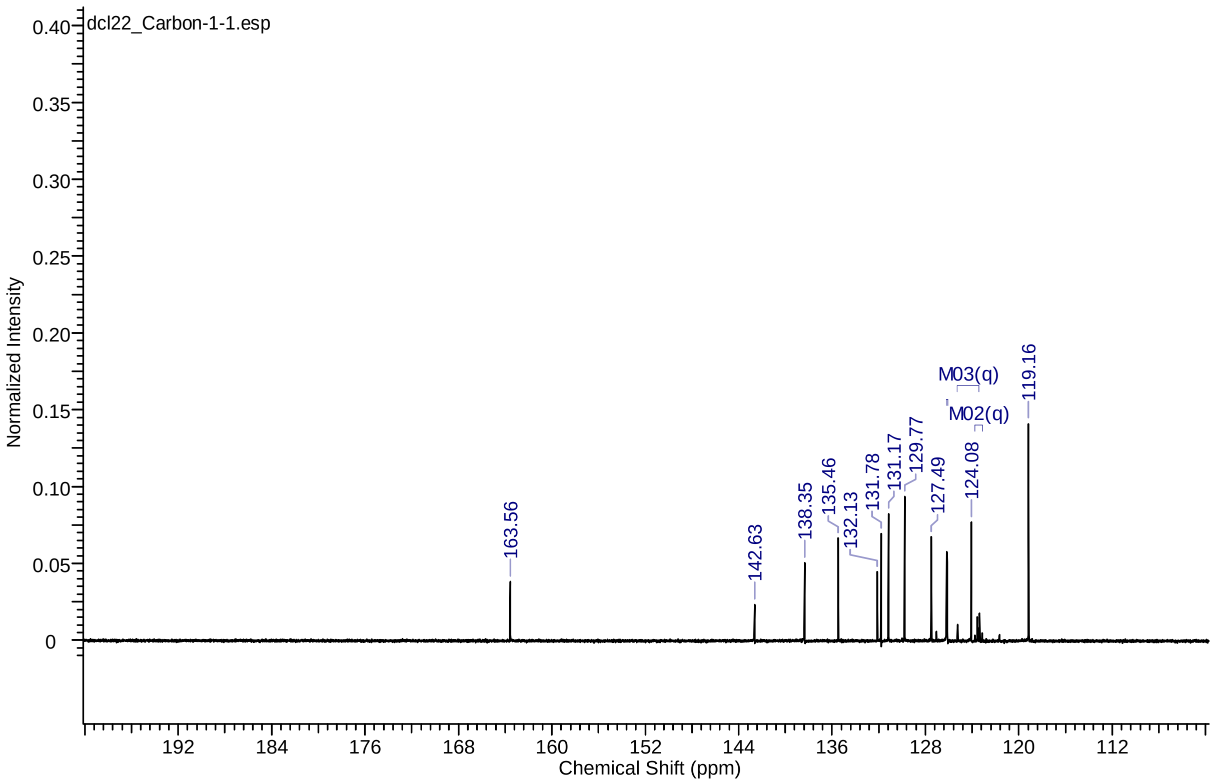

Figure S28. ^{13}C-NMR (DMSO-d_6) spectrum of (2*E*)-3-(3,4-dichlorophenyl)-*N*-[4-(trifluoromethyl)phenyl]prop-2-enamide (**2j**).

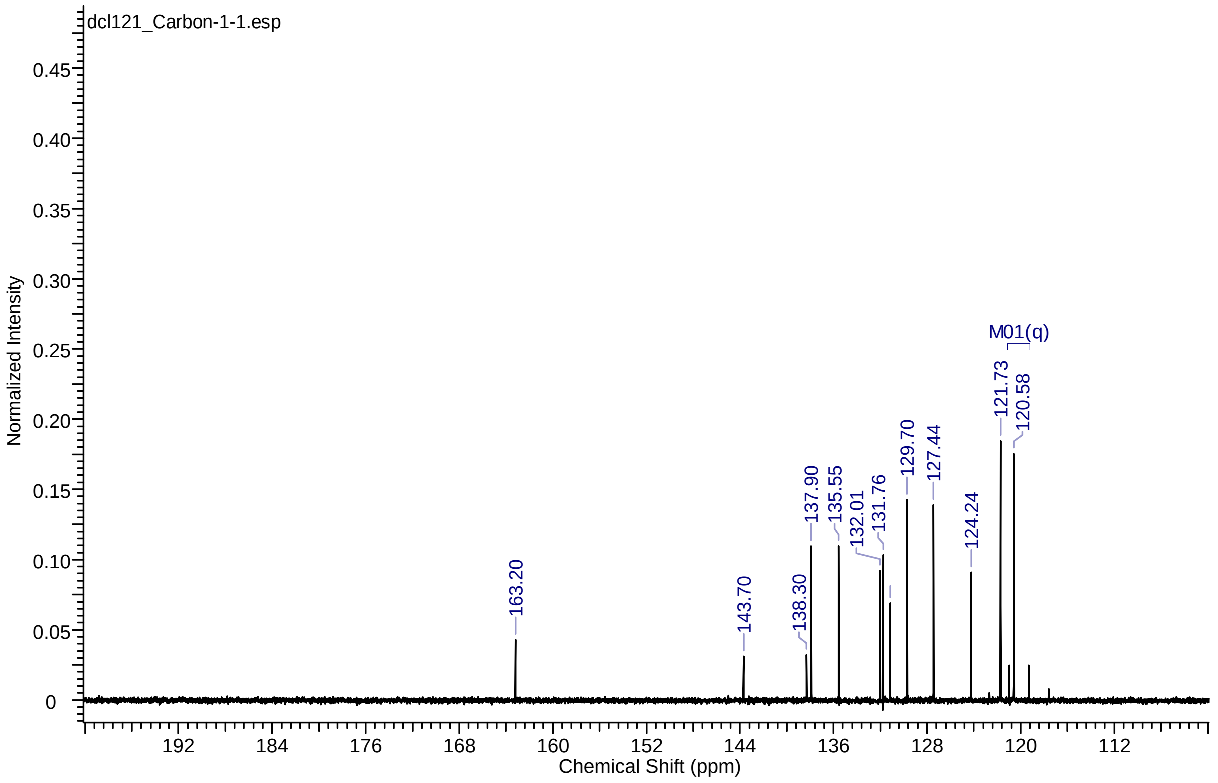

Figure S29. ^{13}C-NMR (DMSO-d_6) spectrum of (2*E*)-3-(3,4-dichlorophenyl)-*N*-[4-(trifluoromethoxy)phenyl]prop-2-enamide (**2k**).

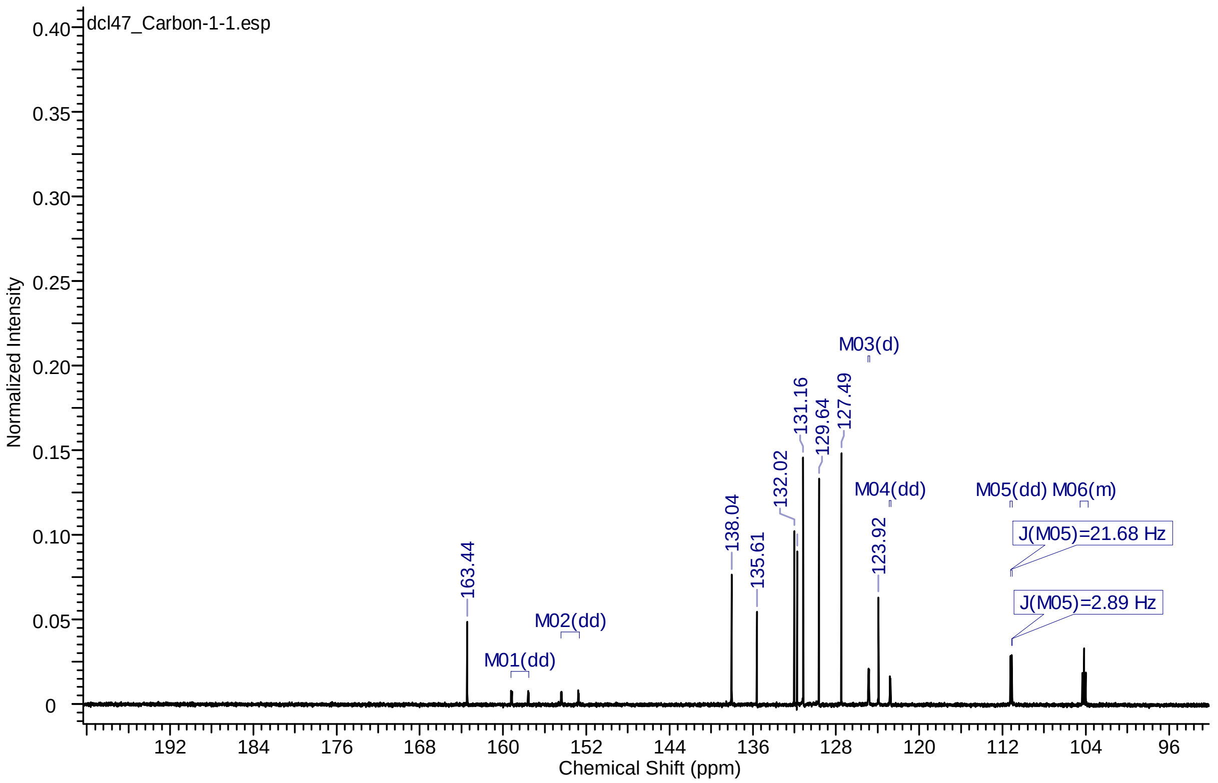

Figure S30. ^{13}C-NMR (DMSO-d_6) spectrum of (2*E*)-3-(3,4-dichlorophenyl)-*N*-(2,4-difluorophenyl)prop-2-enamide (**2l**).

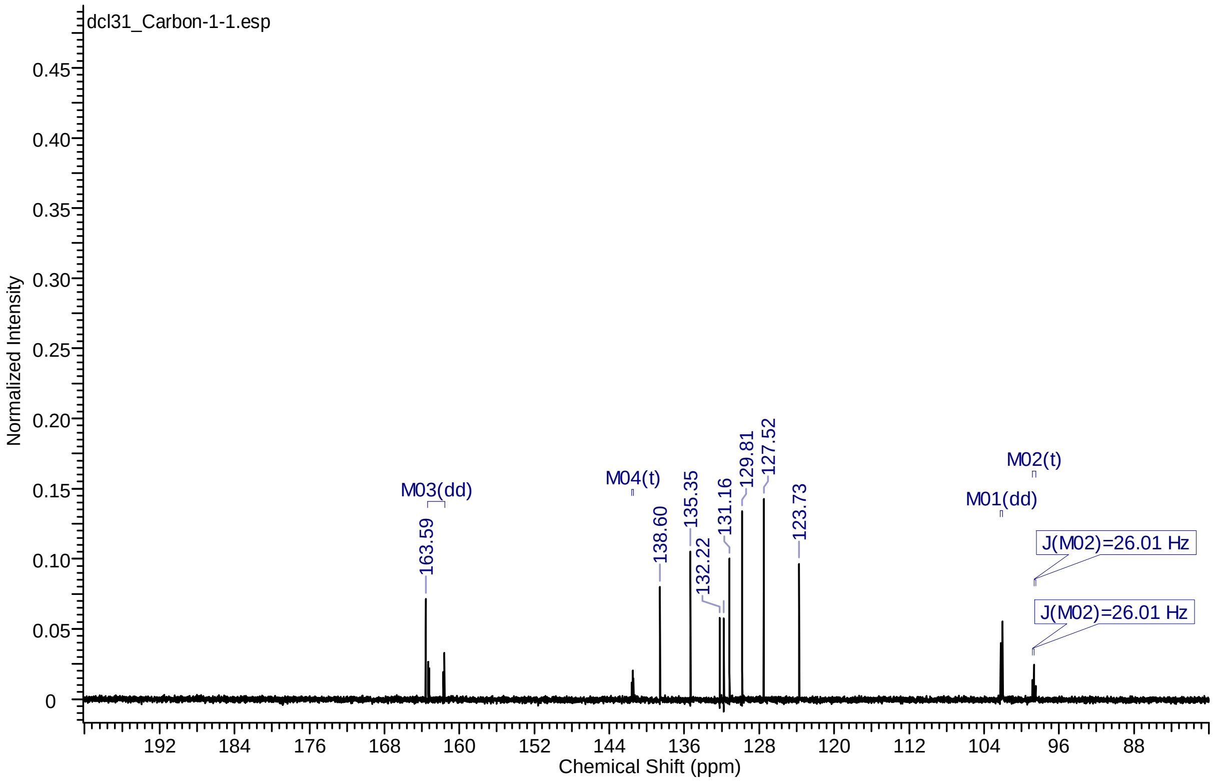

Figure S31. ^{13}C-NMR (DMSO-d_6) spectrum of (2*E*)-3-(3,4-dichlorophenyl)-*N*-(3,5-difluorophenyl)prop-2-enamide (**2m**).

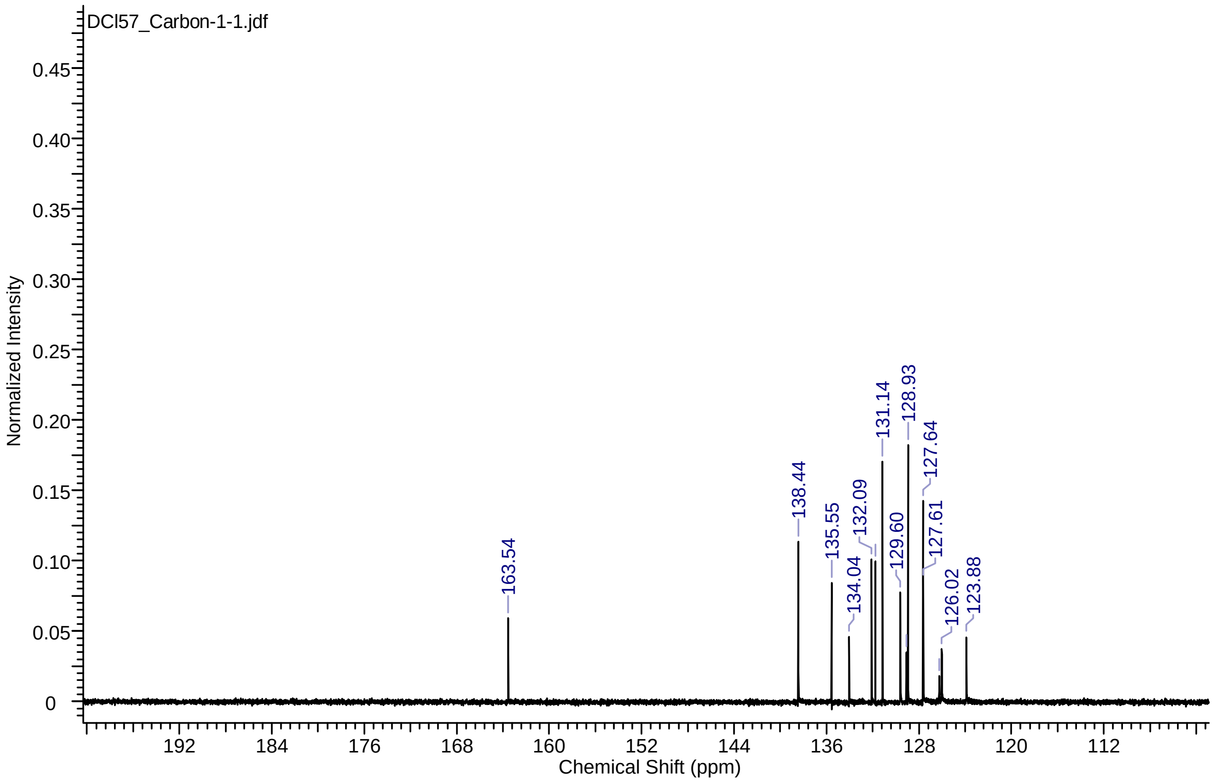

Figure S32. ^{13}C-NMR (DMSO-d_6) spectrum of (2*E*)-*N*-(2,4-dichlorophenyl)-3-(3,4-dichlorophenyl)prop-2-enamide (**2n**).

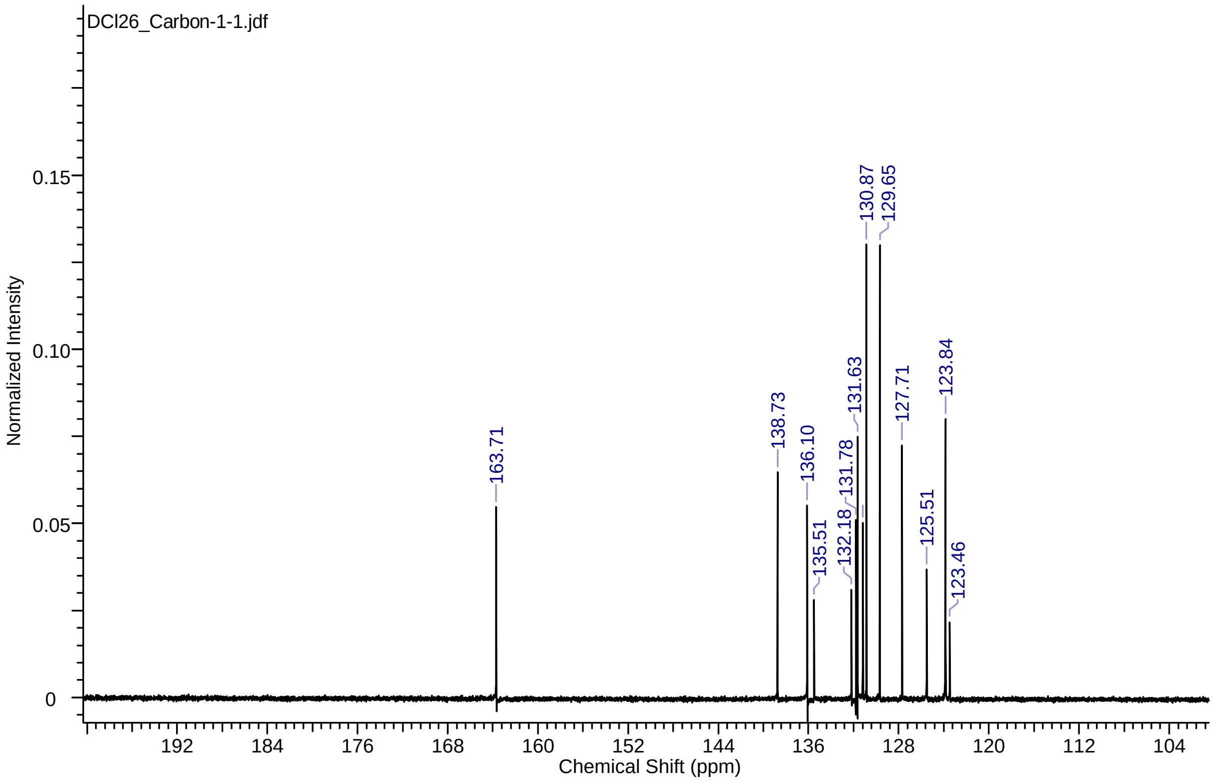

Figure S33. ^{13}C-NMR (DMSO-d_6) spectrum of (2*E*)-*N*-(2,5-dichlorophenyl)-3-(3,4-dichlorophenyl)prop-2-enamide (**2o**).

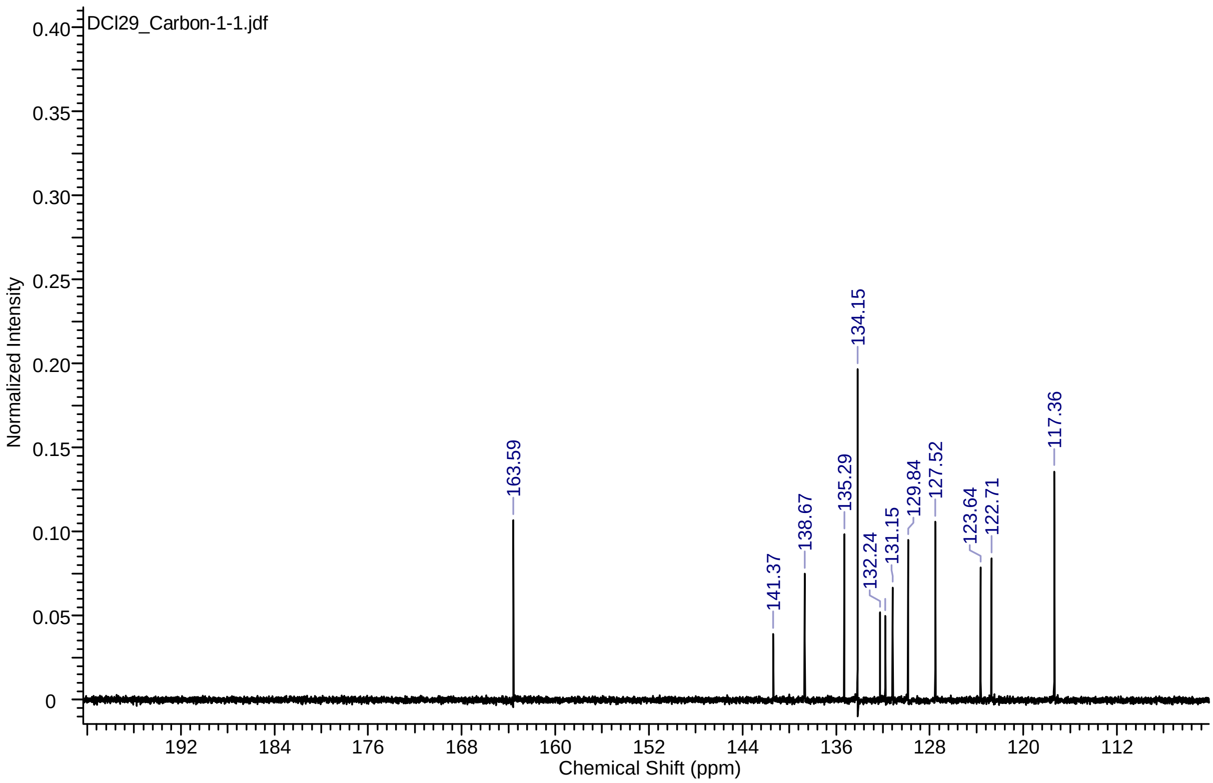

Figure S34. ^{13}C-NMR (DMSO-d_6) spectrum of (2*E*)-*N*-(3,5-dichlorophenyl)-3-(3,4-dichlorophenyl)prop-2-enamide (**2p**).

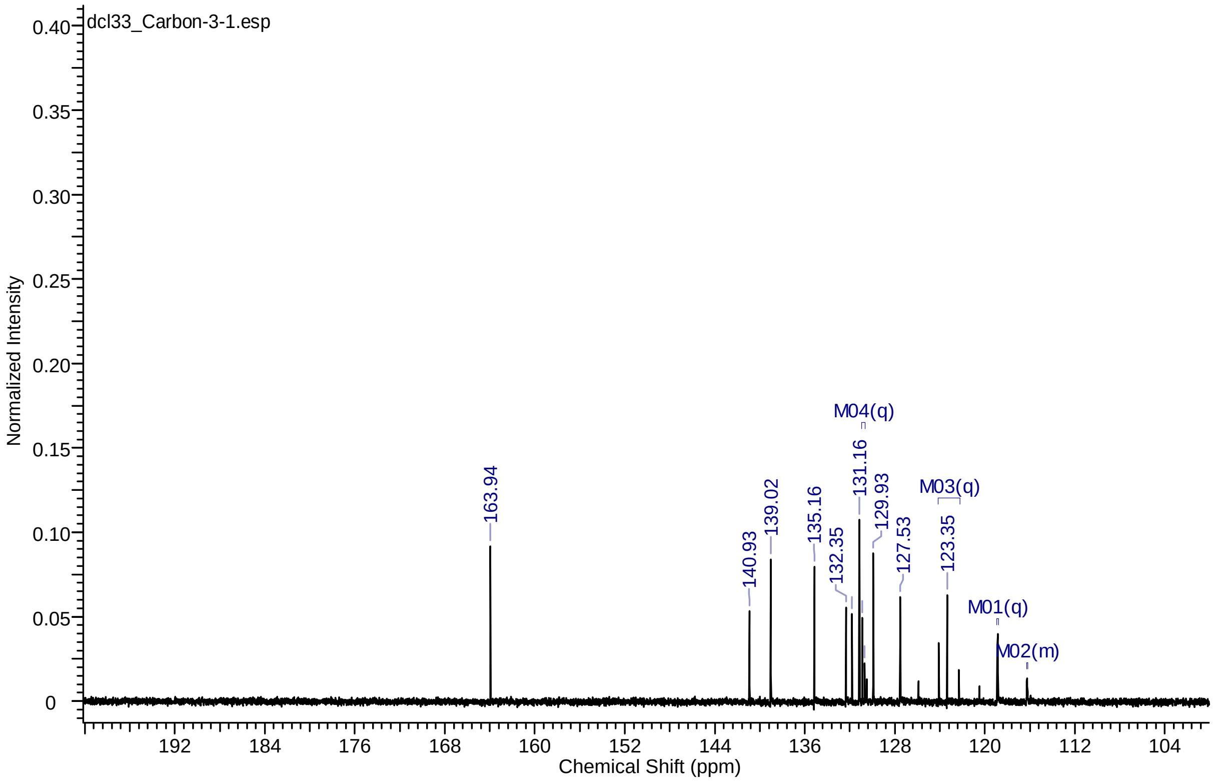

Figure S35. ^{13}C-NMR (DMSO-d_6) spectrum of (2*E*)-*N*-[3,5-bis(trifluoromethyl)phenyl]-3-(3,4-dichlorophenyl)prop-2-enamide (**2q**).

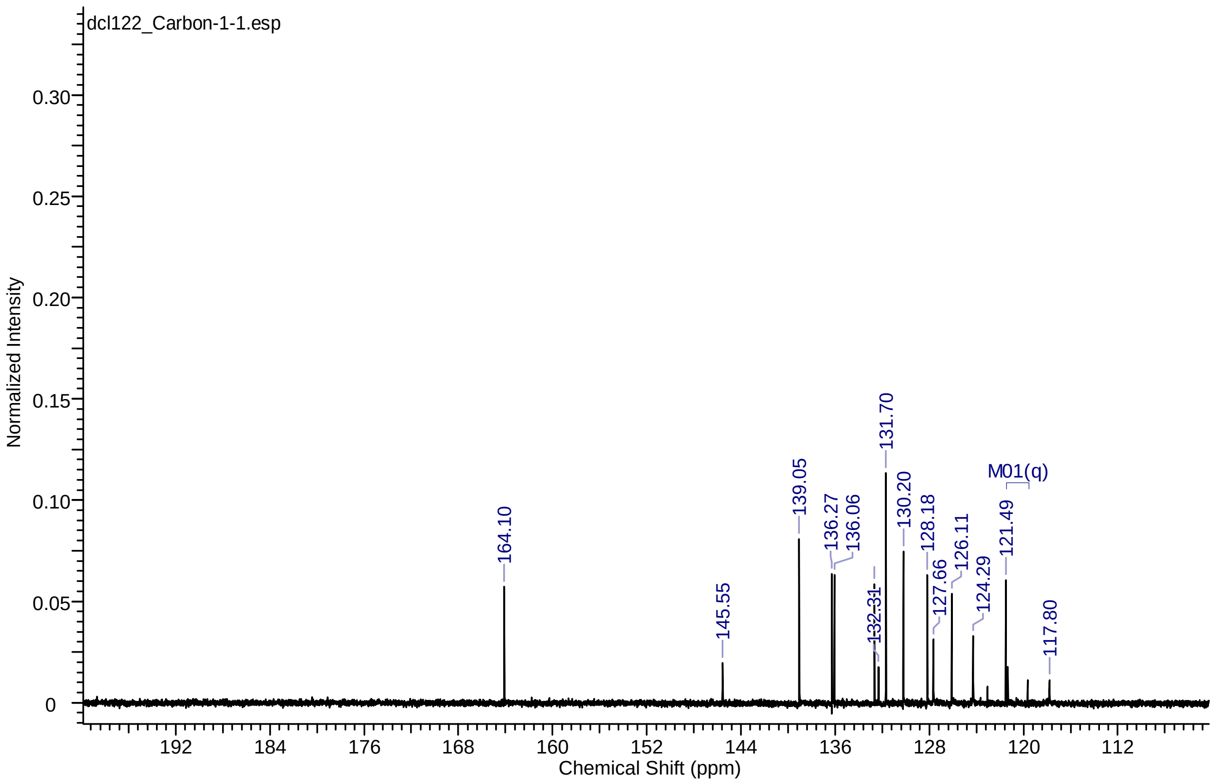

Figure S36. ^{13}C-NMR (DMSO-d_6) spectrum of (2*E*)-*N*-[2-bromo-4-(trifluoromethoxy)phenyl]-3-(3,4-dichlorophenyl)prop-2-enamide (**2r**).
